# Fast and Efficient Root Phenotyping via Pose Estimation

**DOI:** 10.34133/plantphenomics.0175

**Published:** 2024-04-12

**Authors:** Elizabeth M. Berrigan, Lin Wang, Hannah Carrillo, Kimberly Echegoyen, Mikayla Kappes, Jorge Torres, Angel Ai-Perreira, Erica McCoy, Emily Shane, Charles D. Copeland, Lauren Ragel, Charidimos Georgousakis, Sanghwa Lee, Dawn Reynolds, Avery Talgo, Juan Gonzalez, Ling Zhang, Ashish B. Rajurkar, Michel Ruiz, Erin Daniels, Liezl Maree, Shree Pariyar, Wolfgang Busch, Talmo D. Pereira

**Affiliations:** Salk Institute for Biological Studies, La Jolla, CA 92037, USA.

## Abstract

Image segmentation is commonly used to estimate the location and shape of plants and their external structures. Segmentation masks are then used to localize landmarks of interest and compute other geometric features that correspond to the plant’s phenotype. Despite its prevalence, segmentation-based approaches are laborious (requiring extensive annotation to train) and error-prone (derived geometric features are sensitive to instance mask integrity). Here, we present a segmentation-free approach that leverages deep learning-based landmark detection and grouping, also known as pose estimation. We use a tool originally developed for animal motion capture called SLEAP (Social LEAP Estimates Animal Poses) to automate the detection of distinct morphological landmarks on plant roots. Using a gel cylinder imaging system across multiple species, we show that our approach can reliably and efficiently recover root system topology at high accuracy, few annotated samples, and faster speed than segmentation-based approaches. In order to make use of this landmark-based representation for root phenotyping, we developed a Python library (*sleap-roots*) for trait extraction directly comparable to existing segmentation-based analysis software. We show that pose-derived root traits are highly accurate and can be used for common downstream tasks including genotype classification and unsupervised trait mapping. Altogether, this work establishes the validity and advantages of pose estimation-based plant phenotyping. To facilitate adoption of this easy-to-use tool and to encourage further development, we make *sleap-roots*, all training data, models, and trait extraction code available at: https://github.com/talmolab/sleap-roots and https://osf.io/k7j9g/.

## Introduction

Plant roots play a pivotal role in the survival and growth of nearly all terrestrial plants. They are responsible for water and nutrient uptake, anchoring the plant, and can serve as storage organs. Many of these functions are intimately linked to the spatial distribution of roots within a root system in the soil. This spatial configuration is defined as root system architecture (RSA). RSA is indicative of the volume of soil that is explored by a root system and the root surface area that is the interface between roots and soil. RSA is therefore crucial for the uptake of nutrients and water, which are resources that are distributed non-uniformly in the soil. RSA is therefore linked to plant survival and productivity. For example, deep rooting is associated with drought tolerance in several crop species [1–3] and shallow roots are associated with higher productivity under low phosphate conditions, which is a nutrient localized in the topsoil [4]. Given these crucial functions, roots are key players in stress resilience and adaptation to varying environments. Their ability to transfer carbon into the soil [5] positions them at the nexus of climate change and its mitigation. It is thought that enhancing plant root traits that lead to more of their fixed carbon being stored in the soil for a longer time can contribute to substantial mitigation of climate change [6]. To identify genes and genetic variants that can be harnessed to develop plants with superior root systems, and to pinpoint existing plant varieties with these traits, large-scale phenotypic screens of plants and their RSA are necessary [7]. Using the resulting data can be utilized for genetic improvement of crop species to increase root system performance to enhance important characteristics such as stress tolerance or resource use efficiency and carbon sequestration [8,9]. However, large-scale phenotyping of RSA is challenging as roots are hidden in the soil and when excavated do not retain their three-dimensional (3D) structure.

High-throughput plant phenotyping systems allow for the acquisition of very large image datasets. For instance, the Root Architecture 3-D Imaging Cylinder (*RADICYL*) high-throughput gel system for root phenotyping allows us to capture images of the 3D RSA of hundreds of plants daily using machine vision cameras and an automated rotating stage [10]. The system facilitates acquiring comprehensive and detailed images during the early stages of plant development (typically up to 2 weeks, depending on the crop/variety) while maintaining a relatively short overall experiment duration. Our primary objective is to identify and characterize young root phenotypes at both individual and root system levels with predictive power for those of mature plants. This technique can be used in tandem with lower-throughput methods, such as those involving soil or older plants, to validate findings [11]. It empowers low-cost, high-throughput phenotyping in a controlled environment [12–14]. Additionally, the full characterization of the RSA in this pipeline will be useful for genome-wide association studies (GWASs) where correlations between genotypic and phenotypic variations can be quantified and can lead to the identification of genes and their variants that determine RSA [15].

In order to automate the analysis of root images and subsequent RSA phenotypic trait extraction, most previous approaches have relied on image segmentation [16]. This technique first separates foreground plant root pixels from the background, requiring time-consuming manual adjustment of thresholding parameters and regions of interest in order to achieve reliable segmentation [17–19]. Once segmentation masks are obtained, “skeletonization” can be applied, an algorithm that recovers the center line of the plant roots that can be used to extract geometric (e.g., root lengths) and topological (e.g., branching angles) traits [20]. This process is highly sensitive to errors in the image segmentation step as even minor failures may result in fragmented or merged roots. While this issue is tolerable in smaller-scale settings where manual or heuristic correction is feasible, it can quickly become intractable in large-scale screens or when working with multiple species due to variability in imaging conditions and morphology.

The adoption of deep learning has drastically improved the accuracy of root segmentation [16,21–25]. Despite these advances, two issues remain: (a) annotation for segmentation is intrinsically laborious as pixels must be carefully annotated and (b) downstream skeletonization and instance segmentation have a very low tolerance to errors. Recent approaches have been developed to mitigate the annotation labor issue by using human-in-the-loop annotation (e.g., RootPainter [24]) or synthetic data [26]. These are still subject to the skeletonization and instance segmentation challenges, however. To address these, other methods have resorted to object detection [27], which does not reconstruct the entire RSA; image statistics description [28], which does not provide morphological feature localization necessary to extract some ordinal and spatial traits (e.g., root counts); or hybrid architectures that combine segmentation and landmark detection [21,29], which still suffer from the same issues as the segmentation-only approaches.

Recently, it has been demonstrated that multi-instance pose estimation, a technique for landmark localization and grouping, can be used to directly extract plant morphology in time-lapse imagery [30]. Unlike hybrid approaches [21], pose estimation is completely segmentation-free, thereby circumventing the issues of annotation labor and sensitivity to errors. Despite these advantages, pose estimation-based approaches have not been systematically evaluated in the context of plant RSA phenotyping, in part due to the lack of tooling for extracting meaningful traits from the positional data afforded by pose estimation.

Here, we describe a pipeline for plant root pose estimation (segmentation-free landmark detection and grouping) and downstream RSA trait extraction. For pose estimation, we leveraged SLEAP (Social LEAP Estimates Animal Poses), a deep learning-based framework for landmark detection and grouping originally developed for animal motion capture [31]. As most RSA trait estimation tools are designed for segmentation-based inputs, we also developed *sleap-roots*, a Python-based package for pose-based RSA trait extraction, which produces up to 1,035 traits per plant. We applied our approach to a range of plant species, including crop plants such as soybean *(Glycine max*; Movie S1), rice (*Oryza sativa*; Movie S2), canola (*Brassica napus*; Movie S3), and the model plant Arabidopsis (*Arabidopsis thaliana*; Movie S4). Our results show that our approach is highly accurate (0.3 mm to 2.3 mm root landmark localization error) and efficient (peak accuracy at 10 to 200 labeled images), depending on the morphological complexity of the plant. We find that our method outperforms segmentation-based approaches in terms of annotation speed (~1.5× faster), training (~10× faster), and prediction (~10× faster), at the same or better accuracy. We further validate the correctness of our trait extraction pipeline, demonstrating that it is highly accurate when compared to Fiji-based manual trait annotation (*R*^2^ = 0.980 to 0.998) or traits computed from manually proofread landmarks (up to 99.5% of data points within 1 SD). Finally, as a proof of principle, we show that the traits derived from our pipeline can be used for genotype classification and unsupervised phenotypic trait space visualization. We make all code, labeled data, and trained models available at: https://github.com/talmolab/sleap-roots and https://osf.io/k7j9g/.

## Materials and Methods

### Overview

**Fig. 1.**
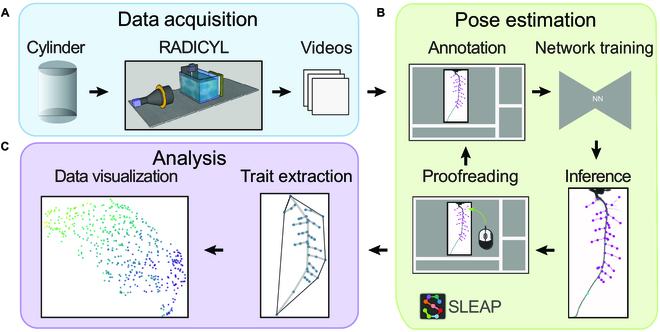
Overview of the high-throughput phenotyping pipeline using SLEAP. (A) Plant cultivation in cylinders and RADICYL imaging system, with image compression into HDF5 format. (B) Importing and annotating videos in SLEAP for neural network (NN) training in root pose estimation, followed by user refinement of NN-predicted labels. (C) Extraction of root traits from corrected predictions for enhanced machine learning analysis, allowing comprehensive trait quantification across plants.

We developed a root trait extraction pipeline specifically designed for images from the *RADICYL* phenotyping system, utilizing the SLEAP software for pose estimation (Fig. 1). The plants were cultivated in 3D, controlled environments using transparent plastic cylindrical containers, which are referred to as “cylinders.” Seventy-two images for each plant were captured in 5° steps, for a comprehensive 360° view of the root system within the transparent medium. Following the imaging, the resultant images were compressed into Hierarchical Data Format, version 5 (HDF5) files [32]. This provided both portability and easy importation into the SLEAP software.

**Table 1.** Summary of experimental conditions for each crop

Crop	Experimental duration	Photoperiod (daytime hours)	Average daytime temperature (°C)	Average nighttime temperature (°C)	Average daytime relative humidity (%)	Average nighttime relative humidity (%)
Soybean	May–August 2021	13	26.59	22.26	57.80	68.21
Rice	April–May 2022	13	33.81	25.52	44.65	65.54
Canola	September–November 2022	16	22.65	18.56	63.02	76.34

The SLEAP workflow incorporated an iterative process of labeling, training, and correction (https://sleap.ai/tutorials/tutorial.html). This was designed to establish a diverse training set and to enable accurate identification of root instances. Following the initial predictions, the results were reviewed and refined within the SLEAP graphical user interface (GUI), which we call “proofreading.”

Two root trait extraction pipelines were made: One is designed for younger monocot plants with one primary and crown roots, and the other is for dicot plants with one primary and lateral roots. The younger monocot pipeline computes 102 traits per frame and a total of 918 derived traits per plant. The dicot pipeline computes a total of 115 traits per frame and a total of 1,035 summary traits per plant. Outliers were identified by employing principal components analysis (PCA) [33] and the Mahalanobis distance [34]. We utilized the high-dimensional dataset for genotype classification using the Random Forest algorithm [35] and SHAP (SHapley Additive exPlanations) [36] for feature selection, aimed at identifying heritable traits that could effectively differentiate genotypes. Unsupervised exploratory data analysis techniques, such as PCA, and Uniform Manifold Approximation and Projection (UMAP) [37] were implemented to visualize the phenotypic trait space.

### Plant cultivation

#### Seed collections

To train and evaluate the potential applications of SLEAP, we prepared datasets of cylinder images from several dicot species—soybean (*G. max*), canola (*B. napus*), and Arabidopsis (*A. thaliana*)—and one monocot species, rice (*O. sativa* ssp. *japonica*). These species were chosen due to their distinct early RSAs to validate the versatility and robustness of SLEAP across varied morphologies.

For rice, we employed fast-neutron-induced (FN) mutant Kitaake strains provided by the laboratory of P. Ronald at the University of California, Davis [38]. The FN irradiation process results in a wide spectrum of mutations, making these strains ideal for constructing a diverse training set [39]. The soybean lines were sourced from a genetically diverse United States Department of Agriculture (USDA) strain collection maintained by the laboratory of H. Nguyen at the University of Missouri [40]. Canola strains were obtained from a diversity panel from the laboratory of M. Stamm at Kansas State University [41]. Arabidopsis seeds were sourced from the TRANSPLANTA collection, developed for the inducible expression of transcription factors [42]. These collections of seeds have publicly available genetic information, making the trained models valuable for future genetic studies such as GWAS.

#### Environmental conditions

The crop plants—soybean, rice, and canola—were cultivated in a greenhouse located in Encinitas, CA. The plants were organized using a completely randomized block design, and a barcode system was employed to monitor and track each individual plant throughout the study (Table 1). Arabidopsis plants were grown in growth chambers at a temperature of 21 °C during the daytime and 16 °C during the nighttime, with a photoperiod of 16 daytime hours and relative humidity of 60%.

#### Soybean

Soybean seeds underwent vapor-phase sterilization in an airtight container for 16 h. This was achieved using a sterilizing solution composed of 200 ml of 8.25% bleach and 3.5 ml of 1 M HCl. The solution was agitated on a stir plate within the container to produce 0.0035 mol of Cl2 gas. The plants were cultivated in a medium consisting of ^1^/_2_ strength Murashige and Skoog (MS) medium (Sigma-Aldrich, St. Louis, USA) and 0.8% Phytagel (Sigma-Aldrich, St. Louis, USA) with a pH of 5.7. Gel cylinders were filled with 170 ml of media and were allowed to solidify at room temperature for 2 h and could be stored at 4 °C for up to 3 weeks. In sterile conditions, the sterilized seeds were sown approximately 1 mm beneath the gel media’s surface. A small hole was created in the gel to accommodate each seed. The hilum of the seed was oriented downward in the gel. The plants were then transferred to the greenhouse for growth. Imaging of the cylinders was conducted 6 days after germination (DAG).

#### Rice

The seeds were first dehusked and then sterilized in a 2.475% bleach solution for 30 min. After sterilization, they were rinsed three times with sterile water and soaked in sterile water for an additional 5 min. The water was then drained, and the sterilized seeds were carefully transferred to wet filter paper placed on sealed germination plates, ensuring that all procedures were conducted under sterile conditions. These seeds were incubated on the wet filter paper at 28 °C for 2 days. Seedlings of a similar developmental stage were selected and cultivated in cylinders using the same protocol as described for soybean, with the radicle oriented downward. Rice plants were cultivated in the greenhouse and grown for 10 DAG with the day of planting in the cylinders considered as the starting point. Imaging of the cylinders was conducted at both 3 DAG and 10 DAG.

#### Canola

Canola seeds underwent vapor-phase sterilization following the same protocol as soybean. Subsequently, the seeds were liquid-phase sterilized following the same protocol as rice. The water was then replaced, and the seeds were stored at 4 °C for 72 h. Next, seeds were sown on plates filled with 45 ml of 0.01% plant preservative mixture (PPM) (Caisson Labs, Smithfield, USA) in Hoagland growth media. The medium consisted of ^1^/_2_ strength Hoagland (MP Biomedicals, Solon, USA) and 1.0% Phytagel, adjusted to a pH of 5.8. Pregermination took place on these plates in the greenhouse over a span of 24 to 48 h. Once the root tip emerged from the seed coat, the germination date was noted, and the seedling was transferred to a cylinder under sterile conditions. This time point was marked as 0 (DAG). For planting, a small hole was created in the gel medium, allowing for half of the seed to be submerged. While ensuring sterility, the seedlings were planted with their root tips oriented downward in the cylinders. Canola plants imaged from 5 to 13 DAG were included in the training set.

#### Arabidopsis

Arabidopsis seeds underwent a stratification process for 48 h at 4 °C. For sterilization, we employed vapor-phase sterilization using a mixture of 200 ml of bleach and 5 ml of concentrated HCl in a sealed container for 1 h. Seeds were pre-germinated on plates and subsequently grown in cylinders in a medium containing ^1^/_4_ strength MS, 1% sucrose, and 1% Phytagel, adjusted to a pH of 5.7. Imaging of the cylinders took place 7 days after planting in cylinders.

### Data acquisition

The cylinder imaging system has a Basler acA2000-50gm GigE camera, which produces gray-scale images with a resolution of 2,048 px × 1,088 px. The camera is equipped with a 0.093×, 2/3″ C-Mount TitanTL telecentric lens and placed so that the plant is in the field of view and directly in front of the camera. The cylinder is placed on a rotating stage inside of an aquarium that has a constant backlight with a red filter. The stage rotates 5°, 72 times. An image is captured at every new angle, resulting in 72 frames per plant. The camera, aquarium, and backlight are mounted on an optical breadboard for consistent positions. The rate of image acquisition is 72 frames/7 s. Each image is 2,165 KB. The scale is 10.6 px/mm.

Cylinders are 110 mm high with a diameter of 68 mm (VWR International, Radnor, USA) and filled with 170 ml of media. Cylinders are made of clear polystyrene.

### Data annotation

For each plant, the set of 72 images was compressed utilizing lossless GZIP compression, set at a compression level of 1, into the HDF5 file format. To ensure unbiased annotation, each HDF5 file was named based on the unique barcode identifying each cylinder. This naming convention not only facilitated the alphabetical arrangement of plants but also shielded the annotator from potential biases that might arise from prior knowledge of the plant’s identity or genotype.

For each crop and root type, specific models were trained as detailed in Table 2. To bolster the training set’s diversity, plants were sourced at random from broader screenings of the pertinent seed collections. Root annotation within images was conducted using the SLEAP labeling workflow, in accordance with the guidelines provided in SLEAP’s online documentation (https://sleap.ai). For each dataset, one to three distinct annotators were assigned nonoverlapping subsets of the data to label. For every video, annotators were presented with uniformly distributed labeling suggestions, with 20 labeling suggestions per video. They were directed to annotate two frames for each plant: an initial frame and a mid-time series frame, providing different plant viewpoints. Given that cylinders are symmetrically placed on the stage devoid of any bias, the selection of the first frame was effectively randomized.

**Table 2.** Datasets used for training each model

Model name	Plant species	Root type	Age (DAG)	Number of plants	Number of labeled frames	Skeleton (number of nodes)
Soybean primary roots (5–8 DAG)	*G. max*	Primary	5–8	226	1,389	6
Soybean lateral roots (5–8 DAG)	*G. max*	Lateral	5–8	220	482	4
Canola primary roots (5–13 DAG)	*B. napus*	Primary	5–13	69	335	6
Canola lateral roots (5–13 DAG)	*B. napus*	Lateral	5–13	65	390	3
Arabidopsis primary roots (7 DAG)	*A. thaliana*	Primary	7	25	50	6
Arabidopsis lateral roots (7 DAG)	*A. thaliana*	Lateral	7	131	276	4
Rice primary root (3 DAG)	*O. sativa*	Primary	3	134	748	6
Rice crown roots (3 DAG)	*O. sativa*	Crown, primary	3	193	868	6
Rice crown roots (10 DAG)	*O. sativa*	Crown, primary	10	313	586	6

**Table 3.** Summary of proofread data subsets

Dataset	Number of plants	Number of genotypes	Time points (DAG)
Soybean	78	10	6
Canola	108	10	7
Rice	298	17	3

**Fig. 2.**
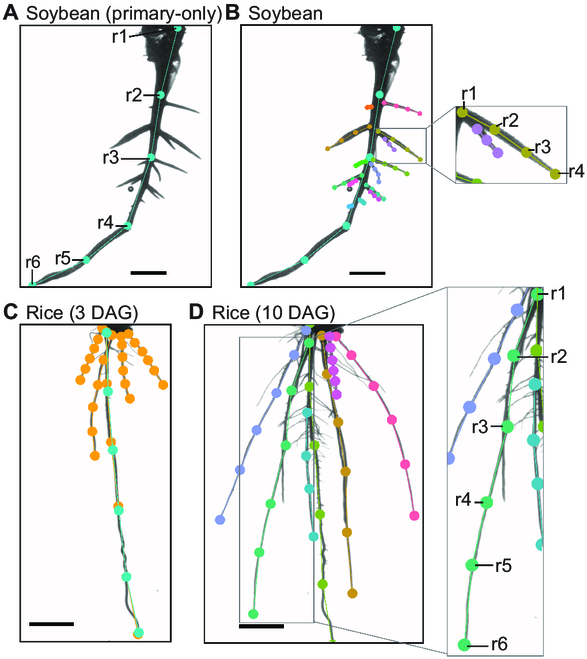
Plant and root-type labeling rules to ensure consistency. (A) Soybean, similarly to the other dicots, has a prominent primary root that can be labeled from beginning to end. (B) Lateral roots are frequently occluded and require the visibility of the first node to be toggled off. Rice roots have crown roots that are difficult to differentiate from the primary root. For 3 DAG rice roots (C), we labeled a model for all crown and primary roots, shown in orange, and a model only for the primary root, shown in cyan. (D) Ten DAG suffer from heavy occlusions and primary roots closely resembling crown roots. In this case, only one class was given to all the primary and crown roots and careful labeling rules were employed. Scale bar, 1 cm.

Each visible root was categorized as an individual instance, represented by a tree structure with nodes dictated by the skeleton. This skeleton is a path, where every node is linked to precisely two others, barring the two terminal nodes that had a singular connection (Fig. 2). The nodes are named {*r1*, *r2*, …, *rN*}, with “*N*” signifying the number of nodes in the root’s skeleton. The determination of *“N”* for each crop and root type was approached with care, balancing the trade-off between accuracy and efficiency. Specifically, “*N*” was identified as the minimum number of nodes necessary to aptly depict the root’s curvature. While a higher number of nodes allows for a more precise match to the root, excessive nodes in the skeleton can inadvertently introduce errors and prolong the labeling process. We opted for a consistent number of nodes: six for primary and crown roots, and three to four for lateral roots. The starting point, labeled as *r1*, represents either the root’s commencement or the highest visible point ascending the root. Nodes were evenly spaced along each root, with root tips defining their extremities. If the root tips or bases were not visible within the image, their visibility markers were deactivated. It is important to note that no breaks in root visibility were permitted.

To enhance the precision of model predictions, rigorous labeling protocols were established for each model. In the dicots—soybean, canola, and Arabidopsis—a distinct primary root emerges, which is labeled from its beginning to its end (from *r1* to *rN*) as depicted in Fig. 2A. Portions of slender roots (common in canola and Arabidopsis) reaching the bottom of the cylinder were left unlabeled as they were typically obscured by the bottom. For such instances, the tip, or *rN*, was marked at the final discernible point—where the primary root met the cylinder’s base. Contrarily, the soybean has a thicker primary root that remains visible even at the cylinder’s bottom, facilitating its labeling. It is worth noting that lateral roots often overlap, especially when their branching points are proximate (Fig. 2B). In overlapping scenarios, the more pronounced lateral roots in the frame took precedence, while the visibility of obscured root bases was toggled off. Dicots were primarily evaluated based on their primary and lateral roots to define the RSA, excluding adventitious roots from labeling. This exclusion is due to the fact that a high number of adventitious roots often reflect stress conditions rather than heritable phenotypic traits [43]. Consequently, plants exhibiting excessive numbers of adventitious roots were selectively removed from the collection when feasible.

Rice, being a monocot, presents its own set of challenges. Root architecture of rice is composed of several embryonic and postembryonic root types: the radicle, crown roots, and lateral roots [44]. The first root that grows from a seed is a radicle (called primary root; Fig. 2C), and the four to five embryonic roots emerging from the coleoptilar node are called crown roots (Fig. 2C and D) during the first and second leaf emergence stages. Later, adventitious postembryonic crown roots, also called nodal roots, emerge from the nodes on the stem and tillers [45]. Multiple roots emerge during the embryonic process, leading to complications in labeling and detection due to the overlapping nature of these crown roots at their bases. By 3 DAG, the primary root is easily distinguishable given its length, further characterized by lateral roots branching out. For this stage, the primary and crown roots were collectively labeled under the model titled “Rice crown roots (3 DAG)” in Table 2. Root traits associated with this class of roots are referred to as “main” root traits for brevity. Additionally, the primary root received individual labeling to allow for a more intricate extraction of its specific traits (Fig. 2C). By the time 10 DAG is reached, the distinction between primary and crown roots becomes subtler since most roots have now extended to the cylinder’s base with lateral roots (roots that emerged from primary and crown roots) sprouting from them (Fig. 2D). As a result, one unified model was created for both primary and crown roots, designated as “Rice crown roots (10 DAG)” in Table 2. For both the 3 DAG and 10 DAG crown root models, any base occlusion of multiple roots was addressed either by designating the root’s base as the most visible point on its upward trajectory or by deactivating the visibility of the base nodes. In the “Primary root 3 DAG” model, annotators emphasized marking the primary root up to its genuine base.

To ensure uniformity and facilitate labeling, comprehensive annotation protocols coupled with illustrative images of labeled plants were distributed among all annotators for every project. These resources are made available in the Supplementary Materials.

After each annotation session, the model was either trained independently or integrated with other labeling projects before training. This approach enabled prediction-assisted annotation, which informed the annotator of the model’s error patterns, identified gaps in the training dataset, and reduced annotation time as the dataset grew. In the SLEAP GUI, prediction scores were utilized to swiftly pinpoint and rectify inaccurate predictions.

### Pose estimation

We use the bottom-up approach of pose estimation implemented in SLEAP to perform landmark-based localization of each root. The bottom-up approach was chosen as it has been demonstrated to be successful in localizing plant landmarks with few labels [30]. In contrast to SLEAP’s top-down approach, which performs better with locally distinguishable morphological features, bottom-up models explicitly learn the relationship between landmarks [31]. This is especially helpful in this application as individual landmarks along the length of the root may be better identified using their geometry as opposed to local appearance.

**Fig. 3.**
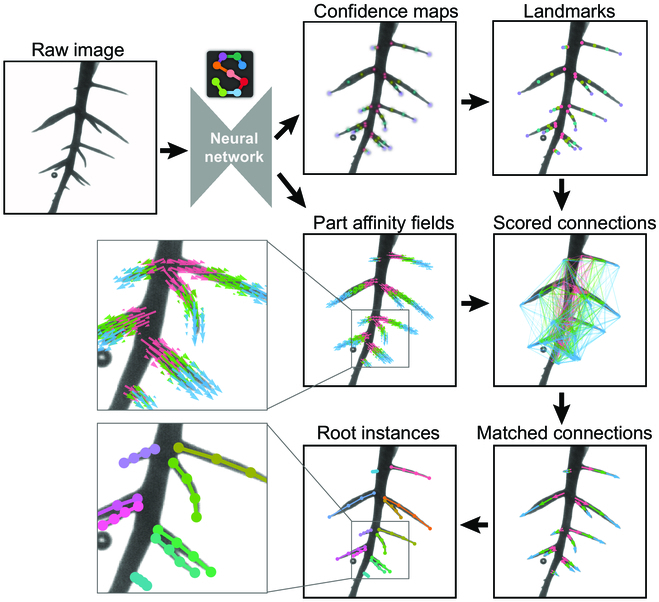
Demonstration of the bottom-up method for root pose estimation. A detailed image of a root system cultivated in a clear gel cylinder is input into a trained NN via SLEAP. This network concurrently produces confidence maps and part affinity fields. The confidence maps, with distinct color codes for each landmark type (*r1* in pink, *r2* in yellow, *r3* in green, *r4* in purple), pinpoint probable root landmark positions. These are then sharpened into discernible peaks based on the highest probabilities within each map. Part affinity fields encode part-to-part associations in 2D vector fields across the image. Using these data, connections between root landmarks are evaluated and scored. The most highly scored connections serve to pair the root landmarks, culminating in the assembly of individual root instances, each distinguished by a unique color.

The root landmarks used are branch points, root tips, and equally spaced nodes in between the beginning and end of each root. Models take full-resolution raw images as input, which are processed by a deep neural network to predict multi-part confidence maps. The local peaks of the confidence maps define the locations of the landmarks (Fig. 3, top). The neural network simultaneously predicts part affinity fields (PAFs)—vector fields defined between connected landmarks [46]. PAFs enable scoring of connections between landmarks, which are then used to match landmarks belonging to the same root (Fig. 3, middle). After forming all possible connections from detected landmarks, the assembled sets of detections form instances of distinct roots (Fig. 3, bottom).

All models were trained using the same hyperparameters using SLEAP v1.3.0. Neural networks used a UNet-like architecture with 3.8 million trainable parameters, 6 downsampling blocks, 5 upsampling blocks, and 24 initial convolutional filters, expanding/contracting at a rate of 1.5 per block (respectively). Downsampling blocks apply two convolutions with ReLU activations followed by max pooling with a stride of two. Upsampling blocks use bilinear interpolation with two convolutions with ReLU activations. All convolutional layers (including pooling) use a kernel size of three. The max stride of the model is 64, with a final output stride of 2 for confidence maps and 8 for PAFs. Both heads are weighed equally and trained against a mean squared error loss. Optimization is performed using the Adam optimizer with AMSgrad enabled. The only augmentations applied were random rotations of −5° to 5°; however, images are always downscaled by a factor of 0.5 (1,024 × 544 final size) at both training and inference time. Models were trained with a batch size of four images per step with an initial learning rate of 1 × 10^−4^, which was reduced by a factor of 0.5 once the validation loss failed to decrease by at least 1 × 10^−6^ for at least five epochs, down to a minimum learning rate of 1 × 10^−8^. Training continued for up to 200 epochs, where an epoch is defined as the number of batches in the training set or 200 batches, whichever is larger, repeating training set images if there were fewer than 200. Shuffling was applied at the image level to produce diverse batches of images. Training was terminated early once the validation loss failed to decrease by at least 1 × 10^−6^ for at least 10 epochs. The model checkpoint with the best validation loss was selected for all subsequent evaluation and inference.

### Proofread data

Proofreading entails a review of SLEAP predictions to ensure accurate extraction of root trait data on each dataset (Table 3). Given that the user will simultaneously observe the plant within the cylinder, a quality control (QC) step has been integrated into this process.

For uniform QC both between and within individual annotators, a set of predetermined QC codes has been developed for each proofread dataset. These codes pertain to specific rules governing plant exclusion. The primary criterion for exclusion is contamination. While the cylinder media is nutrient-rich, and microbial and fungal species might persist after sterilization, slight contamination is permissible. Nevertheless, if the contamination impedes clear observation of the RSA, the plant will be excluded (coded as “cont”). Additional exclusion criteria include the plant being fully submerged (coded as “sub”), oriented incorrectly (coded as “ori”), poor growth (coded as “pg”), and plant death (coded as “dead”). Examples of excluded samples per dataset are provided with the labeling rules in the Supplementary Materials.

Once a plant passes the QC stage, the user proceeds to inspect the predictions in the SLEAP GUI. Common corrections involve the detection of non-root segments in the image, such as media or cylinder deformities, or oversight of prominent RSA features. Upon making the necessary corrections, the user saves them, and the refined root trait data are then extracted from this proofread dataset.

The proofreading stage also provides users with the opportunity to discern recurring error patterns produced by the model. Identifying such consistent errors can be instrumental in model refinement. Any such instances are carefully labeled according to established protocols and subsequently incorporated into the training dataset. To foster collaboration and information exchange among users, a cloud-hosted spreadsheet was used. This spreadsheet is organized with columns for the user’s initials, QC outcomes (binary: accepted or rejected), specific QC codes, and the frames earmarked for inclusion in the training set, with each row corresponding to an individual plant.

### Trait extraction

**Fig. 4.**
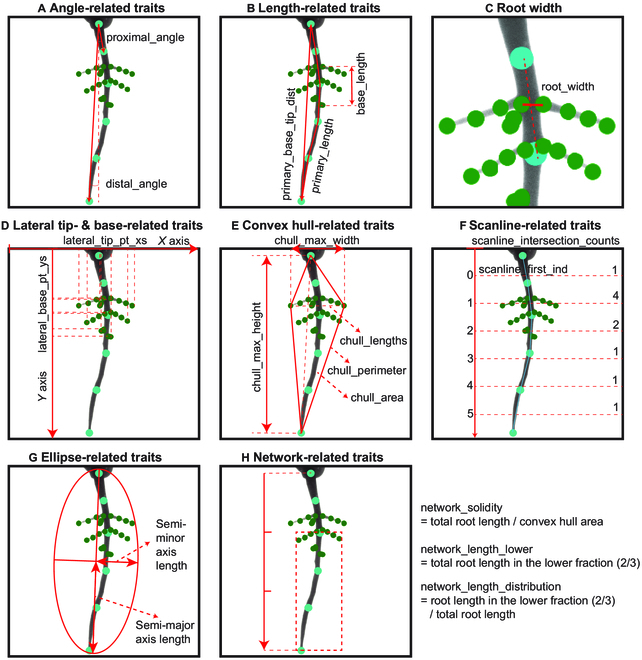
(A to H) Schematics of root trait computation methods from landmark data. The landmarks in cyan are primary root predicted landmarks, while those in green are lateral root landmarks. Detailed descriptions of each trait and category are provided in Table 4.

The code repository has a modular design, allowing flexibility and adaptability, making it suitable for a wide range of root analysis tasks (https://github.com/talmolab/sleap-roots). Its functions for calculating specific root traits (Fig. 4) from root detection with SLEAP are designed to be utilized independently or as a part of our engineered pipelines, facilitating their application in imaging systems beyond the RADICYL cylinder system. *sleap-roots* (v0.0.5) can be installed with all of its dependencies using a pip package (https://pypi.org/project/sleap-roots/). We also make the code used in this paper available as a zip file for reproducibility in the Supplementary Materials. We summarize the modules of the *sleap-roots* package below. For detailed information on the implementation of trait calculations, please reference the source code.

**Series extraction:**

In the *sleap_roots.series* module, the *Series* class is central to managing and analyzing data related to a single image series of root networks. This class is constructed using the *attrs* package (v23.1.0) and is designed to handle data and predictions associated with an image series. The class attributes include paths to the HDF5-formatted image series (*h5_path*), primary root predictions (*primary_labels*), lateral root predictions (*lateral_labels*), and a video representation of the image series (*video*). These attributes are primarily managed using the *sleap-io* (v0.0.11) package.

The load class method facilitates the loading of predictions for a given series. It constructs paths to the prediction files based on provided root names and uses the *sio.load_slp* function to load these predictions. Additionally, the video representation of the image series is loaded using *sio.Video.from_filename*.

The class also provides utility methods and properties, such as *series_name*, which extracts the name of the series from the HDF5 filename using the *pathlib* library. The *__len__* method returns the number of images in the series, while the *__getitem__* and *__iter__* methods enable indexing and iteration over the series, respectively.

The *get_frame* method returns labeled frames for both primary and lateral predictions for a specified frame index. This is achieved by finding the corresponding labeled frames using the find method from the *sio.Labels* class.

Visualization capabilities are provided by the *Series.plot* method, which overlays predictions on the image. This method utilizes the *matplotlib* (v3.8.0) and *seaborn* (v0.13.0) libraries for plotting. The *get_primary_points* and *get_lateral_points* methods retrieve the primary and lateral root points, respectively, for a given frame index. These methods return the root points as numpy arrays, with shapes indicating the number of instances and nodes.

**Angle-related traits:**

The *sleap_roots.angle* module is designed to compute angles between vectors that represent root structures and the gravity vector (Fig. 4A and Table 4A). To represent a root, a vector is constructed using the base node and either the distal or proximal node. The distal node is identified as the first non-NaN node in the initial half of the root, while the proximal node is the last non-NaN node in the latter half of the root. The computation of the angle between these vectors is achieved using the *numpy.arctan2* function from the *numpy* library (v1.26.0).

**Table 4.** Pose-derived trait description (associated with Fig. 4)

Trait name	Units	Trait type	Description
A. Angle-related traits	primary_angle_proximal	Degree	Scalar	Primary root angle calculated by base node and proximal node
primary_angle_distal	Degree	Scalar	Primary root angle calculated by base node and distal node
lateral_angles_proximal	Degree	Non-scalar	Lateral root angle calculated by base node and proximal node—each lateral root will have one value
lateral_angles_distal	Degree	Non-scalar	Lateral root angle calculated by base node and distal node—each lateral root will have one value
main_angles_proximal	Degree	Non-scalar	Main root angle calculated by base node and proximal node—each main root will have one value
main_angles_distal	Degree	Non-scalar	Main root angle calculated by base node and distal node—each main root will have one value
B. Length-related traits	primary_length	px	Scalar	Length of primary root, computed as the sum of the lengths of the segments that form the primary root
lateral_lengths	px	Non-scalar	Lengths of each lateral root, computed as the sum of the lengths of the segments that form each lateral root
main_lengths	px	Non-scalar	Lengths of each main root, computed as the sum of the lengths of the segments that form each main root
primary_base_tip_dist	px	Scalar	Euclidean distance (straight line distance) from the base node to tip node of the primary root
main_base_tip_dists	px	Non-scalar	Euclidean distances from the base nodes to the tip nodes of each main root
primary_tip_pt_y	px	Scalar	*Y* coordinate of tip of primary root
curve_index	-	Scalar	Primary root curvature index, calculated by *curve_index = (primary_length* − *primary_base_tip_dist)*/*primary_length*
main_curve_indices	-	Non-scalar	Curve index of each main root
C. Root width	root_widths	px	Non-scalar	Primary root width based on paired lateral root bases
D. Tip- and base-related traits	lateral_base_pt_xs	px	Non-scalar	*X* coordinate of each lateral root base point
lateral_base_pt_ys	px	Non-scalar	*Y* coordinate of each lateral root base point
lateral_tip_pt_xs	px	Non-scalar	*X* coordinate of each lateral root tip point
lateral_tip_pt_ys	px	Non-scalar	*Y* coordinate of each lateral root tip point
main_tip_pt_xs	px	Non-scalar	*X* coordinate of each main root tip point
main_tip_pt_ys	px	Non-scalar	*Y* coordinate of each main root tip point
base_length	px	Scalar	Difference in *y* coordinates between first and last lateral bases
base_length_ratio	-	Scalar	base_length/primary_length
base_median_ratio	-	Scalar	Ratio of median value in all lateral base points to tip of primary root in y axis, calculated by *base_median_ratio = numpy.nanmedian(lateral_base_ys)*/*primary_tip_pt_y*
base_ct_density	1/px	Scalar	Ratio of number of base points over primary root length
lateral_count	Roots	Scalar	Number of lateral roots
main_count	Roots	Scalar	Number of main roots
E. Convex hull-related traits	chull_line_lengths	px	Non-scalar	Lengths of lines connecting any two vertices on the convex hull
chull_perimeter	px	Scalar	Perimeter of convex hull
chull_area	px^2^	Scalar	Area of convex hull
chull_max_width	px	Scalar	Maximum width—difference in *x* coordinates—of convex hull (taken between vertices)
chull_max_height	px	Scalar	Maximum height—difference in *y* coordinates—of convex hull (taken between vertices)
F. Scanline-related traits	scanline_intersection_counts	-	Non-scalar	Number of intersections with roots at each scanline
scanline_first_ind	-	Scalar	Index of the first scanline that intersects with roots
scanline_last_ind	-	Scalar	Index of the last scanline that intersects with roots
G. Ellipse-related traits	ellipse_a	px	Scalar	Semi-major axis length of the fitted ellipse of the root network
ellipse_b	px	Scalar	Semi-minor axis length of the fitted ellipse of the root network
ellipse_ratio	-	Scalar	Ratio of the major to minor lengths
H. Network-related traits	network_length	px	Scalar	Total root length
network_width_depth_ratio	-	Scalar	Width-to-depth ratio of bounding box of root network
network_solidity	1/px	Scalar	Total root length divided by the convex hull area
network_length_lower	px	Scalar	Total root length in the lower fraction (2/3) of the network bounding box
network_length_distribution	-	Scalar	Total root length in the lower fraction (2/3) of the network bound box over total root length

**Length-related traits:**

In the *sleap_roots.lengths* module, root lengths are computed by summing the segment lengths (Euclidean distance) between nodes for each individual root (Fig. 4B and Table 4B). This calculation utilizes the *numpy.diff* and *numpy.linalg.norm* functions from the *numpy* library (version 1.26.0). The total length for each root is then aggregated using *numpy.nansum*.

**Tip- and base-related traits:**

In the *sleap_roots.tip* module, tips of roots are identified as the terminal nodes using *numpy* array operations (v1.26.0). Conversely, bases are recognized as the initial nodes of each root. Utilizing these landmarks, various traits can be derived, including the density of base points on the primary root, denoted as *base_ct_density* (Fig. 4D and Table 4D).

For dicot plants, the bases of lateral roots are employed to approximate root widths (Fig. 4C and Table 4C). Initially, lateral root bases on both the right and left flanks of the primary root are pinpointed. Subsequently, a *LineString* object representing the primary root is constructed using the *LineString* function from *shapely.geometry* (*shapely* v2.0.1). To locate points on the primary root proximate to the bases of the lateral roots, we employ *nearest_points* from *shapely.ops* and *Point* from *shapely.geometry*. Each point’s normalized projection, relative to the length of the primary root’s *LineString*, is then determined for every base on both the left and right sides. A cost matrix is formulated based on the discrepancies in projections between the bases on the two sides. Utilizing the Hungarian matching algorithm, an optimal pairing between the left and right bases is established, aiming to minimize the total difference in projections. This is achieved using *linear_sum_assignment* from *scipy.optimize* (*scipy* v1.11.3). After the application of a user-defined tolerance for projection differences between base pairs (default set at 0.02) and the exclusion of pairs not intersecting the primary root line, the distances between the matched base pairs are computed using *numpy.linalg.norm*.

**Convex hull-related traits:**

Within the *sleap_roots.convhull* module, the convex hull is determined using the ConvexHull function from *scipy.spatial* (scipy v1.11.3). Traits derived from the convex hull include the convex hull perimeter, area, width, height, and distances between vertices (Fig. 4E and Table 4E).

**Scanline-related traits:**

Within the *sleap_roots.scanline* module, functions employ horizontal “scanlines” to quantify the number of roots based on their intersections with these lines. This procedure is executed using *numpy* array operations (v1.26.0) on the list of points that delineate the root network (Fig. 4F and Table 4F).

**Ellipse-related traits:**

Within the *sleap_roots.ellipse* module, the *fit_ellipse* function is designed to approximate an ellipse to the points characterizing the root network. This function yields the semi-major and semi-minor axes of the fitted ellipse, in addition to the ratio of the major to minor lengths (Fig. 4G and Table 4G). This is achieved using the *EllipseModel* from *skimage.measure* (*scikit-image* v0.22.0).

**Network-related traits:**

In the *sleap_roots.networklength* module, a suite of functions is dedicated to the analysis of the root network. The *get_network_distribution* function leverages the *Polygon* class from the *shapely* library (v2.0.1) to transform the lower fraction of the root network’s bounding box into a polygon. For each root, *LineString*s are formed, and the intersection and length properties of shapely’s *LineString* are utilized to compute the length of roots that intersect with this polygon. The total *network_length* is ascertained by aggregating the lengths of all roots in the network using *numpy.nansum*, with individual root lengths being pre-calculated via the *get_root_lengths* function. The remainder of the computations in the *sleap_roots.networklength* module predominantly employs *numpy* array operations (Fig. 4H and Table 4H).

**Trait graphs:**

In the *sleap_roots.trait_pipelines* module, trait maps are implemented for different types of plants. The module employs a directed graph to represent the hierarchical dependencies between root traits. The graph starts at the points from the SLEAP predictions and ends with root traits per plant. Classes are defined for each unique trait map, which determine the initial points fed into the graph and the connections between nodes. The graph representation offers flexibility, making it easy to adapt the pipeline for different input predictions and relationships between traits. This adaptability ensures that the pipeline remains versatile and can cater to the specific needs of various types of plants with different trait relationships.

The graph was built using *networkx.DiGraph* from the *networkx* package (v3.1). To ensure that traits are computed in the correct sequence, respecting their dependencies, the computation order was defined using *networkx.topological_sort*. By leveraging topological ordering, the module can systematically compute each trait only after its dependent traits have been calculated, ensuring efficiency in the trait extraction process. The pipelines made are *DicotPipeline* and *YoungerMonocotPipeline*.

#### DicotPipeline

An advanced root trait extraction pipeline was designed to process SLEAP predictions and subsequently generate root traits for dicot plants in CSV format. This pipeline is tailored for plants with primary and lateral root predictions.

From each frame, 35 distinct traits were derived, culminating in a total of 1,035 traits for each plant. A visual representation of these 35 traits was constructed using a mermaid graph (Fig. S1), originating from the SLEAP landmarks, where individual nodes symbolize traits and arrows between nodes show the actions of the functions in the sleap-roots code repository as defined in the trait map for the dicot pipeline.

Of the 35 root morphological traits computed, 25 are scalar traits and 10 are non-scalar traits. For these 10 non-scalar traits, a suite of nine summary statistics—maximum, minimum, mean, median, SD, and percentiles at 5th, 25th, 75th, and 95th—was applied. This led to the derivation of 90 summarized non-scalar traits per frame. By applying summary statistics to each frame trait, a comprehensive set of 1,035 summarized traits per plant was achieved.

#### YoungerMonocotPipeline

Similarly, a pipeline tailored to the younger monocot predictions, consisting of primary and crown roots—referred to as “main” root traits for brevity—was constructed beginning at the predictions and ending at traits in CSV format. For each frame, 30 distinct traits are calculated. The mermaid graph depicting the trait for younger monocots is shown in Fig. S2. In this pipeline, there are 21 scalar and 8 non-scalar traits per frame. The summary statistics—maximum, minimum, mean, median, SD, and percentiles at 5th, 25th, 75th, and 95th—were applied to the non-scalar traits per frame and again over the 72 images in the series, resulting in 918 traits per plant.

### Manually measured traits using Fiji

Given the labor-intensive nature and time demands of manual trait measurements, we opted to focus our error evaluation using manual trait measurements on a select number of traits for rice, a monocot, and soybean, a dicot. Traits were measured on one representative frame per plant. These traits were chosen because they are representative descriptors of the RSA and are more straightforward to measure manually, minimizing potential measurement errors. We used Fiji’s multi-point tool for point measurement and the segmented line tool for root lengths [47]. For rice, we evaluated the *y* coordinate of the deepest root tip, where roots could be either primary or crown roots, and the primary root length, which correspond to the traits in Table 4 of *main_tip_pt_ys_max_median* and *primary_length_median* from the younger monocot pipeline. For soybean, we assessed the *y* coordinates of the highest lateral root base and the deepest lateral root tip, corresponding to the traits of *lateral_base_ys_min_median* and *lateral_tip_pt_ys_max_median* from the dicot pipeline. We chose to evaluate the median of each trait over the 72 frames since it is a robust estimator of the trait and a fair comparison with the representative trait chosen by the annotator.

### Comparison with RootPainter

This study aimed to assess our computer vision approach for plant phenotyping in comparison with the established approach of using RootPainter for segmentation and RhizoVision Explorer for segmentation-based trait extraction [19,24,48]. Both RootPainter and SLEAP were used to train minimal models, and root traits derived from each method were compared with the ground truth. Time metrics for every phase were documented. The goal was to label sufficiently in both software to yield acceptable predictions—sufficiently discerning between root and non-root regions—and compare the time it took to create a “minimally accurate model” in each. We used three DAG rice images, labeling both crown and primary roots, to train a model on each platform.

Our workstation ran on Windows 10 Pro, powered by an Intel Core i7-9700K CPU (3.60 GHz, 8 cores, 8 logical processors), paired with an NVIDIA GeForce RTX 2070 Super boasting 8 GB of VRAM, operating with driver version 531.79 and CUDA version 12.1.

For the SLEAP benchmark, we made a project for the primary and crown roots of three DAG rice samples. From a randomly chosen subset of 10 plants, 2 frames per plant were labeled (20 frames in total), noting the labeling duration for each. Adhering to the labeling rules defined for the “Rice crown roots (3 DAG)” model, the 1st and 30th frames from each plant were labeled. Following this, the model underwent training based on the configuration detailed elsewhere in this article. Testing was conducted on a distinct set of 75 user-labeled frames. The resultant predictions met acceptable standards. The derived root traits from SLEAP’s model and the labeled frames were compared, and errors were assessed as the absolute difference between the two sets.

For the RootPainter benchmark, the aforementioned 10 rice plants chosen for SLEAP were reintroduced to RootPainter (version 0.2.25; [24]). The images were resized to half, matching input scaling used in SLEAP, and cropped to isolate the cylinders, resulting in a final 512 px × 512 px dimension. One tile per image was used. The initial labeling spanned 38 min 46 s. The instructions from the RootPainter GitHub page [49] guided the labeling process, maintaining a consistent pace. Training was initiated after completing two labeled images. Upon evaluating the predictions after six labels, they were still essentially random. Extended training was permitted until corrective predictions seemed viable. Notably, enhanced accuracy required more extensive labeling, and corrective annotations markedly refined the model. In total, 14 frames were labeled, achieving desirable predictions. The best model was used to segment the same 75 frames tested with SLEAP (these frames are also distinct from the training set used in RootPainter). The derived segmentations were directed to RhizoVision Explorer for root trait extraction, employing specific settings: whole-root mode, retention of the largest component, image threshold set at 200, and root pruning active at a threshold of 5. Root traits derived from RhizoVision Explorer were benchmarked against the ground truth for error computation.

### Statistical analysis

**Fig. 5.**
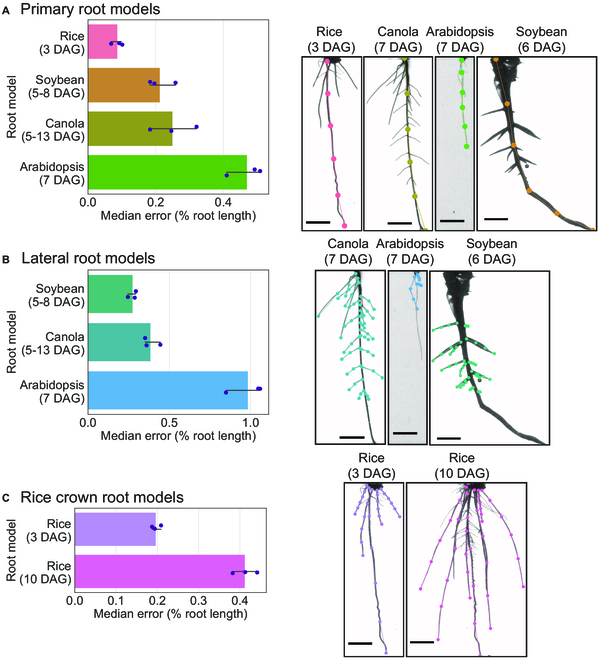
SLEAP models accurately located root landmarks in four species and three classes of roots. (A) Primary root, (B) lateral root, and (C) crown root accuracies and predictions are displayed. The accuracy is calculated as the median localization error for a randomly selected held-out test set normalized by the average root length for that dataset. The bar graph presents the models ordered by mean accuracy, with error bars showing the 95% confidence interval (*n* = 3 random splits). Scale bar, 1 cm.

We use the localization error as one evaluation metric for the accuracy of the models (Figs. 5 and 8). The localization error is calculated as the Euclidean distance between the predicted landmark and the ground truth human annotated landmark. It reflects the performance with which our approach can correctly predict the locations of morphological features in 2D images.

For each model, we created three randomly selected test sets of plants we had ground-truth labels for. The test set is approximately 10% of the total labeled data for each model (Tables S1 and S6). We trained a model with each training set and evaluated the model on the plant-wise test set hidden from the model during the training.

**Fig. 6.**
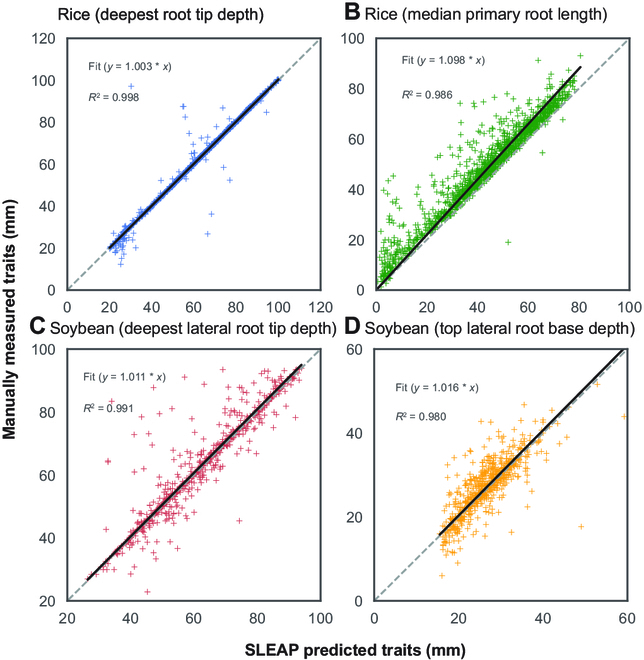
Scatter plots of traits predicted using SLEAP and extracted using the *sleap-roots* package versus manual measurements in Fiji. The gray dashed lines represent the 1:1 lines (*y* = *x*), while the black solid lines depict the regression lines, with the associated regression functions and *R*^2^ values displayed in each panel. The *younger monocot* pipeline is tested using the (A) deepest root tip depth (*main_tip_ys_max_median*) and the (B) primary root length (*primary_length_median*) (*n* = 1,301 plants). The *dicot pipeline* is tested using the (C) deepest lateral root tip depth (*lateral_tip_pt_ys_max_median*) and (D) the highest lateral root base depth (*lateral_base_pt_ys_min_median*) (*n* = 600 plants).

We assessed the accuracy of SLEAP-predicted root traits by regressing them against corresponding manually measured values obtained via Fiji (Fig. 6). The coefficients of determination (*R*^2^) and the coefficient of regression lines were calculated. Two rice traits (deepest root tip depth and primary root length) and two soybean traits (deepest lateral root tip depth and highest lateral root base depth) were used to evaluate the SLEAP-based traits error.

**Fig. 7.**
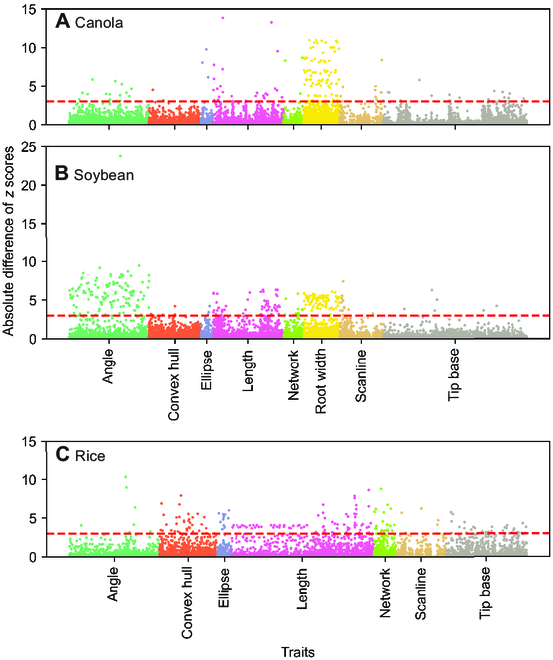
Comparison of proofread and non-proofread traits for each crop. Manhattan plots of the normalized difference between traits derived from proofread and non-proofread data for (A) canola (7 DAG), (B) soybean (6 DAG), and (C) rice (3 DAG). The *x* axis of each figure indicates unique traits grouped by category. The *y* axis of each figure represents the absolute difference between proofread and non-proofread trait values (normalized by the mean and SD of the proofread traits). Points correspond to individual trait values for individual plants. The red dashed line represents three SDs of the proofread trait distribution. *Y* axes are truncated for clarity, and additional distribution summaries can be found in Tables S3 to S5.

Proofreading of SLEAP predictions on a data subset was performed to assess the discrepancy between user-corrected outputs and initial machine predictions, and to observe the traits most impacted by the discrepancy. The absolute difference of *z* scores were used to compare each trait of each plant before and after proofreading (Fig. 7). The *z* score for each trait and plant was calculated based on the formula *z* = (μ − *x*)/σ, where *x* is the trait value, μ is the average trait value for proofread plants, and σ is the SD of trait value for proofread plants.

Furthermore, to complement the *z*-score analysis and provide a quantitative measure of the linear relationship between proofread and non-proofread traits, we calculated the Pearson correlation coefficient (Tables S3 to S5). This statistical measure evaluates the degree to which two datasets are linearly related, offering insights into the consistency between the machine-generated predictions and the subsequent user corrections. A high Pearson correlation indicates a strong agreement between the proofread and non-proofread data, suggesting that the initial machine predictions were relatively accurate, requiring minimal corrections or that prediction inaccuracies did not impact trait calculations. Conversely, a lower correlation would highlight trait discrepancies.

The choice of using *z*-score normalization, coupled with Pearson correlation, serves a dual purpose. First, it standardizes the trait data, making it possible to objectively assess and compare the magnitude of deviations between corrected and original predictions across various traits and plants. Second, it quantifies the alignment between the datasets, providing a clear metric to gauge the initial predictive accuracy of the models. This comprehensive approach ensures a robust evaluation of the differences between proofread and non-proofread traits, guiding further refinements in both the predictive models and the proofreading methodologies.

SLEAP-based plant traits were used to do plant accession classification using random forest classifiers (*sklearn.ensemble.RandomForestClassifier*) (Fig. 10). We selected 70 plants from 10 accessions with more than six replications per accession. The plant traits were derived based on proofread SLEAP predictions. Fivefold cross-validation overall accuracy (*accuracy_score*) was used to evaluate random forest performance. Four random forest classifier parameters were fine-tuned with a randomized search algorithm. The parameters were number of trees, maximum depth of the tree, minimum samples per split, and minimum samples per leaf. The best model was selected based on the highest accuracy. The parameter settings of the best classifier are *max_depth* of 10, *min_samples_leaf* of 1, *min_samples_split* of 4, and *n_estimators* of 712.

The SHAP algorithm was used to evaluate the accession classification trait importance [36]. The input traits were the same as the accession classification dataset, while the target is the plant accession as well. SHAP determined the importance value for each trait. A higher SHAP value means that this trait plays a more important role in the classification of accessions. A precision–recall curve was conducted to show the trade-off between precision and recall. The average precision value was used based on the precision values of 10 accessions with macro metrics. A higher area under the curve signifies a higher precision and recall value. Based on SHAP, three traits were selected with varying amounts of importance (most importance, average importance, least importance). The Kruskal–Wallis *H*-test was conducted for each trait to test whether the median value of all genotypes is equal or not. We opted for the Kruskal–Wallis one-way analysis of variance, a nonparametric test, due to its suitability for our dataset characterized by high variability within genotypes, non-normally distributed phenotypic trait values per genotype, and unequal variances across different genotypes. This approach is more appropriate for our data’s nature, where traditional parametric assumptions do not hold [50].

To confirm the reliability of root traits derived from sleap-roots, we conducted exploratory data analysis and visualized phenotypic trait spaces (Fig. 11). Our *DicotPipeline* aggregated 1,035 root traits across 2,082 soybean samples. Only samples without any missing (NaN) values across all traits were retained, resulting in a dataset of 2,009 samples. We standardized the data using *z*-score normalization followed by PCA employing the *StandardScaler* and *PCA* modules from *scikit-learn* (v1.0), respectively. To robustly estimate the covariance of the dataset, a minimum covariance determinant (MCD) [51] estimator was fitted using scikit-learn’s *MinCovDet* on 75% of the data variability represented by the first eight principal components. For dataset quality assurance, we adopted the Mahalanobis distance as a metric to flag potential outliers, leading to manual inspection of all samples with a distance value of 10 or above. This QC step resulted in the retention of 1,877 samples. Subsequent PCA was performed after outlier exclusion to identify the traits with the highest variance explanation, which were then employed to annotate UMAP visualizations of the phenotypic trait space. The UMAP embeddings were generated using default settings on the *z*-score normalized dataset.

## Results

### Pose estimation robustly localizes root system landmarks across species

The principal output of pose estimation models is the locations and groupings of morphological landmarks. In order to evaluate how well our models perform at identifying roots and locating their landmarks on 2D images, we measured the localization error (how far predicted landmark coordinates are from the ground truth) in held-out test sets for each model and dataset (randomly split and repeated in triplicate). We find that all of our models have median localization error of less than 1% of the root length (Fig. 5 and Fig. S2). The most accurate model relative to its root length was the rice primary root 3 DAG model at 0.087% root length (0.540 mm) followed by the rice crown root 3 DAG model at 0.197% root length (0.662 mm). The model with the highest error relative to its root length was the Arabidopsis lateral 7 DAG model at 0.984% root length (0.843 mm) followed by the Arabidopsis primary 7 DAG model at 0.470% root length (1.537 mm) and the rice crown 10 DAG model at 0.412% root length (2.281 mm). These results indicate that pose estimation models are highly accurate at locating biologically meaningful morphological landmarks from 2D root images.

### Phenotypic traits are accurately extracted from root system landmarks

Localization accuracy measures how well morphological landmarks are recovered; however, this may not directly translate to equivalent performance on downstream trait extraction. To estimate how well our approach can recover phenotypic traits, we compared the predicted traits derived automatically from model-predicted landmarks with manually annotated ground truth across a large number of plants (*n* = 1,301 rice and 600 soybean plants). Since manual annotation of individual traits is highly laborious, we restrict this analysis to a comparison of a subset of the whole suite of traits that our pipeline produces. For two rice traits from the younger monocot pipeline (Fig. 6A and B), the *R*^2^ values were 0.998 and 0.986, while the coefficients of regression lines were 1.003 and 1.098 for the deepest root tip depth (*main_tip_ys_max_median*) and median primary root length (*primary_length_median*), respectively. For two soybean traits from the dicot pipeline (Fig. 6C and D), the *R*^2^ values were 0.991 and 0.980, while the coefficients of regression lines were 1.011 and 1.016 for the deepest lateral root tip depth (*lateral_tip_pt_ys_max_median*) and highest lateral root base depth (*lateral_base_pt_ys_min_median*), respectively. In both rice and soybean, the traits derived from the younger monocot and dicot pipelines demonstrated a high degree of correlation with manually measured traits, as indicated by the high *R*^2^ values and coefficients of regression lines close to 1, underscoring the reliability and accuracy of these pipelines in phenotypic trait assessment. In comparing root length measurements, we observed that lengths obtained using *sleap-roots* are generally shorter than manual measurements. This discrepancy likely arises from the fixed number of nodes in *sleap-roots*, in contrast to Fiji’s segmented line tool, which allows users to select an arbitrary number of nodes to closely follow root curvature. Notably, this difference in measurements is more pronounced in roots with greater curvature. Despite these discrepancies, *sleap-roots* demonstrated a strong correlation with manual measurements across all samples, indicating its reliability in approximating root lengths and tip depths.

As it was not feasible to extract all traits for all plants via manual annotation, we next compared the accuracy of traits derived from automatically predicted landmarks against manually corrected landmarks for proofread datasets (Fig. 7). Manual landmark proofreading is much faster than manual trait annotation, but trait results may differ since proofreading corrects predictions, while manual measurements in Fiji are not informed by predictions (and conducted in a different software). We estimate trait computation accuracy as the absolute difference of *z* scores between proofread and non-proofread predictions for each trait and plant. The *z* scores were calculated using the estimators of the distributions of the proofread traits so that the difference in *z* scores (Δz) can be interpreted as the trait error in units of true SD intrinsic to the trait. Additionally, we calculate the correlation and 95th percentile (Δz95) between proofread- and non-proofread-derived traits for each dataset to aid in interpretability of the standardized differences (Tables S3 to S5).

In our dicot datasets, we are able to predict up to 1,035 traits using the primary and lateral root landmarks (if sufficient root landmarks are detected). For canola (Fig. 7A and Table S3), we find that 96.2% of trait prediction errors (Δz) are within 1 SD of the proofread ground truth (*n* = 110,745 predicted trait values across 107 plants). For soybean (Fig. 7B and Table S4), 96.3% of Δz are within 1 SD (*n* = 72,750 predicted trait values across 70 plants). For monocot datasets, our pipelines predict up to 918 traits due to the absence of lateral roots. For rice (Fig. 7C and Table S5), we find that 99.5% of Δz are within 1 SD of the proofread ground truth (*n* = 247,860 predicted trait values across 270 plants).

These results show that manual landmark proofreading can improve trait estimation accuracy, increasing the proportion of trait values that are within 1 SD of ground truth by 0.5% to 3.8%. This indicates that the trait values predicted by our approach are highly accurate across trait categories and species even in the fully automated setting.

### Landmark-based methods are faster and more efficient than segmentation-based approaches

A key advantage of pose estimation is that it requires less laborious annotation than segmentation since only the landmark points need to be annotated by the user. In SLEAP, this is further sped up by human-in-the-loop annotation, in which models are iteratively trained and used to generate predictions, and those predictions are imported to the annotation GUI for proofreading.

**Fig. 8.**
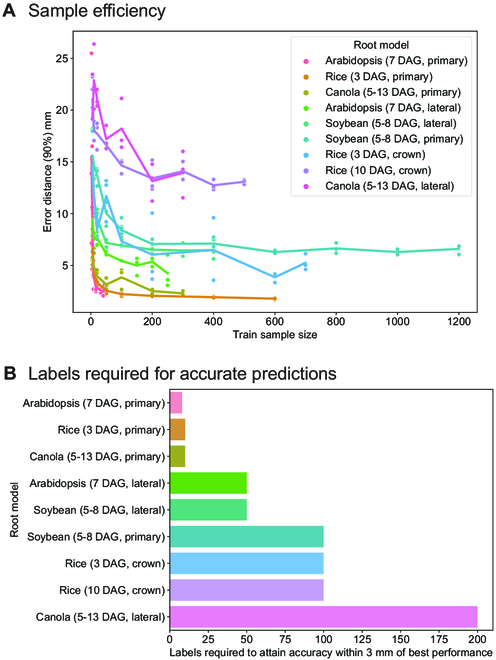
Efficient models require fewer labels for accurate predictions and facilitate diverse training sets. (A) Models with the number of labeled frames given by the train sample size were trained for each root model. The *y* axis shows the localization error at the 90 percentile so that error-prone examples are included in this error estimation (we use the high end of the error distribution). The line plot connects the average error distance at each sample size (*n* = 3). The sample efficiency curves plateau as the increase in training sample size does not further reduce the localization error. (B) We can estimate the number of labeled frames needed for accurate predictions by asserting a threshold of 3 mm as the change in localization error with a change in the number of labeled frames in the training set. For most models, we are within 3 mm of the best model within 100 labeled frames.

While annotation speed is a key factor for generating reliable models, it is not clear how many annotations are necessary to achieve high accuracy. To determine this, we conducted a series of sample efficiency experiments, in which subsamples of each dataset were used to train SLEAP models (Fig. 8). In agreement with previous work using SLEAP in plants [30], we observe diminishing improvements in accuracy beyond tens to hundreds of labels (Fig. 8A). Relative to the peak accuracy, most datasets require <100 labeled images, with the exception being canola lateral root models that achieve peak accuracy at 200 labels (Fig. 8B). Canola is the exception, due to the large amount of diversity of the training set; these plants have the widest range in age, from 5 to 13 DAG, and were randomly selected from a very large diversity screen spanning over many months. These results indicate that pose estimation models can be trained to predict plant root landmarks at high accuracy with relatively little annotated data, making our approach practical and time efficient to use.

**Fig. 9.**
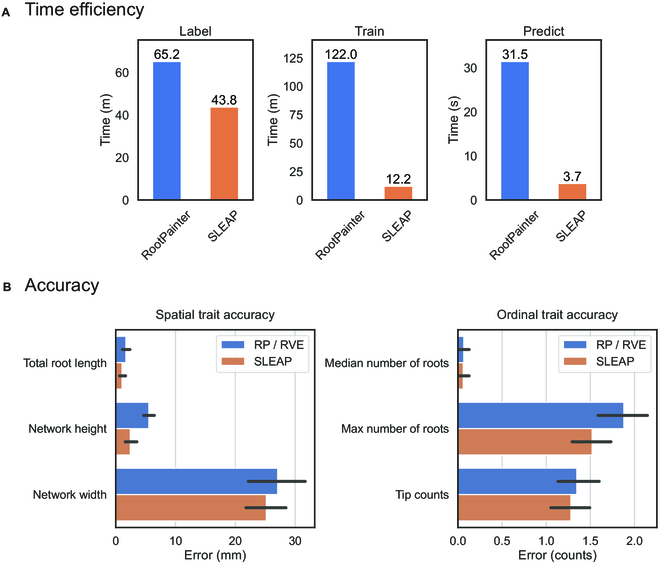
Root traits are extracted in a fraction of the time with the same or better accuracy as segmentation-based methods. (A) Minimal models for accurate root predictions are labeled and trained in both RootPainter and SLEAP, and the time is recorded. Seventy-five root images are predicted using these trained models. (B) The accuracy of the predictions from this minimally trained model is evaluated for the common set of traits between RhizoVision Explorer and *sleap-roots*. The height of the bar graphs is the mean error, with error bars showing the 95% confidence interval (*n* = 75). RP, RootPainter; RVE, RhizoVision Explorer.

Next, we compare the speed and accuracy of our approach to a segmentation-based approach. RootPainter [24] is a commonly used, user-friendly segmentation tool that employs deep learning and human-in-the-loop annotation workflow, which continuously trains and generates predictions during the annotation process. We find that we can train a model in SLEAP using relatively few labeled frames and the time to label, train, and predict using pose estimation is lower than RootPainter. We found a 32.8% decrease in labeling time when using SLEAP to annotate landmarks (Fig. 9A). Training and inference time was decreased in SLEAP: There was a 90.0% decrease in training time and 88.1% decrease in inference time (Fig. 9A). Root trait extraction times using the younger monocot *sleap-roots* pipeline with all 918 traits took an average of 0.44 s per plant (*n* = 1,153). The dicot pipeline with all 1,035 traits took an average of 0.70 s per plant (*n* = 2,082). We then employed RhizoVision Explorer [19] to extract root traits from the RootPainter-predicted segmentation masks. When we compare the root traits extracted from the two pipelines, we find that SLEAP has the same or better accuracy (Fig. 9B).

### Pose estimation-based traits can be used for downstream phenotypic analyses

As a proof of principle that our pipeline can be used in common downstream analyses of plant RSA phenotypes, we used the traits we extracted in both supervised (genotype classification) and unsupervised (UMAP visualization) tasks.

**Fig. 10.**
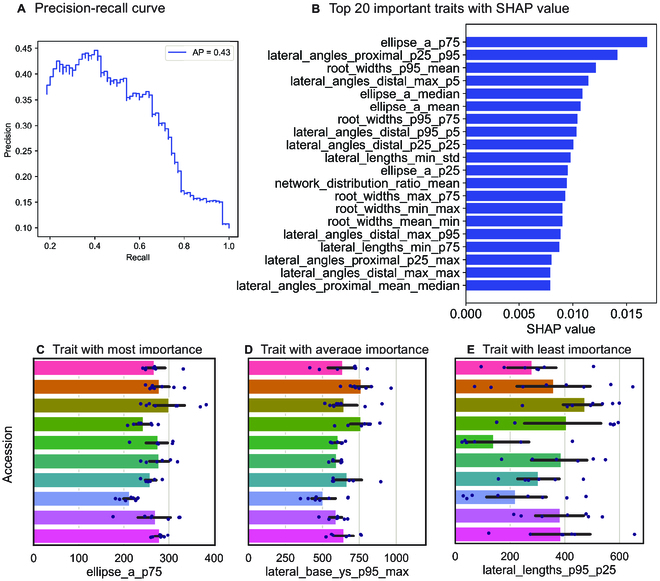
Genotype prediction using phenotypic traits. (A) Classification performance using precision–recall curve of accession classification of 70 diverse soybean plants. AP stands for average precision value. (B) Top 20 most important traits for accession classification using fine-tuned random forest best parameters. (C to E) Three traits showing varying amounts of importance in genotype differentiation of samples. The *x* axis is the trait value, while the *y* axis represents the accession. The *P* values of the Kruskal–Wallis *H*-tests are 0.00182, 0.00316, and 0.02970 for the most, average, and least important traits, respectively.

For classification, we used traits from our pipeline to predict the genotype of soybean plants. Seventy plants were sampled from 10 accessions to perform supervised classification. Each accession had at least six replicates. The best random forest classifier achieved an overall accuracy of 0.400 (chance: 0.1) using fivefold cross-validation after the parameter fine-tuning. A precision–recall curve was generated based on the accession classification results (Fig. 10A) with an average precision of 0.43. We then used SHAP [36] to evaluate the importance of each trait with the best random forest classifier. The top 20 most important traits are shown in Fig. 10B, including the lateral angle-, root width-, and ellipse-based traits. The least important traits were scanline count-, lateral base-, and lateral tip-based traits. Three typical traits with the most, average, and least importance for accession classification are visualized in Fig. 10C to E. The *P* values of the Kruskal–Wallis *H*-tests are 0.00182, 0.00316, and 0.02970 for the most, average, and least important traits, respectively. These analyses demonstrate that traits derived from our pipeline can be used to train genotype classifiers with sufficient accuracy and explainable predictions.

**Fig. 11.**
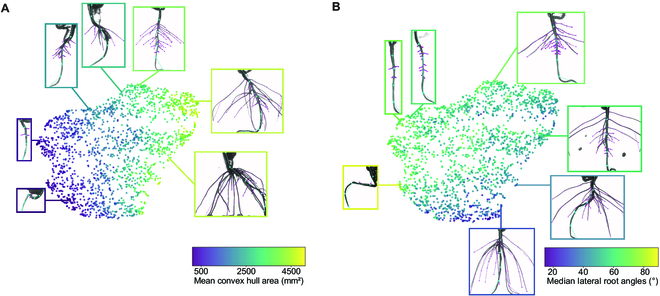
Visualization of phenotypic space through large-scale root trait mapping (*n* = 1,877). (A) The UMAP, color-coded according to the mean convex hull area, illustrates the dominant role this trait plays in shaping the trait space. (B) A similar color-coding strategy is used to highlight the median lateral root angle’s contribution to the global trait structure. These examples, each uniquely colored based on their corresponding trait, offer a comprehensive view of the phenotypic diversity observed in the screen.

Next, we used the predicted traits for soybean across 1,877 plants to map out the phenotypic space of these plants, a common task in high-throughput screening (Fig. 11). Upon conducting a PCA, we observed that the trait representing the mean convex hull area (*chull_area_mean*) emerged with one of the highest coefficient magnitudes in the linear transformation pertaining to the first principal component. This indicates a predominant influence of this trait on the variance captured by the first principal component. Similarly, the median distal lateral root angle (*lateral_angles_distal_median_median*) demonstrated a substantial coefficient magnitude in relation to the second principal component, suggesting its considerable impact on the variance described by this component. These findings illustrate that these two traits play a major role in their respective principal component axes, providing an orthogonal representation of the trait space within the first two dimensions, which accounted for 50.62% of the total explained variance. When these traits were used to inform the coloring of the UMAP, the resulting visualization offered an interpretable map of the phenotypic trait space. The traits serve as effective indicators within the UMAP, enabling clear differentiation and understanding of the complex, nonlinear relationships among the samples. Using UMAP, we find that these plants span a continuous space characterized by different high variance traits, including the mean convex hull area (Fig. 11A) and the median lateral root angles (Fig. 11B).

## Conclusion

We have developed pipelines for efficient and accurate root trait phenotyping using a pose estimation-based approach. While previous approaches have leveraged landmark detection [21], here we establish the feasibility of a segmentation-free, pose estimation-based pipeline for RSA phenotyping.

In summary, the key contributions of this work include:

1. We use SLEAP [31] to train pose estimation models to localize and group landmarks on primary, crown, and lateral roots across a range of plant species, including the crop plants soybean (*G. max*), rice (*O. sativa*), canola (*B. napus*), and the model plant Arabidopsis (*A. thaliana*). We show that our pipelines can detect root landmarks with 0.087% root length or 0.540 mm (rice primary root 3 DAG) to 0.984% root length or 0.843 mm (Arabidopsis lateral 7 DAG) median localization error (Fig. 5).

2. We developed a plant RSA trait extraction tool (*sleap-roots*) that can extract up to 1,035 traits per plant using predicted landmarks (Fig. 4). We show that there is a high correlation between traits (root length and tip depth) from the fully automated *sleap-roots* pipeline with independently and manually measured traits in Fiji across 1,901 plants (Fig. 6). We further show that the full set of up to 1,035 computed traits are highly accurate, with 96.2% (canola; *n* = 107 plants) to 99.5% (rice; *n* = 270 plants) of individual trait values derived from fully automated inference falling within 1 SD from those derived from manually proofread landmarks (Fig. 7).

3. We show that accurate pose estimation-based root landmark detection and grouping can be achieved with <100 labeled images for most datasets (Fig. 8), and is 1.5× faster to annotate and 10× faster to train and predict than segmentation-based approaches (Fig. 9).

4. We demonstrate the applicability of pose estimation-derived root traits for common downstream analyses, such as genotype classification (Fig. 10) and large-scale phenotypic trait mapping (Fig. 11).

5. To inform adoption by practitioners interested in measuring specific traits, we provide an exhaustive list of traits and their estimated accuracies using our automated pipeline, as well as species-specific annotation protocols (Supplementary Materials).

6. To encourage reproducibility and extensions to our work, we make all data, code, and models openly available: https://github.com/talmolab/sleap-roots and https://osf.io/k7j9g/.

### Limitations and future directions

While this work demonstrates clear advantages to pose estimation-based plant phenotyping, we note that this approach has inherent limitations:

1. We approximate root centerlines using a fixed number of landmarks per model, which means that complex root curvatures will not be as well captured in some cases. Future work may be able to generalize our pose estimation algorithm to variable numbers of landmarks to resolve this.

2. While the vast majority of traits computed in segmentation-based pipelines are also recovered in our approach, root widths are not as reliably and densely estimated using our lateral root-matching method as would be possible via segmentation since we explicitly do not predict root boundaries. Future work combining segmentation with pose estimation may resolve this limitation.

3. Our pose estimation approach relies on landmark detection and grouping in 2D images, making this step of the pipeline readily compatible with 2D growth systems (e.g., plates) as has been shown previously [30]. While our explicit representation of connectivity is more robust to self-intersections than segmentation-based postprocessing (e.g., skeletonization and connected components), dense and highly overlapping root systems are challenging in either case. Here, we mitigate this by computing summary measures of traits across 72 different viewpoints using a cylindrical imaging system (RADICYL [10]), under the assumption that projective and occlusive ambiguities are resolvable in sufficient views. This will not be the case in single-view imaging systems, and self-intersections may present more substantial challenges in 2D growth systems. We expect that bottom-up pose estimation will be beneficial in systems with partial occlusions, like rhizoboxes, where disconnected root segments, obscured by dirt, can be logically reconnected by learning the plant’s overall morphological structure. Regardless of the imaging or growth system, we expect that our approach will perform most optimally on plants in their early developmental stages that exhibit a consistent skeletal structure with fewer self-intersections, occlusions, and projective ambiguities.

4. For manually measured root traits in Fiji, the annotator selected one frame among the 72 frames that had the fewest occlusions. As we do not have a comparable approach to a priori select the frame with the optimal viewing angle, we evaluated the SLEAP-based root trait estimation accuracy, using the median value of the trait summarized across all 72 frames to reflect the actual operating mode of our approach. Future work comparing *sleap-roots* traits to manual annotation may provide more nuanced views on the automated system performance by including repeated manual trait measurements on the same images and measurements across all images to circumvent the frame selection bias.

5. Despite our use of multi-view data, we do not estimate root landmarks in 3D. This is achievable using triangulation [52], but will require robust tracking of detections across views [53]. Datasets with heavy occlusion from overlapping roots, such as the rice crown 10 DAG model (Fig. 5C), would benefit the most from root traits using 3D reconstructions. We note, however, that many traits are well estimated in 2D (e.g., ordinal traits like root counts, or unambiguous spatial traits like root depth), which may make 3D reconstruction inadvisable for some experiments given the technical complexity of implementing a 3D imaging system.

6. As mentioned in the Materials and Methods, all models were trained using the same hyperparameters informed by previous work [30]. In the future, optimal hyperparameters could be fine-tuned for these datasets, which may considerably improve performance. This would make the most difference for Arabidopsis models (Fig. 5C and Fig. S2), which have smaller and thinner roots than the crop plants.

## Supplementary Materials

Supplementary 1 Figs. S1 and S2 Tables S1 to S6 Annotation Protocols
