# Supplementary material for: Fast and Efficient Root Phenotyping via Pose Estimation
Source: Plant Phenomics. 2024 Apr 12;6:0175. doi: 10.34133/plantphenomics.0175 (PMC11020144; doi:10.34133/plantphenomics.0175)

Arabidopsis Quality Control and Labeling in SLEAP

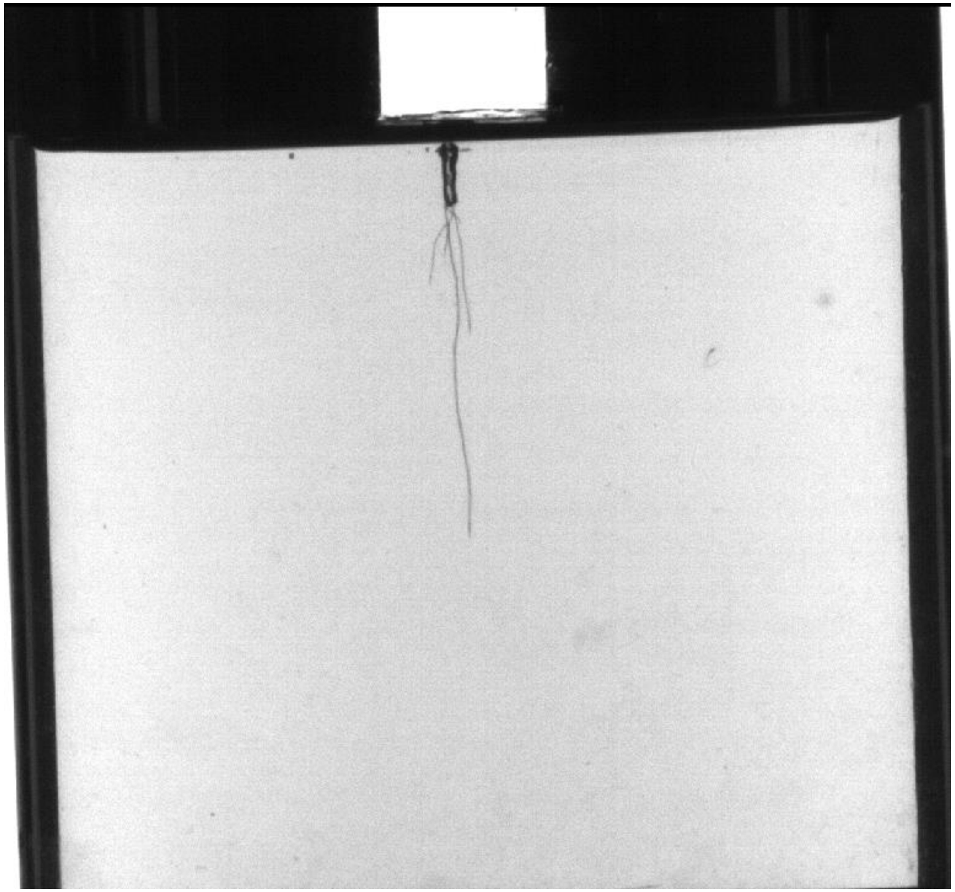

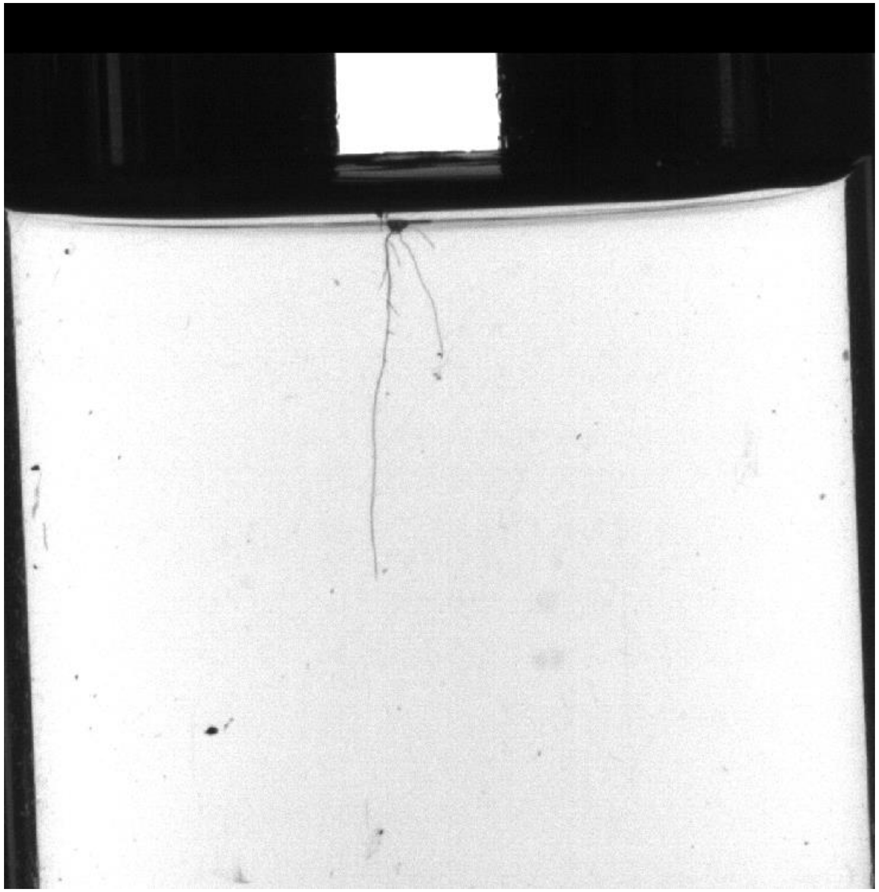

Overview

- Quality control
  - Protocol
  - Codes
  - Examples
- Arabidopsis Models
  - Primary Root Model and Skeleton
  - Lateral Roots Model and Skeleton
- General Arabidopsis Labeling Rules
  - Key Ideas
  - Tip and Base Definition
  - Prioritizing Roots
  - Occluded and Small Roots
  - Bubbles
  - Toggling Visibility: Bases, Tips, Holes

SLEAP Quality Control (QC) Protocol

- Before proofreading in sleap, QC must be done
- Meet beforehand to discuss specific QC requirements for individual experiments
  - Make sure there is consensus between everyone involved in QC and ideally do it together
- Do QC on the final day scan
  - If you do this before the final scan, please write the age you QC'd plant in column "QC_age"
- Do QC based on QC codes on following slide
- If replicate passes quality control, record a 0 in the "QC" column in the master data sheet for the experiment and leave "QC_code" column blank
- If replicate does not meet quality control requirements and needs to be excluded, mark 1 in "QC" column and record corresponding QC code in "QC_code" column

QC_code	Description	Notes/Details (vary based off of experiment)
**cont**	Excessive contamination engulfing plant	• Plants can have some contamination and not be QC'd out as long as you can see most of the root not contaminated in some frames<br>• As long as base of root is not contaminated, include plant (don't QC)
**sub**	Whole plant submerged	
**jig**	Media jiggly	
**ori**	Incorrect orientation	• Careful: distinguish poor growth and orientation, growing sideways or up is **ori** not **pg**
**germ**	Poor germination	• No germination or just a cotyledon nub grown (any growth past cotyledon nub is **pg** not **germ**)
**dead**	Unhealthy or dead	
**adv**	Too many adventitious roots	• Adventitious root s are different from primary or lateral roots. Models are not trained on adventitious roots so too many can result in error.
**miss**	Cylinder missing	• Only use if you can't find the scans for the barcode (naming individual cylinder). If you need to throw away a cylinder in the middle of an experiment note the date and QC_code
**pg**	Poot growth	• In experiments where we expect to see no grwth we will be less strict with this/define new conditions

Arabidopsis Models

Primary Root Model
Lateral Roots Model

- Arabidopsis is a dicot, it can jave one primary root and multiple lateral roots
- Arabidopsis has two SLEAP models one for primary roots and one for lateral roots

Arabidopsis Models: Primary Root Model

- Primary root defined as longest root
- Only one root should be labeled in this model

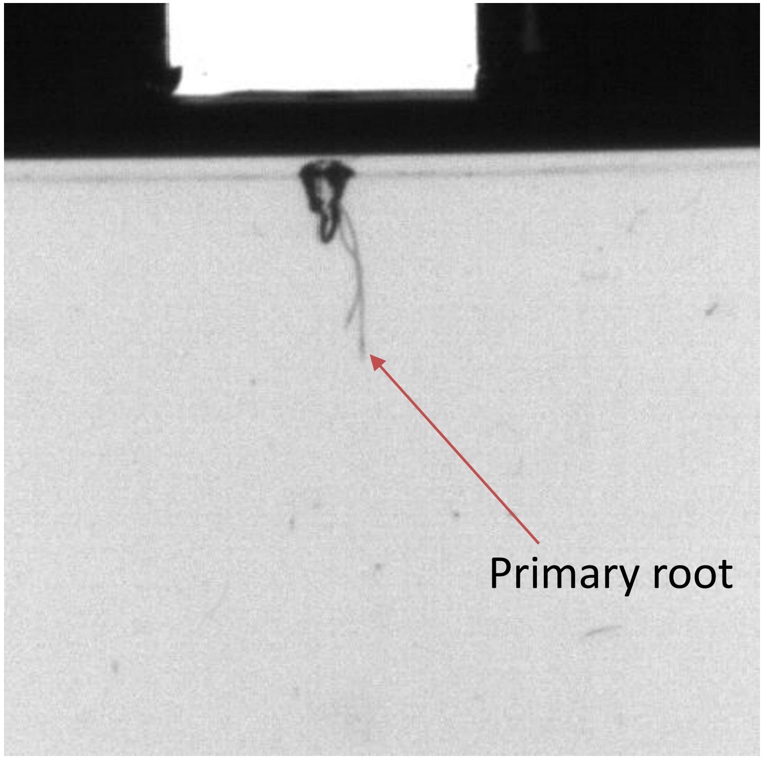

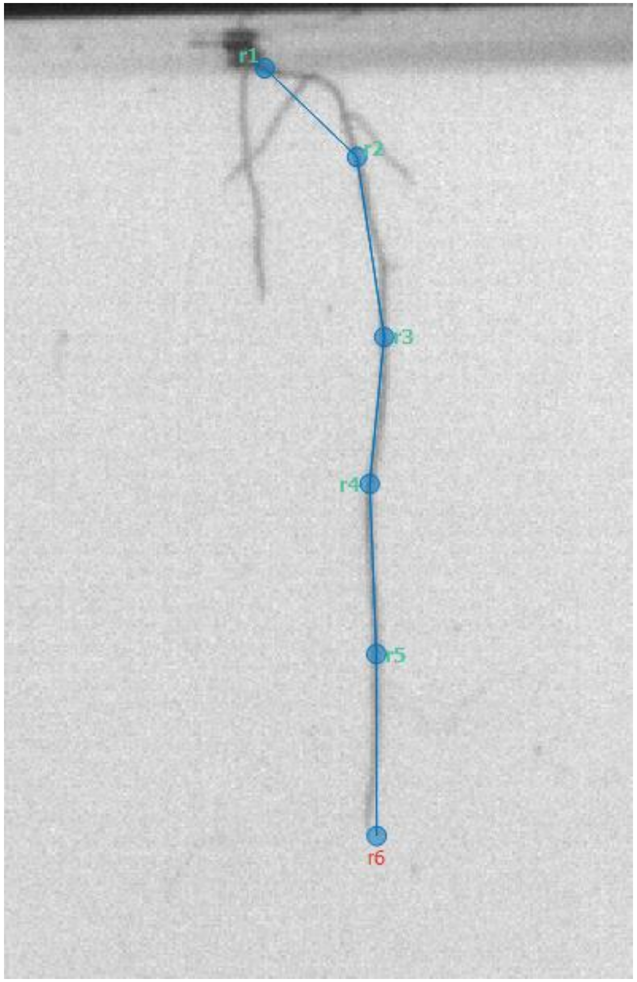

Arabidopsis Models: Primary Root Model Skeleton

- Roots are labelled using 6-node trees, which form a line, r1 is the base, r6 is the tip
  - Nodes should be equally spaced lengthwise along each root.
  - Nodes should be centered widthwise on the root.
    - Zoom in to make sure they're centered

"node_names": ["r1", "r2", "r3", "r4", "r5", "r6"],
"edge_inds": [[0, 1], [1, 2], [2, 3], [3, 4], [4, 5]]

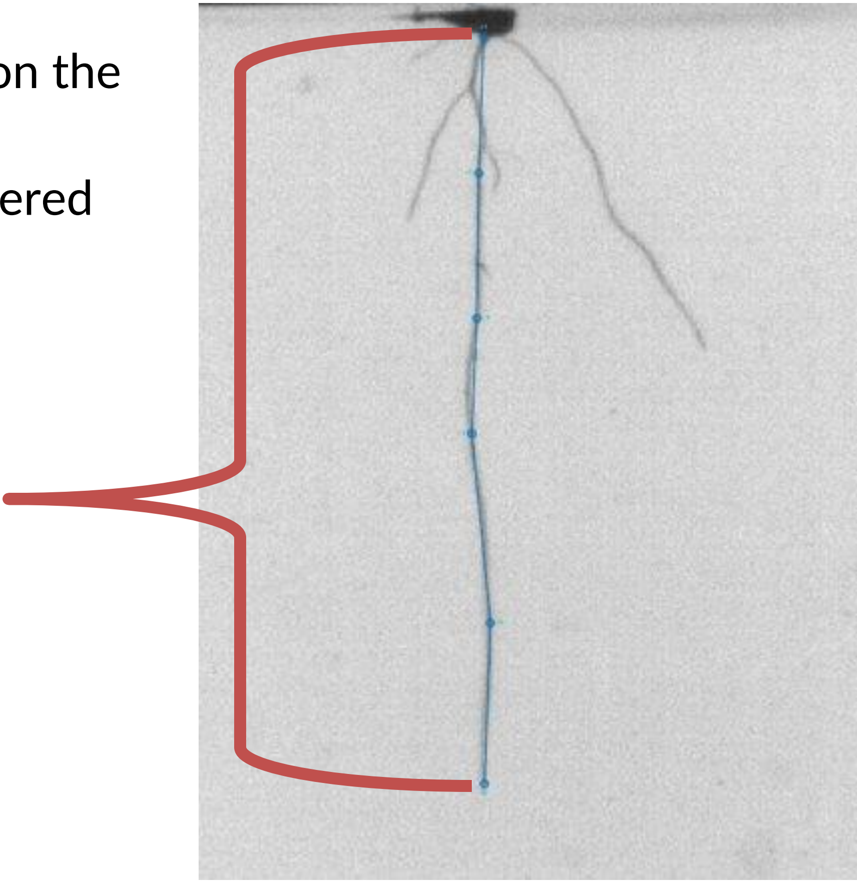

Arabidopsis Models: Lateral Roots Model

- At this age, lateral roots are all other roots besides the longest one
- Lateral roots grow out of the primary root
- All roots labeled in image to the right are lateral roots

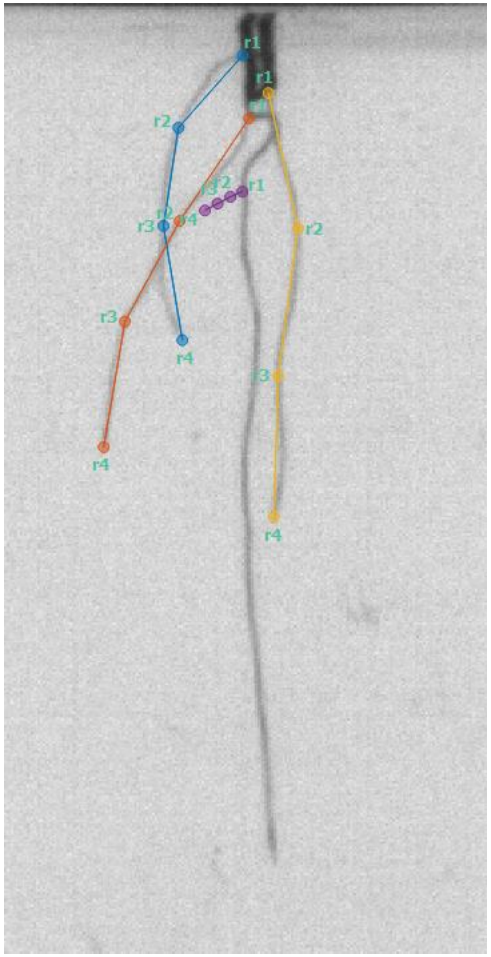

Arabidopsis Models: Lateral Roots Model Skeleton

- Roots are labelled using 4-node trees, which form a line. r1 is the base, r4 is the tip
  - Nodes should be equally spaced lengthwise along each root.
  - Nodes should be centered widthwise on the root.
    - Zoom in to make sure they’re centered

"node_names": ["r1", "r2", "r3", "r4"],
"edge_inds": [[0, 1], [1, 2], [2, 3]]

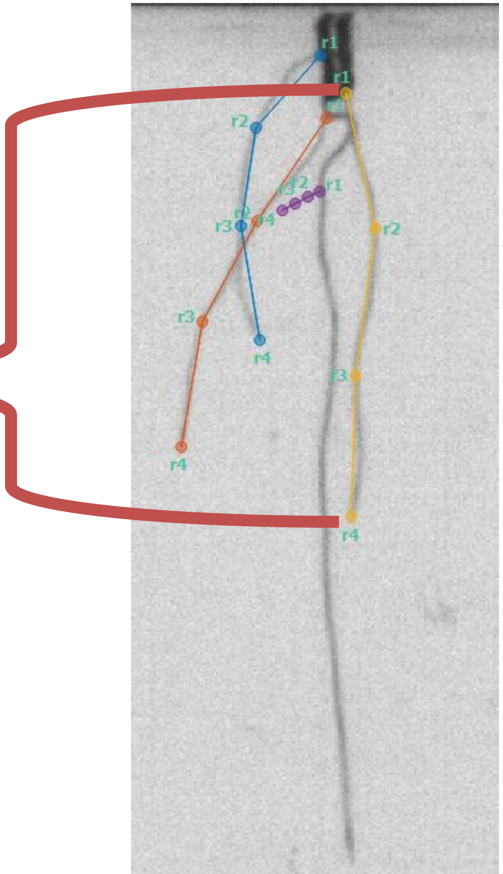

General Arabidopsis Labeling Rules: Key Ideas

Save frequently
- Changes are NOT automatically saved

Consistent labels
- if labels are not consistent, they will have to be re-done
- Go slowly and carefully rather than quickly
- If more than one person is labeling, review rules for crop and experiment beforehand and record who labels which replicates

Don't over label
- Older roots are very complicated
    - Review specific experiment focus to decide priorities but in general prioritize:
        - larger roots
        - more visible roots
        - roots most important to overall root system architecture
- Minimize crisscrossing labels

General Arabidopsis Labeling Rules: Tip Definition

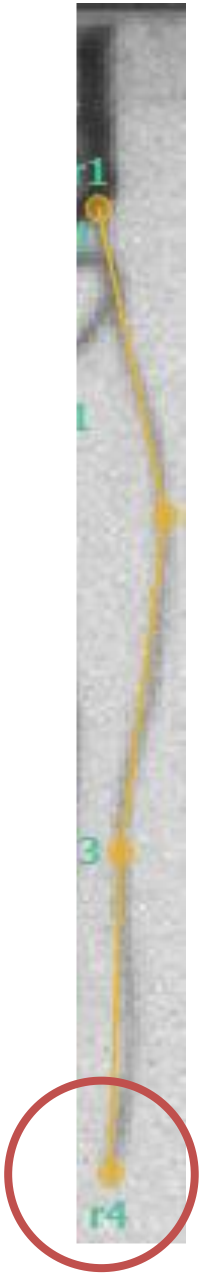

- The tip of the root is the last visible point, and should be labeled with r6 (circled in purple)
  - Remember to zoom in for precision

General Arabidopsis Labeling Rules: Base Definition

- The **base** of the root is the **first visible point** on an individual root and should be labelled as **r1**.
  - If the bases of two roots overlap, r1 on one of the roots should be moved down to the first distinguishable point of the root that does not overlap with another root.
  - The bases of thicker and longer roots should be prioritized and kept higher instead of moved down.
  - If a bubble or a hole obstructs the primary root, start r1 as close as to where primary root should be

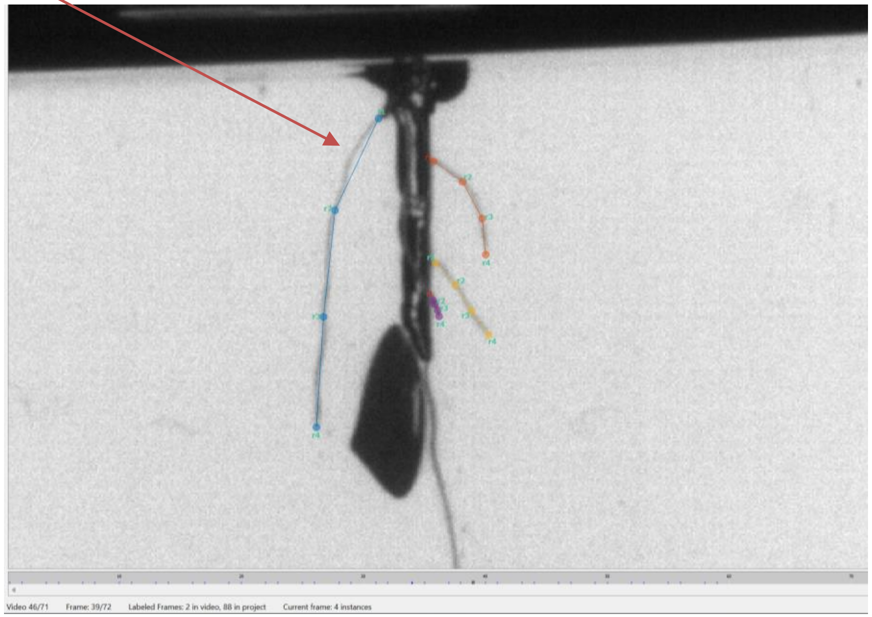

General Arabidopsis Labeling Rules: Occluded Roots

- If root is small and 75% or more occluded by another larger root, don't label (circled in red to the right)

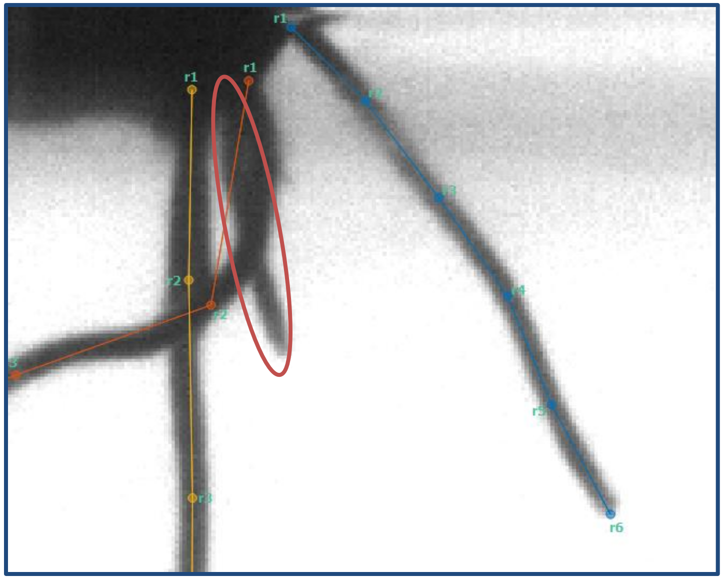

Example is rice plant

General Arabidopsis Labeling Rules: Small Roots

- When roots are too small to distinguish if they are roots or specs, then do not label them.
  - Circled lateral root in left example is big enough to label
  - Red circled lateral roots in right example are too small to label

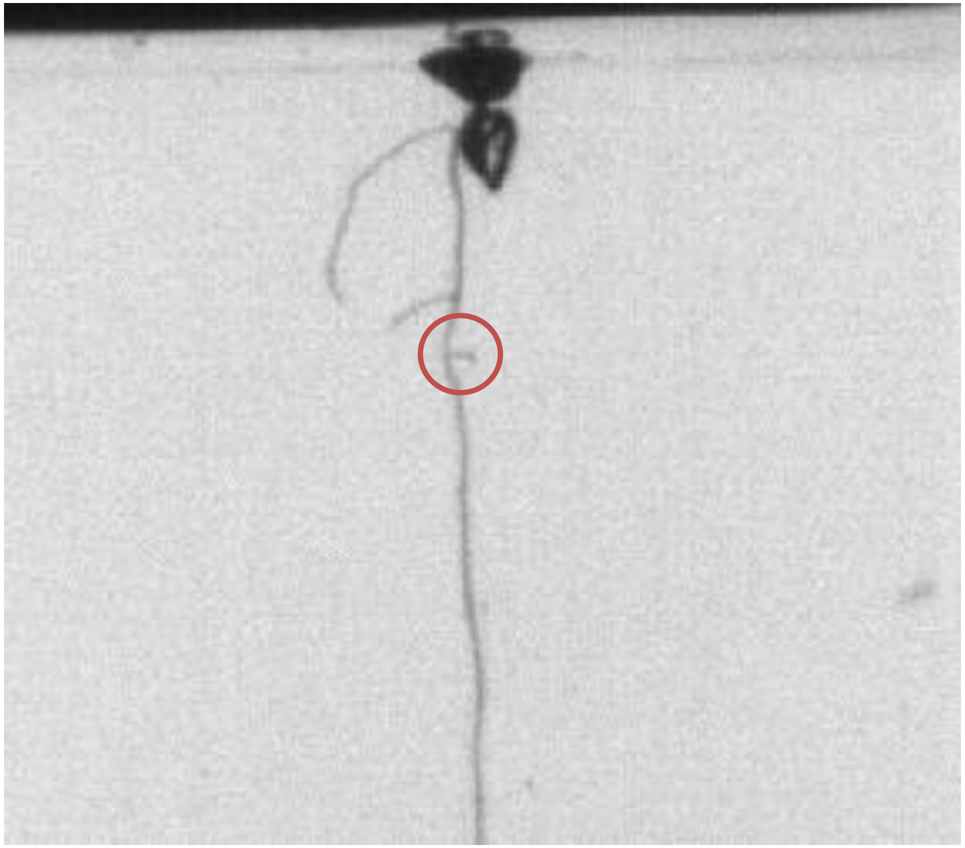

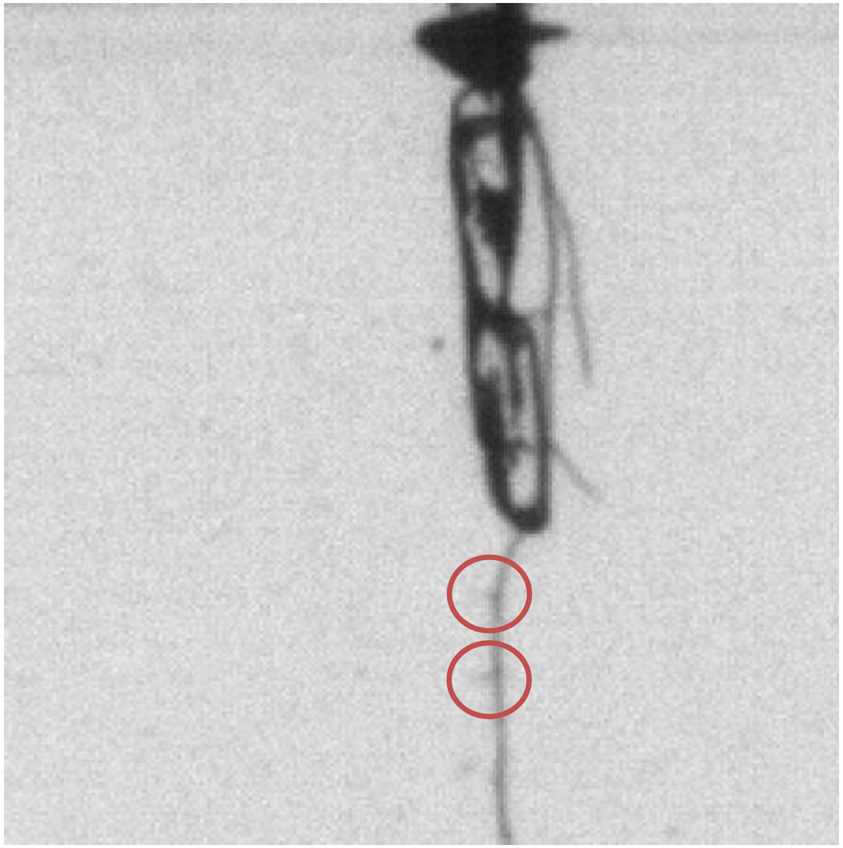

General Arabidopsis Labeling Rules: Labeling Around Bubbles

- If a bubble is obstructing a root but you're sure the root goes under the bubble, you can place a node on top of the bubble
  - Make sure to keep nodes evenly spaced
  - If you're not sure where the root ends/if it ends behind the bubble, shift to other frames with a similar angle where the root isn't hidden behind the bubble

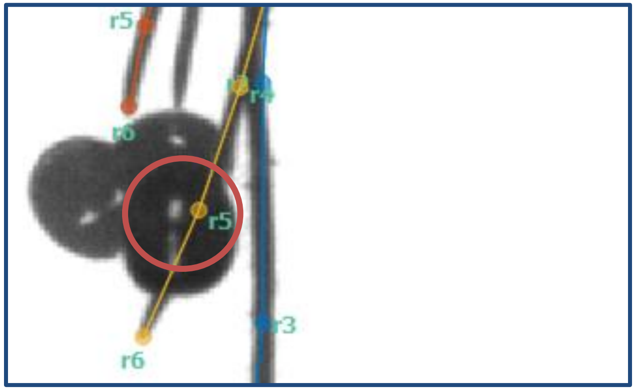

Example is a rice plant

General Arabidopsis Labeling Rules: Toggling Visibility - Bases

- Use sparingly, only when any of following conditions are met:
  - Toggle visibility of a node anytime it's on a part of the root that's not visible.
  - If two roots' bases overlap, you can either move the base of the lower-priority root down (TRY THIS FIRST), or toggle the visibility of the nodes above its first non-overlapping point

General Arabidopsis Labeling Rules: Toggling Visibility - Tips

- SLEAP uses landmark detection
  - ==> Tips are easily recognizable
- If you cannot see the tip, label the last point on the root as r5 and r6 as invisible
- Only do this if most of that root is visible (more than 75%). Otherwise, just do not label the root.

- In the example to the right the tip of the right most blue labeled root is occluded so r5 is on the last visible part of the root and r6 is toggled invisible

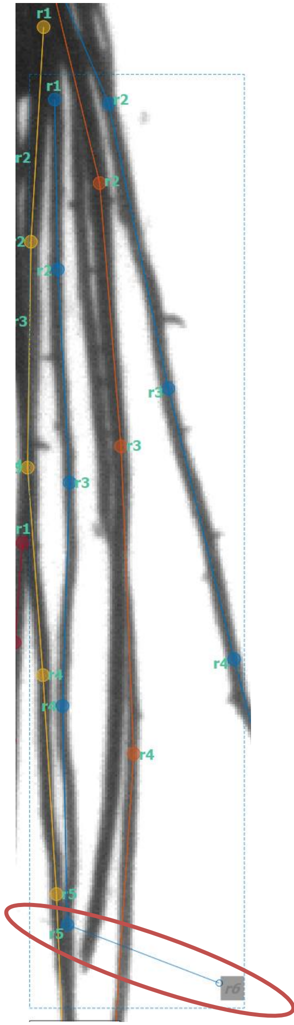

Example is rice plant

General Arabidopsis Labeling Rules: Toggling Visibility - Holes

- There cannot be any holes of visibility in a root.
- If a segment in the middle of a root is not visible, the nodes that are in the occluded part must be toggled invisible AND the rest of the nodes going one direction (towards r1 or r6) must also be toggle invisible
- Example on the right: r3 on the red root overlaps with the pink root, and in this case the pink root takes priority.
    - r3 on the red root must be toggled invisible.
    - Since it's in the middle of the root, either r4, r5, and r6 or r1 and r2 must also be toggled invisible to avoid a hole.

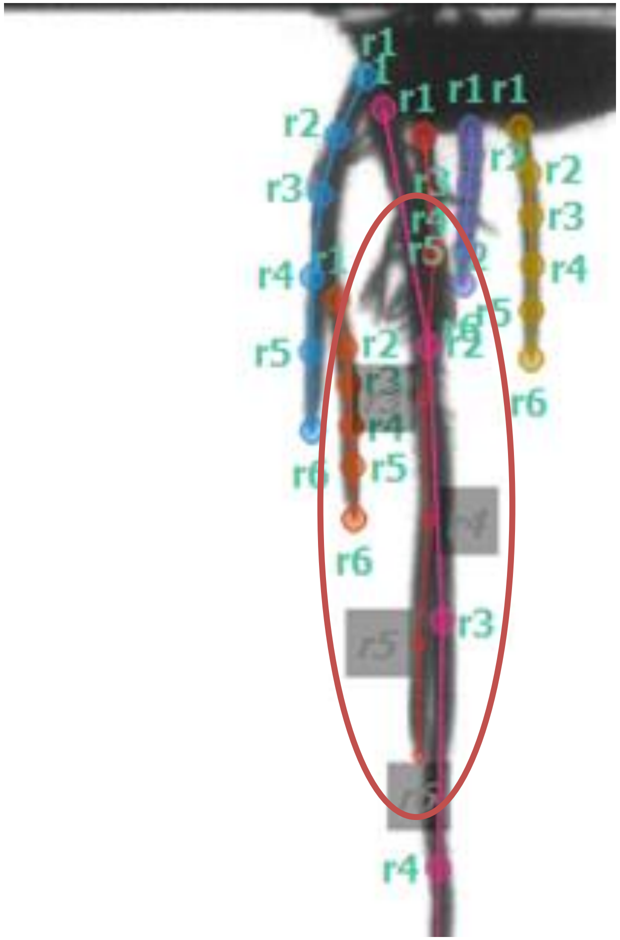

Example is rice plant

Canola Quality Control and Labeling in SLEAP

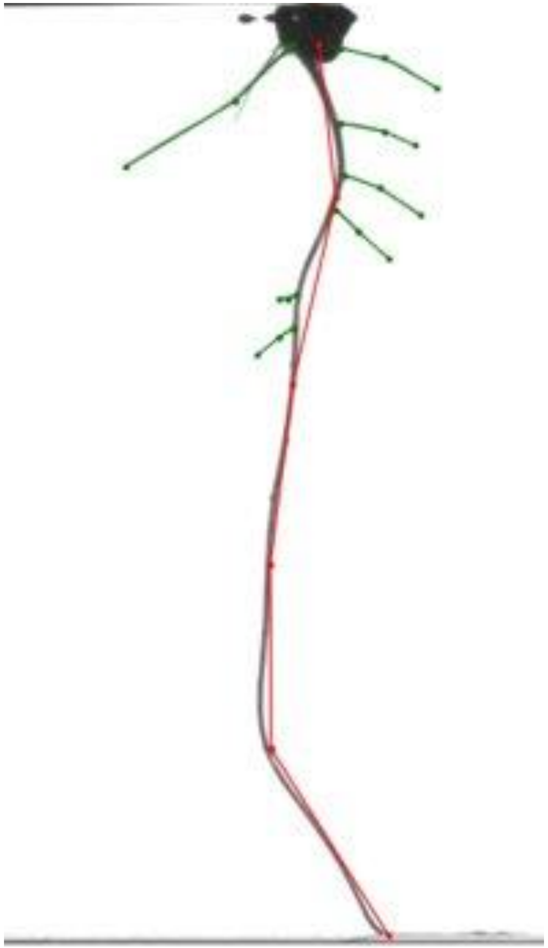

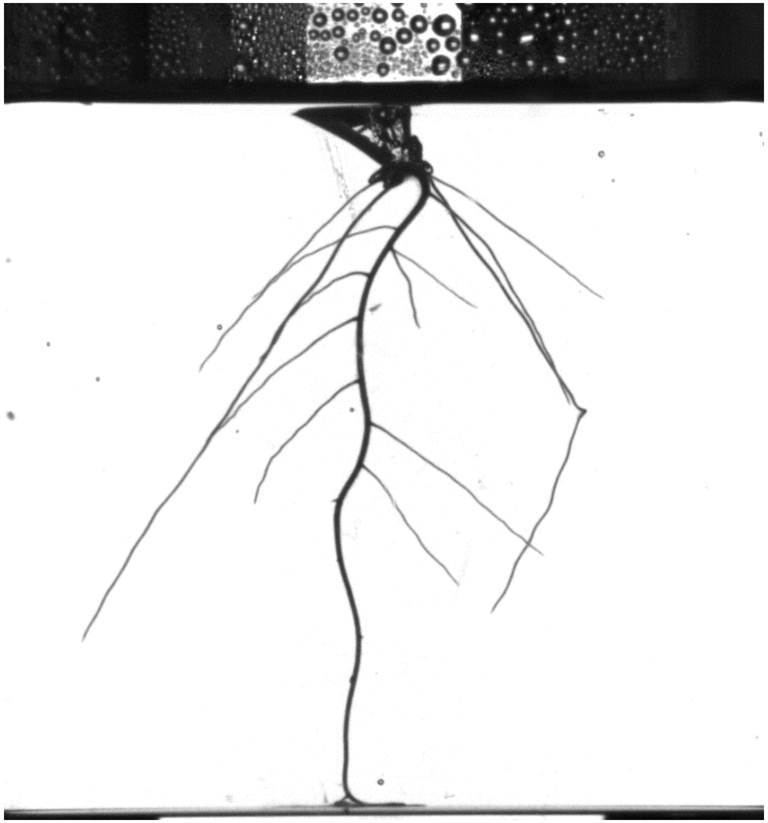

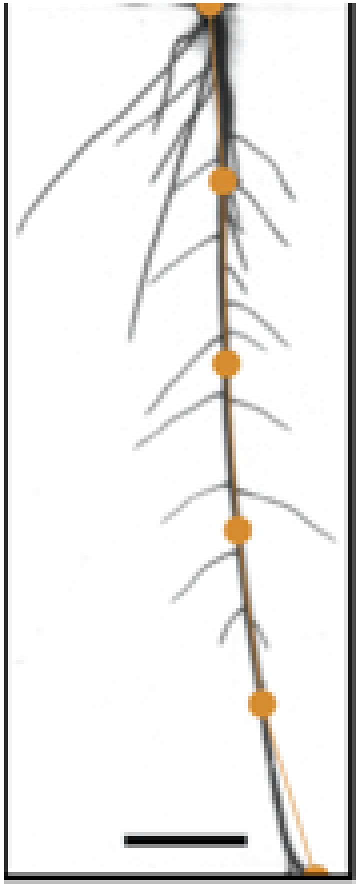

Overview

- Quality control
  - Protocol
  - Codes
  - Examples
- Soybean Models
  - Primary Root Model and skeleton
  - Lateral Roots Model and skeleton
- General Soybean Labeling Rules
  - Key Ideas
  - Tip and Base Definition
  - Prioritizing Roots
  - Occluded, Small Roots
  - Toggling Visibility: Bases, Tips, Holes
  - Labeling Around Bubbles and Contamination

SLEAP Quality Control (QC) Protocol

- Before proofreading in sleap, QC must be done
- Meet beforehand to discuss specific QC requirements for individual experiments
  - Make sure there is consensus between everyone involved in QC and ideally do it together
- Do QC on the final day scan
  - If you do this before the final scan, please write the age you QC'd plant in column "QC_age"
- Do QC based on QC codes on following slide
- If replicate passes quality control, record a 0 in the "QC" column in the master data sheet for the experiment and leave "QC_code" column blank
- If replicate does not meet quality control requirements and needs to be excluded, mark 1 in "QC" column and record corresponding QC code in "QC_code" column

QC_code	Description	Notes/Details (vary based off of experiment)
**cont**	Excessive contamination engulfing plant	• Plants can have some contamination and not be QC'd out as long as you can see most of the root not contaminated in some frames<br>• As long as base of root is not contaminated, include plant (don't QC)
**sub**	Whole plant submerged	
**jig**	Media jiggly	
**ori**	Incorrect orientation	• Careful: distinguish poor growth and orientation, growing sideways or up is **ori** not **pg**
**germ**	Poor germination	• No germination or just a cotyledon nub grown (any growth past cotyledon nub is **pg** not **germ**)
**dead**	Unhealthy or dead	
**adv**	Too many adventitious roots	• Adventitious root s are different from primary or lateral roots. Models are not trained on adventitious roots so too many can result in error.
**miss**	Cylinder missing	• Only use if you can't find the scans for the barcode (naming individual cylinder). If you need to throw away a cylinder in the middle of an experiment note the date and QC_code
**pg**	Poot growth	• In experiments where we expect to see no grwth we will be less strict with this/define new conditions

QC Examples: **cont** (Contamination)

- QC for if contamination engulfs base of plant
- And/or if contamination occludes large portion of roots on most frames

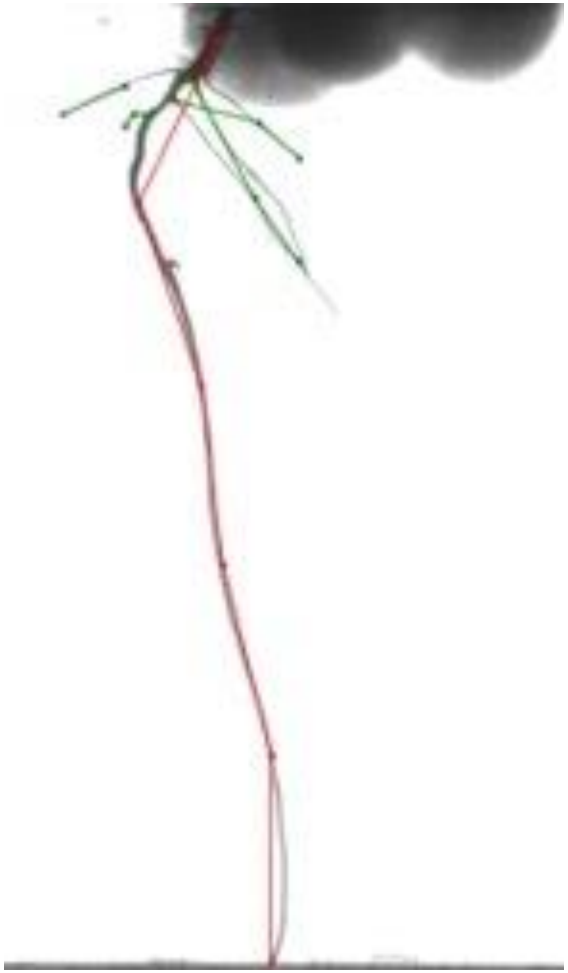

QC Examples: **pg** (poor growth)

- Both plants are 9 days old and are very small for this timepoint in comparison to other individuals of the same genotype and environmental conditions
- Plants look stunted for canola

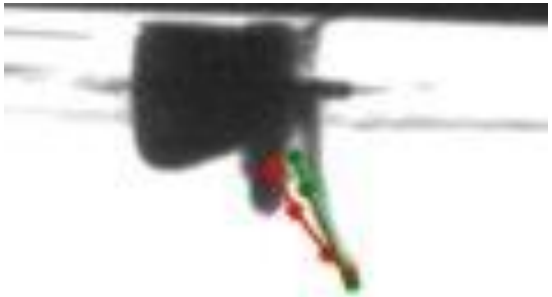

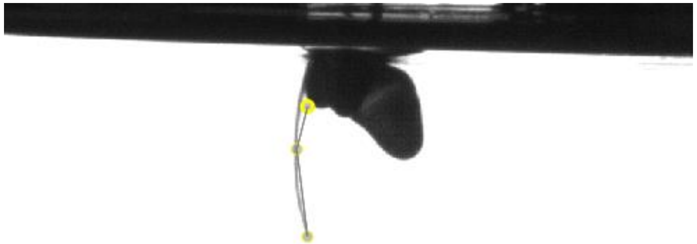

QC Examples: **ori** (orientation)

- QC for orientation if shoots is growing into media or plants is growing in incorrect orientation
- In right examples the shoot growing into the media is circled in green
    - Specifically, the leaves are growing into the media in both examples

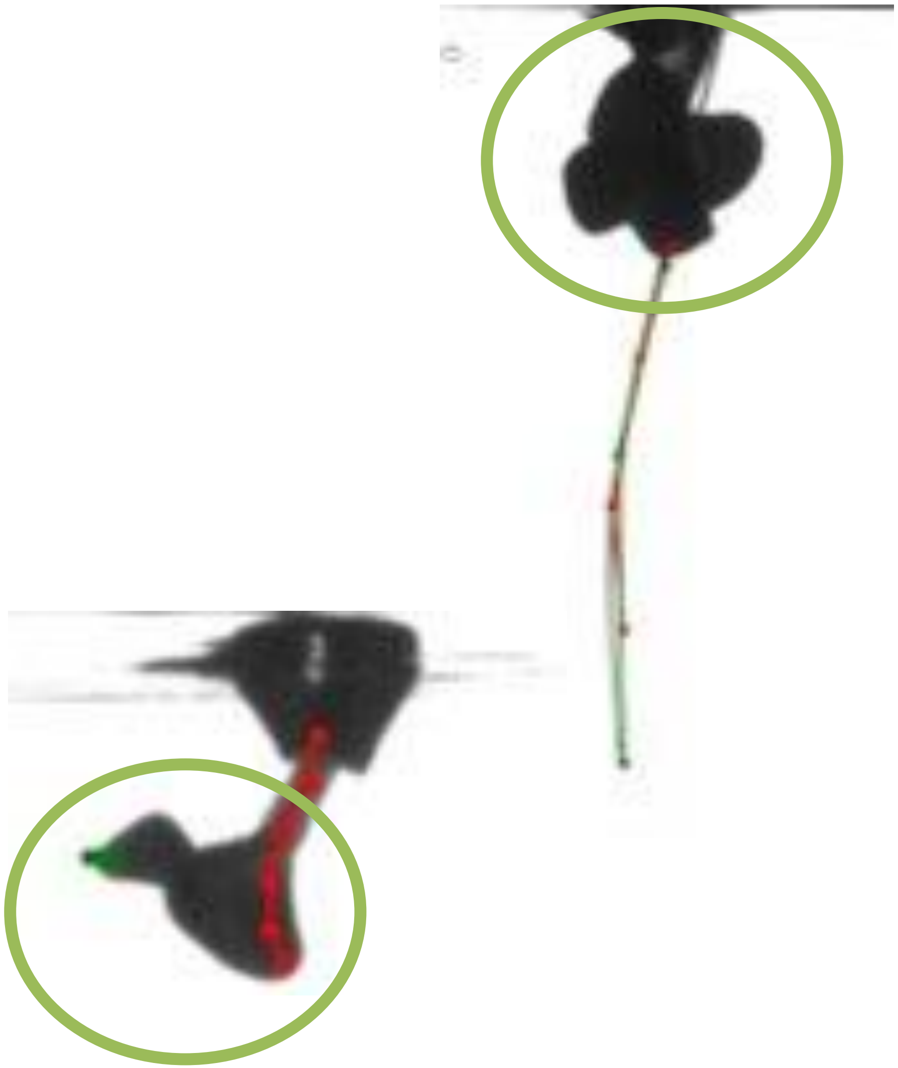

QC Examples: **sub** (submerged)

- In right example the seed is fully submerged in the media (top of media is the black line at the top of the image)
- Seed should be intersecting the top of the media, if not QC the plant being submerged

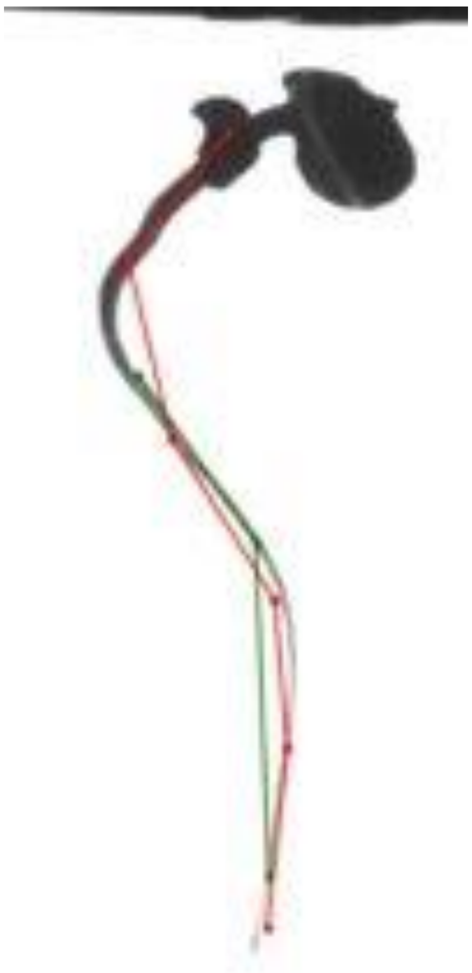

Canola Models

Primary Root Model
Lateral Root Model

- Canola is a dicot, it can have one primary root and multiple lateral roots
- Canola has two SLEAP models one for primary roots and one for lateral roots

Primary Root is in Red
Lateral Roots are in Green

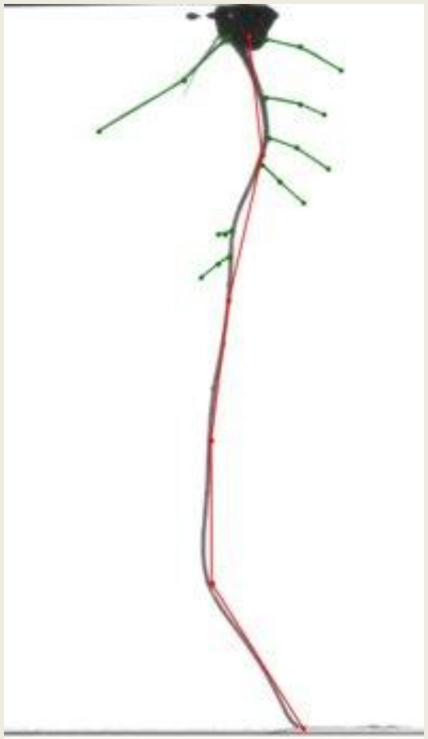

Canola Models: Primary Root Model and Skeleton

- In Canola, primary roots are usually the largest, thickest, most gravitropic root.
- Primary roots grow out of the seed whereas lateral roots grow out of the primary root

- Primary roots are labelled using 6-node trees, which form a line
- r1 is the base node and r6 is the tip node
- Nodes should be equally spaced lengthwise along each root.
- Nodes should be centered widthwise on the root.
- Zoom in to make sure they're centered

"node_names": ["r1", "r2", "r3", "r4", "r5", "r6"],
"edge_inds": [[0, 1], [1, 2], [2, 3], [3, 4], [4, 5]]

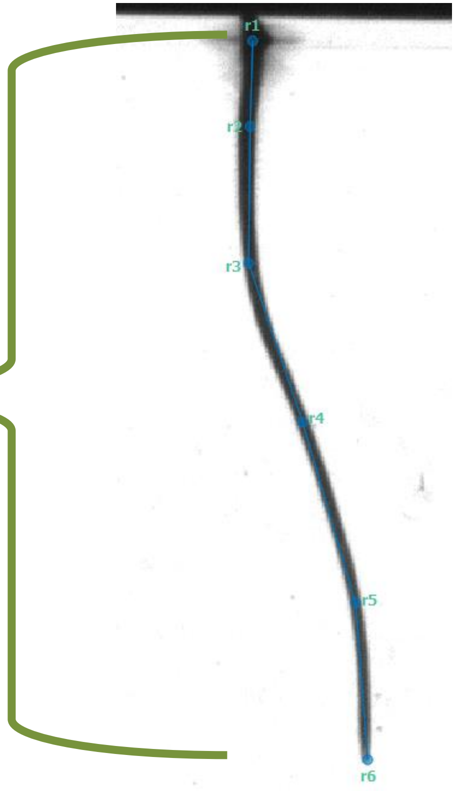

Canola Models: Lateral Roots Model and Skeleton

- Lateral roots grow out of the primary root and in general are thinner and shorter than the primary root at this age

- Lateral roots are labelled using 3-node trees, which form a line
- r1 is the base node and r3 is the tip node
- Nodes should be equally spaced lengthwise along each root.
- Nodes should be centered widthwise on the root.
- Zoom in to make sure they're centered

```
"node_names": ["r1", "r2", "r3"]
"edge_inds": [[0, 1], [1, 2]]
```

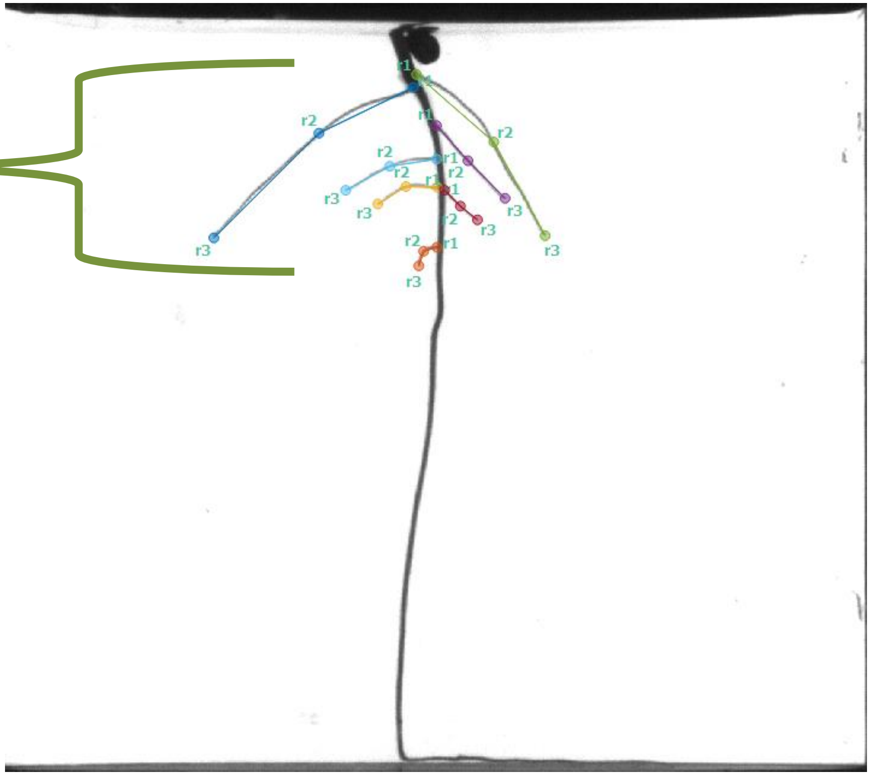

General Canola Labeling Rules: Key Ideas

Save frequently

- Changes are NOT automatically saved

Consistent labels

- if labels are not consistent, they will have to be re-done
- Go slowly and carefully rather than quickly
- If more than one person is labeling, review rules for crop and experiment beforehand and record who labels which replicates

Don’t over label

- Older roots are very complicated
  - Review specific experiment focus to decide priorities but in general prioritize:
    - larger roots
    - more visible roots
    - roots most important to overall root system architecture
- Minimize crisscrossing labels

General Canola Labeling Rules: Tip Definition

- The tip of the root is the last visible point, and should be labelled with r3 in the lateral root model and r6 in the primary root model (lateral root example below, tips circled in green) .
  - Remember to zoom in for precision

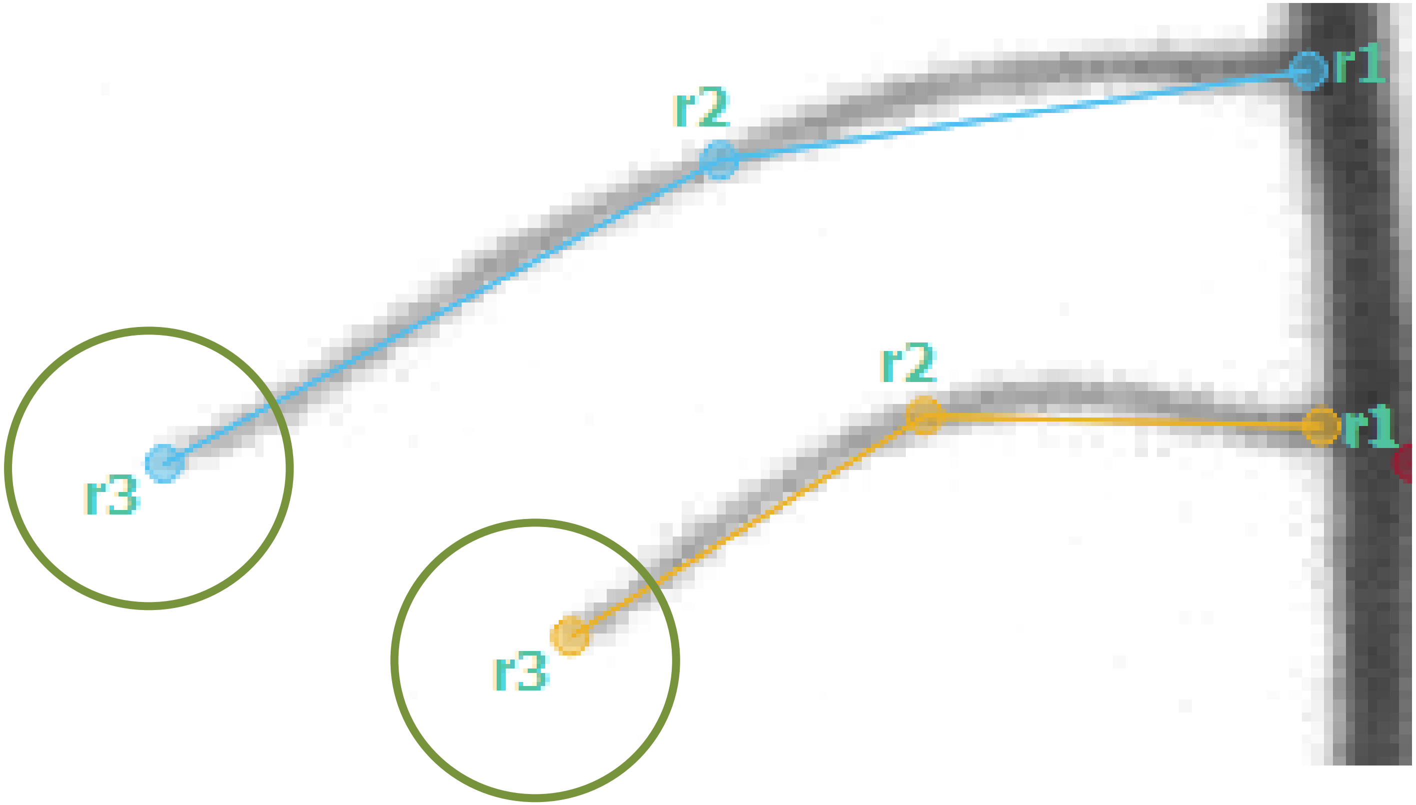

General Canola Labeling Rules: Base Definition

- The **base** of the root is the **first point visible** on a primary or a lateral root and should be labelled as **r1**.
- **For a primary root**, this point is where the primary root reaches the seed or the top most visible part of the root structure.
- **For a lateral root**, this point is where the lateral root meets the primary root
- If the bases of two roots overlap, r1 on one of the roots should be moved down to the first distinguishable point of the root that does not overlap with another root.
  - Or toggle r1 invisible
- The bases of thicker and longer roots should be prioritized and kept higher instead of moved down.
- Bases are circled in green in example below

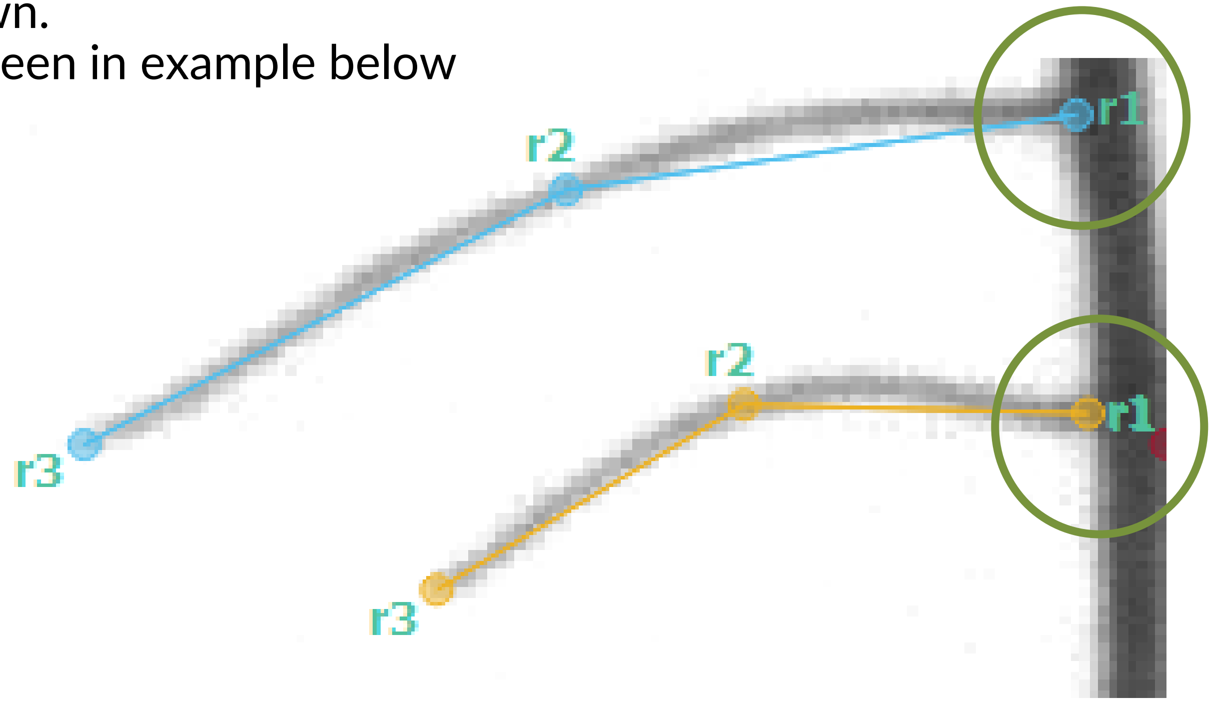

General Canola Labeling Rules: Prioritizing Roots - Size

- Prioritize larger roots
- If two roots have similar thickness/length, prioritize the one that is less obstructed by other roots
  - The non-prioritized one will be prioritized in a different frame
- In the example below, the bases of the orange and blue root are overlapping
- Since the orange root is larger and more important than the blue root overall to the root system architecture, the orange root is prioritized.
  - So the r1 of the blue root is moved downwards to the first non-overlapping section

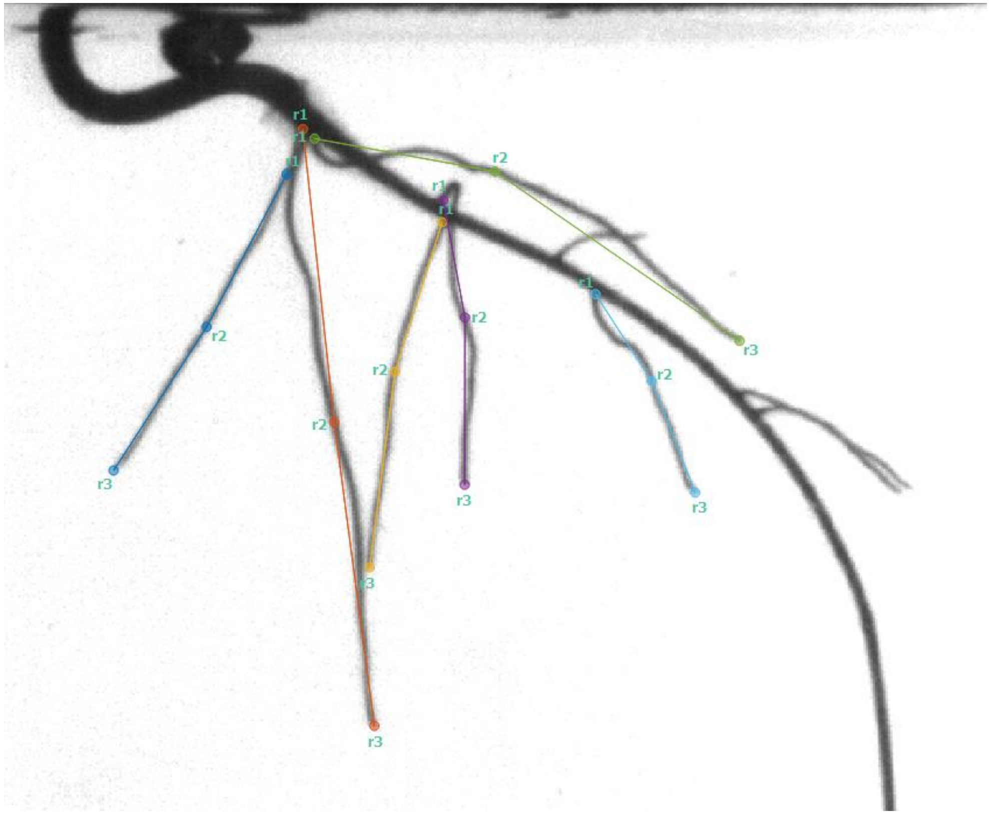

General Canola Labeling Rules: Occluded and Small Roots

- Don't label a root if it's ~75% or more occluded by another root
- label the base of an occluded root if you can tell where that would be
- Here we don't label the root behind the purple root because it's about 75% occluded by the purple root and to label it we would have to 2/3 toggle nodes invisible to avoid crisscrossing

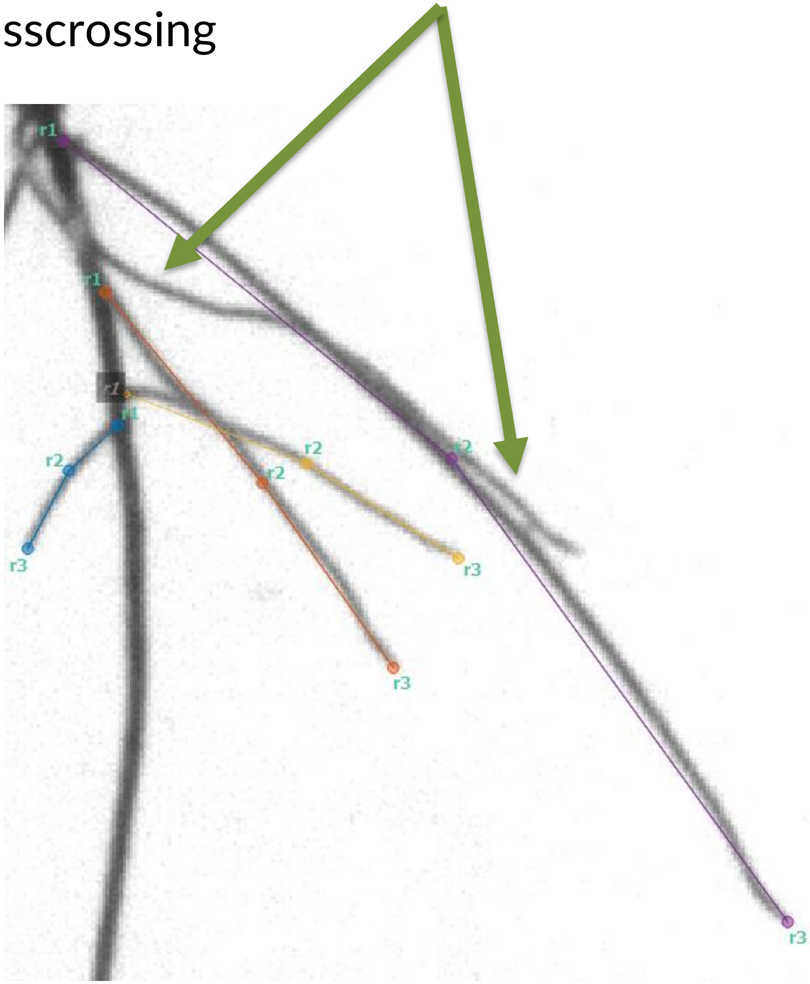

General Canola Labeling Rules: Toggling Visibility – Bases, Tips, Holes

- Use sparingly, only when any of following conditions are met:

Bases

- Toggle visibility of a node anytime it's on a part of the root that's not visible.
- If two roots' bases overlap, you can either move the base of the lower-priority root down (TRY THIS FIRST), or toggle the visibility of the nodes above its first non-overlapping point
- For an example of moving r1 downwards, see base definition/labelling slide

Tips

- SLEAP uses **landmark detection**
  - ==> Tips are easily recognizable
- **For primary roots**, If you cannot see the tip, label the last point on the root as r5 and r6 as invisible
- **For lateral roots**, If you cannot see the tip, label the last point on the root as r2 and r3 as invisible
- Only do this if most of that root is visible (more than 75%). Otherwise, just do not label the root.

Holes

- There cannot be any holes of visibility in a root.
- If a segment in the middle of a root is not visible, the nodes that are in the occluded part must be toggled invisible AND the rest of the nodes going one direction (towards r1 or towards r6) must also be toggle invisible

General Canola Labeling Rules: Labeling Around Bubbles and Contamination

- If a bubble is obstructing a root but you're sure the root goes under the bubble, place a node on top of the bubble
- Make sure to keep nodes evenly spaced
- If you're not sure where the root ends/if it ends behind the bubble, shift to other frames with a similar angle where the root isn't hidden behind the bubble
- See example of node placed over bubbled circled in blue to the right

Rice Quality Control and Labeling in SLEAP

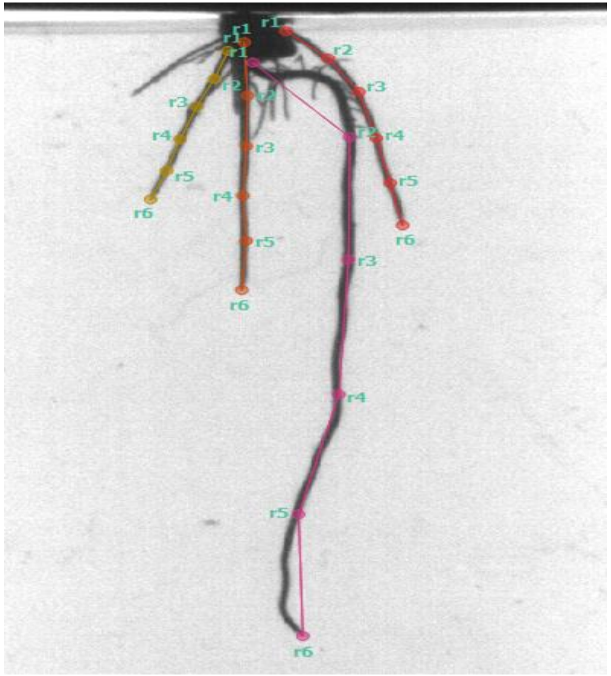

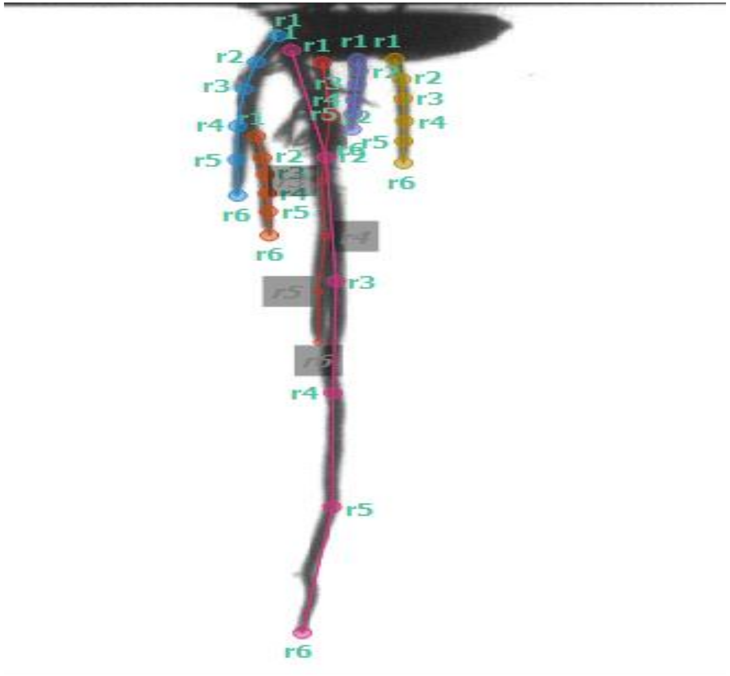

Overview

- Quality control
  - Protocol
  - Codes
  - Examples
- Rice Models
  - Primary Root Model
  - Crown Root Model
- General Rice Labeling Rules
  - Key Ideas
  - Skeleton of Rice
  - Tip and Base Definition
  - Prioritizing Roots
  - Occluded, Small Roots
  - Toggling Visibility: Bases, Tips, Holes
  - Bubbles
  - Shoots

SLEAP Quality Control (QC) Protocol

- Before proofreading in sleap, QC must be done
- Meet beforehand to discuss specific QC requirements for individual experiments
    - Make sure there is consensus between everyone involved in QC and ideally do it together
- Do QC on the final day scan
    - If you do this before the final scan, please write the age you QC'd plant in column "QC_age"
- Do QC based on QC codes on following slide
- If replicate passes quality control, record a 0 in the "QC" column in the master data sheet for the experiment and leave "QC_code" column blank
- If replicate does not meet quality control requirements and needs to be excluded, mark 1 in "QC" column and record corresponding QC code in "QC_code" column

QC_code	Description	Notes/Details (vary based off of experiment)
**cont**	Excessive contamination engulfing plant	• Plants can have some contamination and not be QC'd out as long as you can see most of the root not contaminated in some frames<br>• As long as base of root is not contaminated, include plant (don't QC)
**sub**	Whole plant submerged	
**jig**	Media jiggly	
**ori**	Incorrect orientation	• Careful: distinguish poor growth and orientation, growing sideways or up is **ori** not **pg**
**germ**	Poor germination	• No germination or just a cotyledon nub grown (any growth past cotyledon nub is **pg** not **germ**)
**dead**	Unhealthy or dead	
**adv**	Too many adventitious roots	• Adventitious root s are different from primary or lateral roots. Models are not trained on adventitious roots so too many can result in error.
**miss**	Cylinder missing	• Only use if you can't find the scans for the barcode (naming individual cylinder). If you need to throw away a cylinder in the middle of an experiment note the date and QC_code
**pg**	Poot growth	• In experiments where we expect to see no grwth we will be less strict with this/define new conditions

QC Examples: **cont** (Contamination)

- Contamination engulfs base of plant
- Contamination occludes large portion of roots on most frames

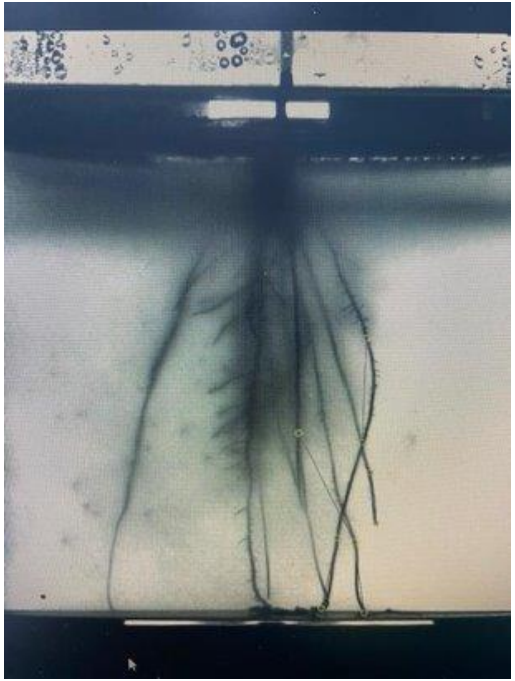

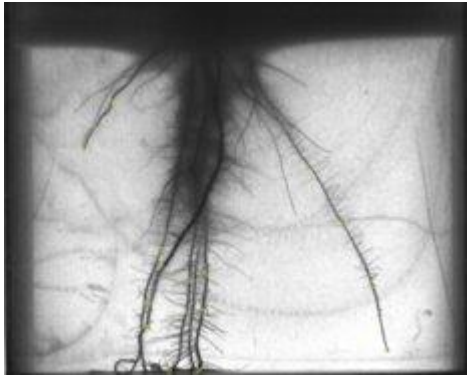

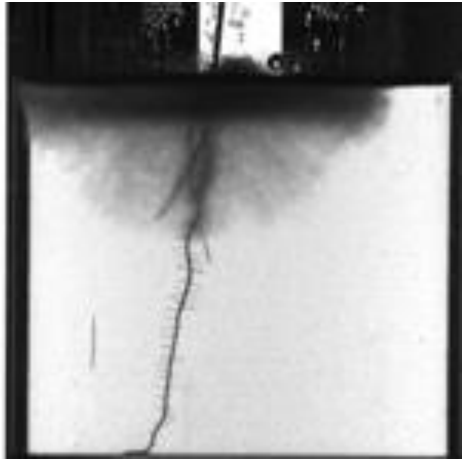

QC Examples: **ori** (Orientation)

- Shoot grew down into the media, considered poor orientation of seed at transplanting resulting in shoot growing down into media

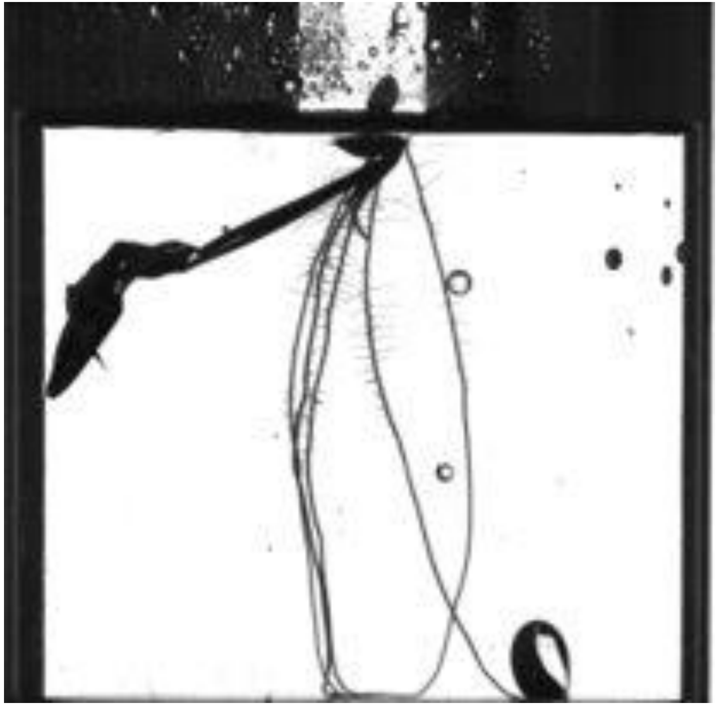

QC Examples: **pg** (poor growth)

- Plant is very small for timepoint in comparison to other individuals of the same genotype and environmental conditions
- Shoot growing into media and looks stunted for rice
  - This plant is also an **ori** QC because of the shoot growth into the media

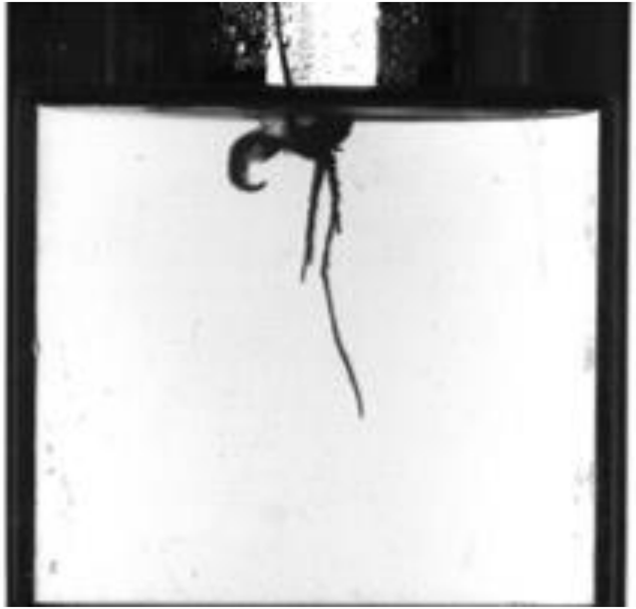

Rice Models

Primary Root Model
Crown Root Model

- Rice is a monocot, it can have many crown roots. The first emerging root and longest is the primary root.
- Rice has two SLEAP models one for crown roots + primary root and one for primary roots.

Rice Models: Primary Root Model

- Primary root defined as: longest root
- Only one root should be labeled in this model
- In 3 day-old plants for most individual cylinder replicates, the longest root is the only root visible

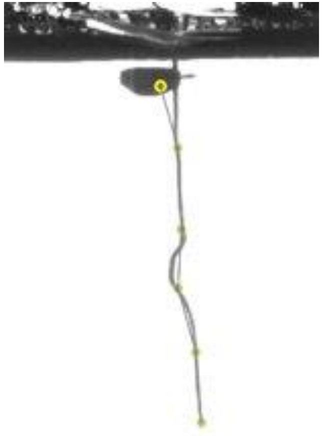

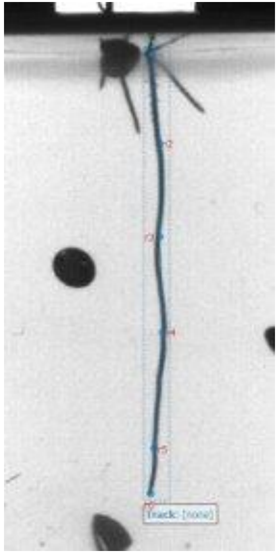

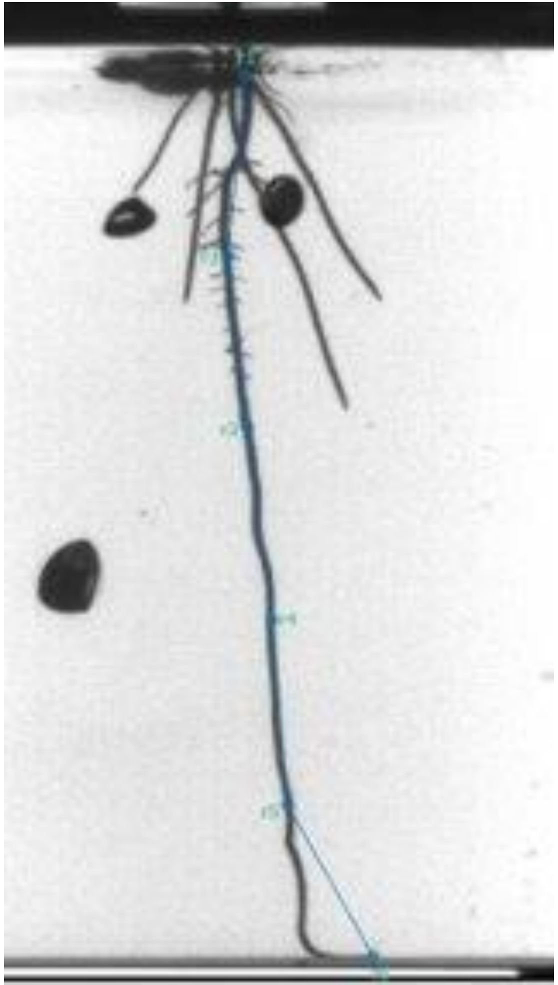

Rice Models: Crown Root Model

- Crown roots are the thick and dark roots
- Crown roots are not to be confused with the lateral roots
    - Lateral or hairy roots grow off the crown
    - Hairy roots are significantly shorter and thinner
- Only crown roots should be labeled

In example to the right:

- Hairy roots are circled in purple
- All other roots labeled in this image are crown roots (including the root in purple which would be labeled as the primary root as the longest crown root)

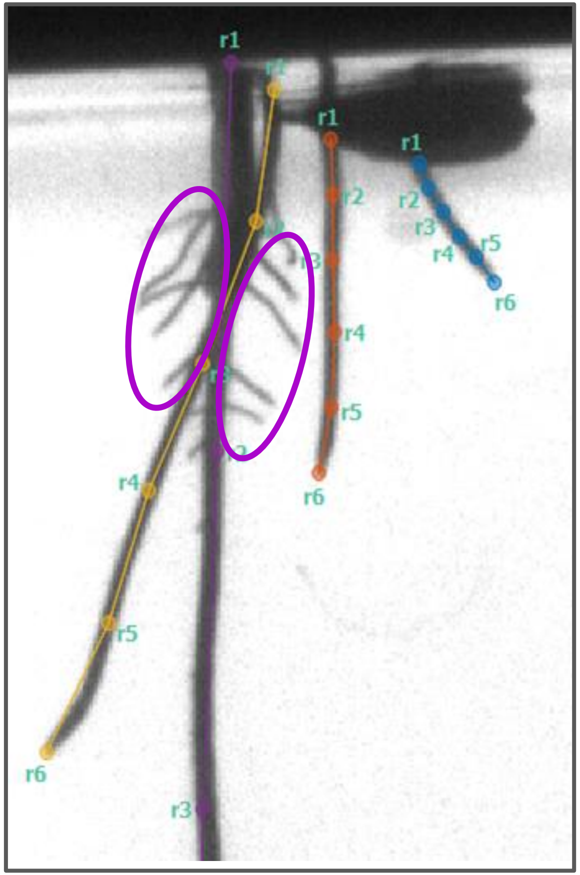

General Rice Labeling Rules: Key Ideas

Save frequently

- Changes are NOT automatically saved

Consistent labels

- if labels are not consistent, they will have to be re-done
- Go slowly and carefully rather than quickly
- If more than one person is labeling, review rules for crop and experiment beforehand and record who labels which replicates

Don’t over label

- Older roots are very complicated
    - Review specific experiment focus to decide priorities but in general prioritize:
        - larger roots
        - more visible roots
        - roots most important to overall root system architecture
- Minimize crisscrossing labels

General Rice Labeling Rules: Skeleton of Rice

- Roots in both rice models are labeled using 6-node trees, which form a line
  - Nodes should be equally spaced lengthwise along each root.
  - Nodes should be centered widthwise on the root.
    - Zoom in to make sure they're centered

"node_names": ["r1", "r2", "r3", "r4", "r5", "r6"],
"edge_inds": [[0, 1], [1, 2], [2, 3], [3, 4], [4, 5]]

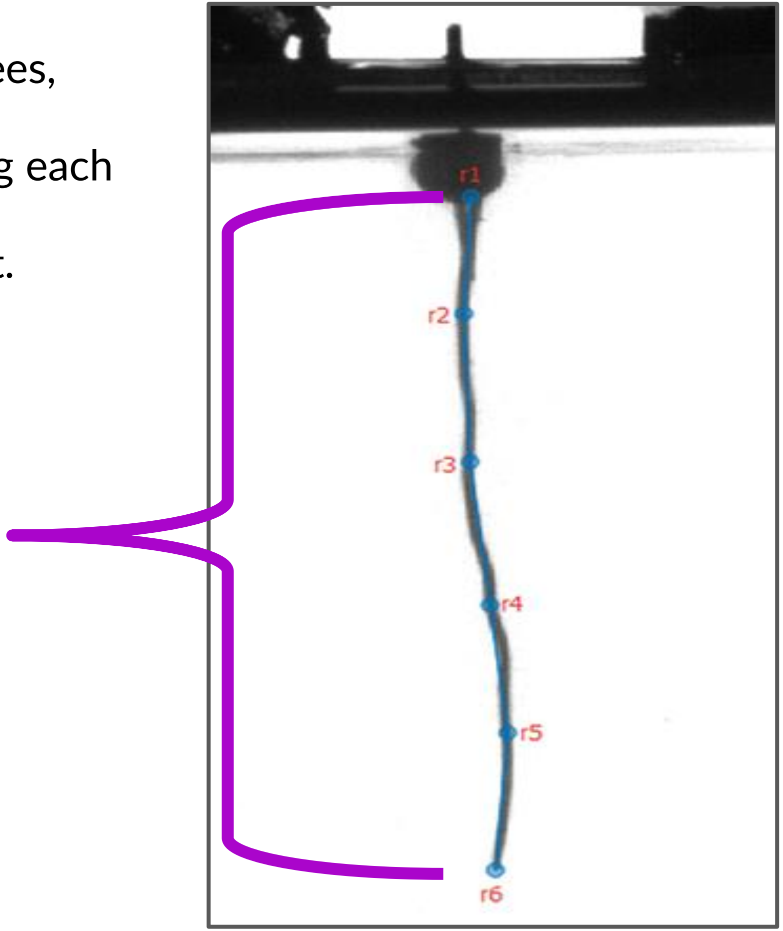

General Rice Labeling Rules: Tip Definition

- The tip of the root is the last visible point, and should be labelled with r6 (circled in purple)
    - Remember to zoom in for precision

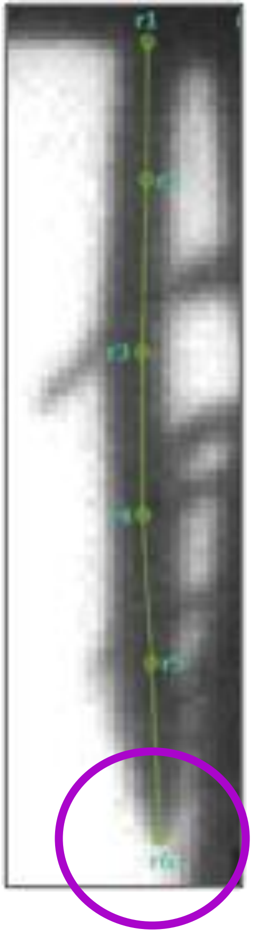

General Rice Labeling Rules: Base Definition

- The **base** of the root is the **first visible point** on an individual root and should be labelled as **r1**.
    - If the bases of two roots overlap, r1 on one of the roots should be moved down to the first distinguishable point of the root that does not overlap with another root.
    - The bases of thicker and longer roots should be prioritized and kept higher instead of moved down.

General Rice Labeling Rules: Base Definition Examples

Incorrect base labelling:

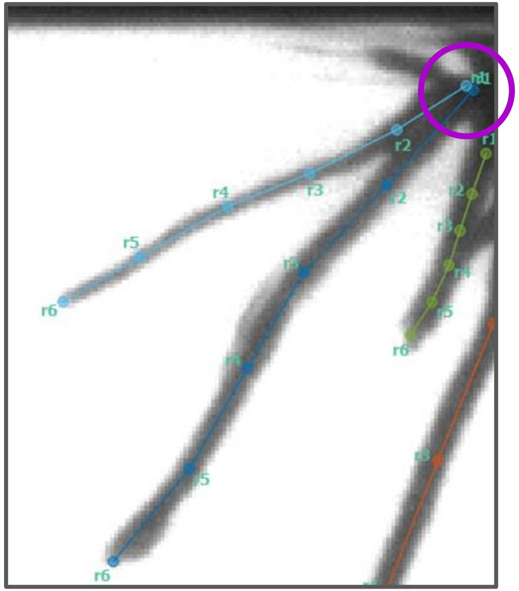

Correct base labelling:

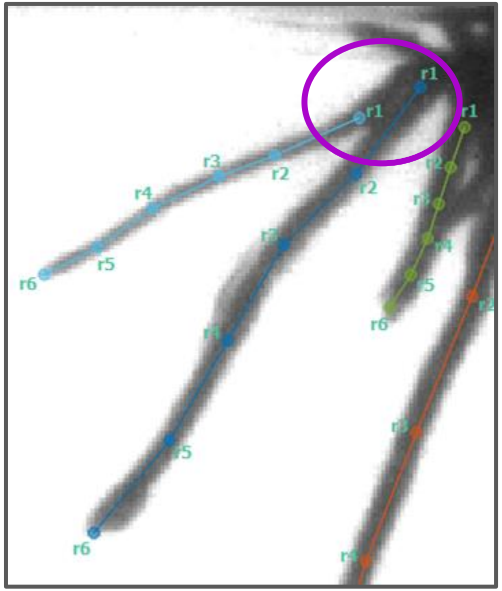

- Although the bases of both roots labeled in blue overlap, their r1 points should not be next to each other as in the left image. The thicker should be prioritized and the r1 point of the thinner root should move downwards to the first non-overlapping section as in the right image .

General Rice Labeling Rules: Base Definition Examples

- Another example of labeling r1 lower in the case of overlap and prioritizing the longer/thicker root.

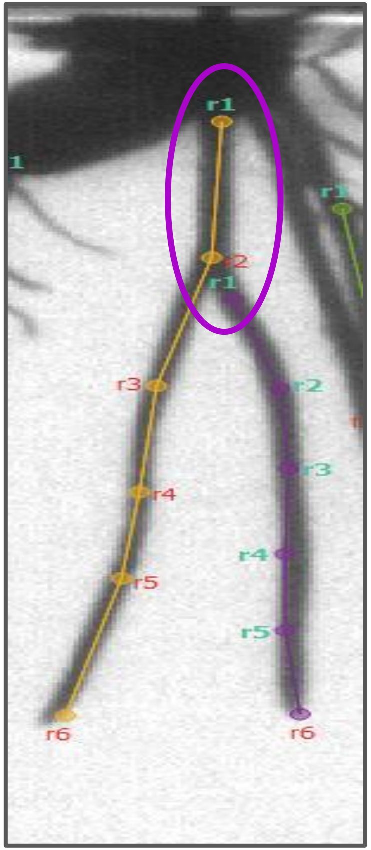

General Rice Labeling Rules: Prioritizing Roots

- If two roots have similar thickness/length, prioritize the one that is less obstructed by other roots
  - The non-prioritized one will be prioritized in a different frame
- Example in crown root labeling to the right:
  - Yellow and orange roots are around same length/thickness
  - yellow root is obstructed by the purple root and its base is less visible than the orange base
  - So orange root should be prioritized in this frame
    - Base of yellow is moved down

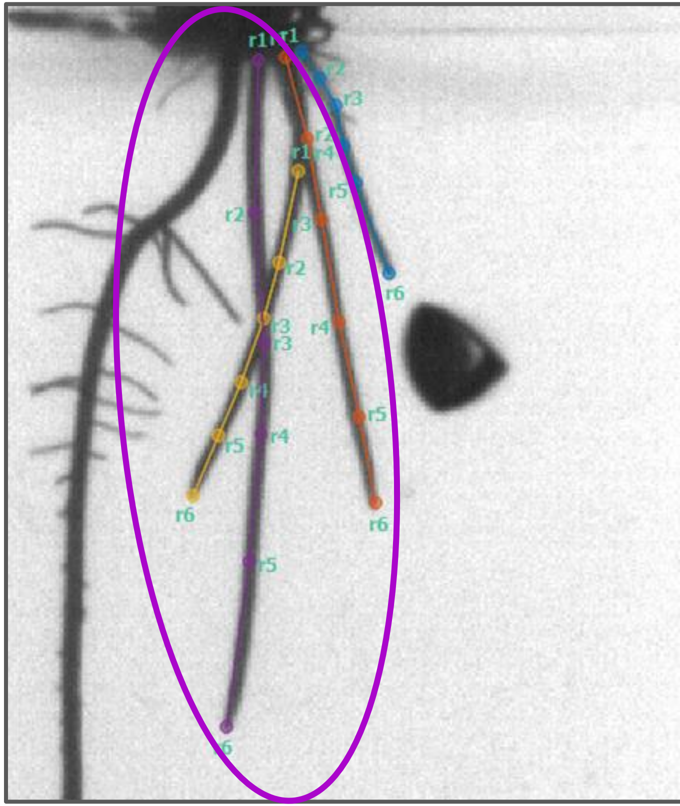

General Rice Labeling Rules: Occluded Small Roots

- If root is small and 75% or more occluded by another larger root, don't label (circled in purple)

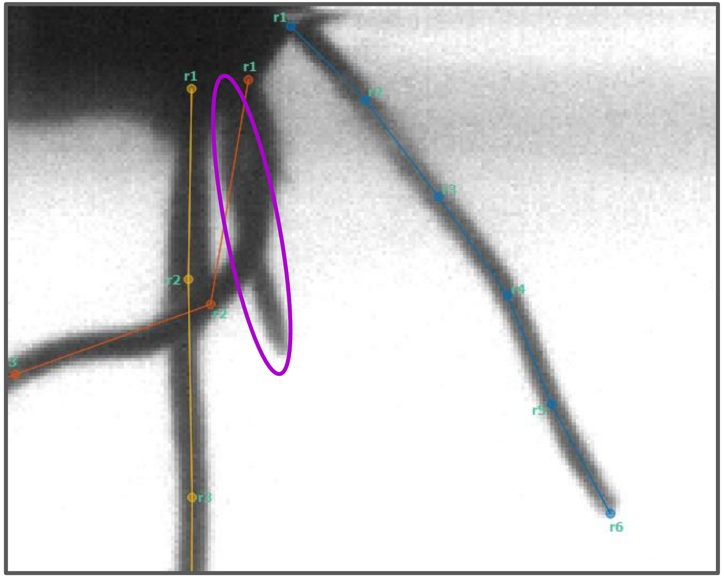

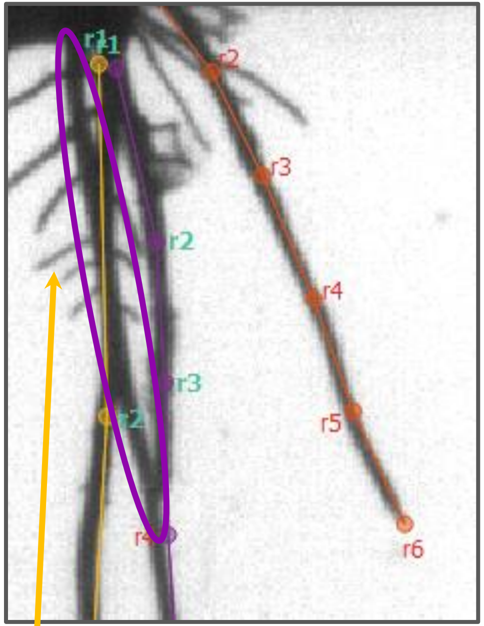

Hairy roots that shouldn't be labelled

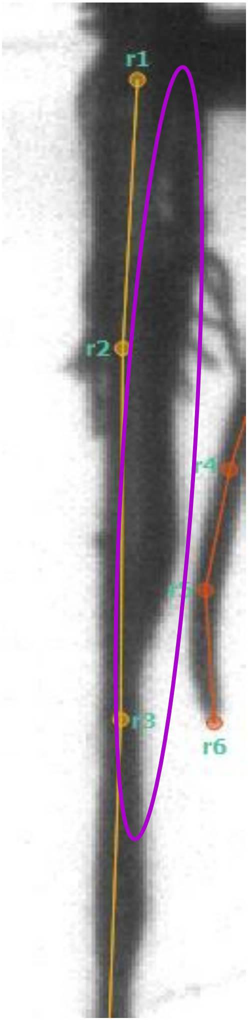

General Rice Labeling Rules: Toggling Visibility - Bases

- Use sparingly, only when any of following conditions are met:
  - Toggle visibility of a node anytime it’s on a part of the root that’s not visible.
  - If two roots’ bases overlap, you can either move the base of the lower-priority root down (TRY THIS FIRST), or toggle the visibility of the nodes above its first non-overlapping point
    - For an example of moving r1 downwards, see base definition/labelling slide

General Rice Labeling Rules: Toggling Visibility - Bases Example

- The bases of the two rightmost roots overlap
- Since the purple root is both thicker and longer, it takes priority, the visibility of r1 of the red root should be toggled to invisible, and r2 should be placed at the first non-overlapping point on the red root.
- Use right click to toggle visibility.
- Toggled invisibility marked by smaller node dot and node name in grey

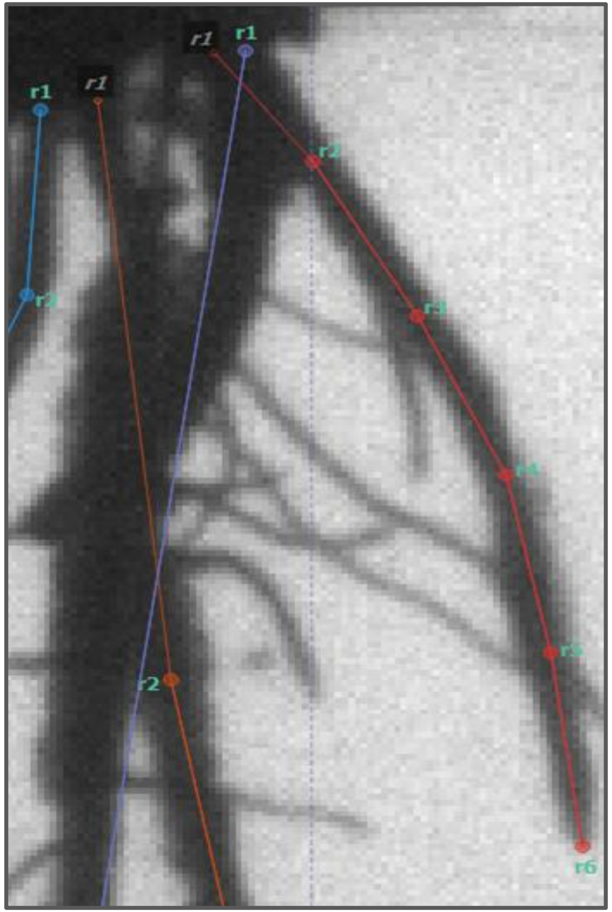

General Rice Labeling Rules: Toggling Visibility - Tips

- SLEAP uses landmark detection
  - ==> Tips are easily recognizable
- If you cannot see the tip, label the last point on the root as r5 and r6 as invisible
- Only do this if most of that root is visible (more than 75%). Otherwise, just do not label the root.

- In the example to the right the tip of the right most blue labeled root is occluded so r5 is on the last visible part of the root and r6 is toggled invisible

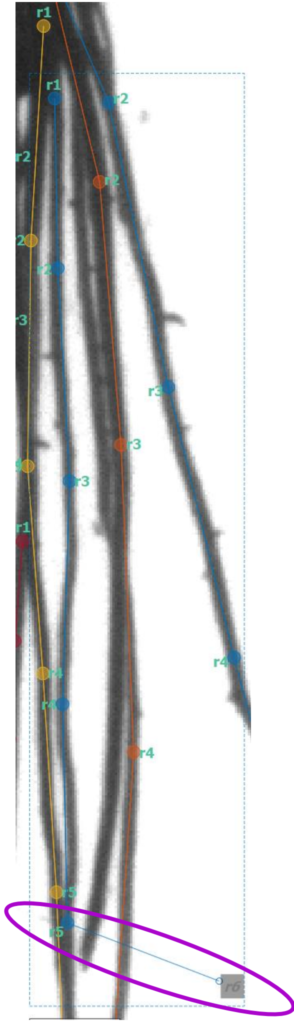

General Rice Labeling Rules: Toggling Visibility - Holes

- There cannot be any holes of visibility in a root.
- If a segment in the middle of a root is not visible, the nodes that are in the occluded part must be toggled invisible AND the rest of the nodes going one direction (towards r1 or towards r6) must also be toggle invisible
- Example on the right: r3 on the red root overlaps with the pink root, and in this case the pink root takes priority.
  - r3 on the red root must be toggled invisible.
  - Since it's in the middle of the root, either r4, r5, and r6 or r1 and r2 must also be toggled invisible to avoid a hole.

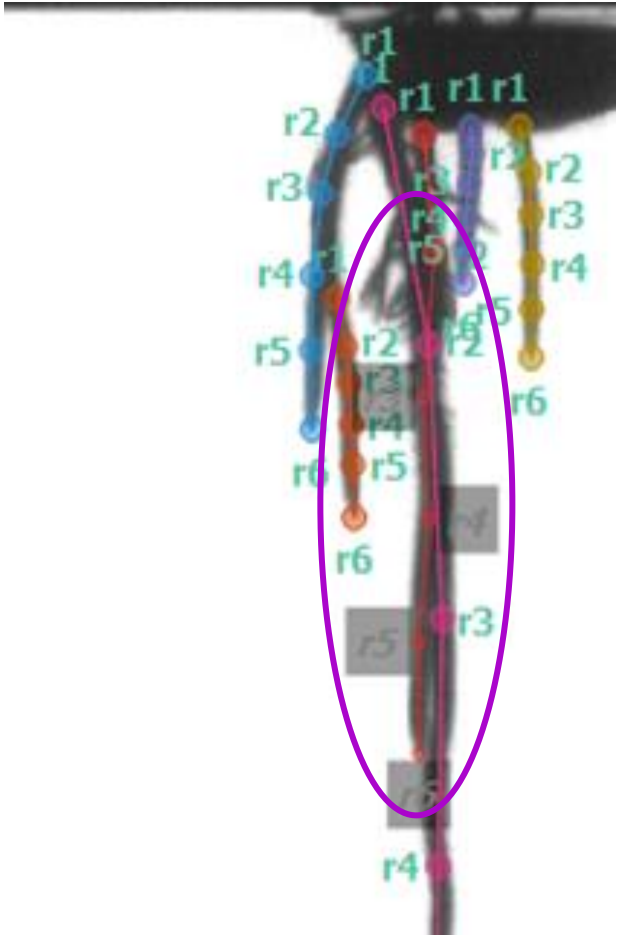

General Rice Labeling Rules: Labeling Around Bubbles

- If a bubble is obstructing a root but you're sure the root goes under the bubble, place a node on top of the bubble
  - Make sure to keep nodes evenly spaced
  - If you're not sure where the root ends/if it ends behind the bubble, shift to other frames with a similar angle where the root isn't hidden behind the bubble

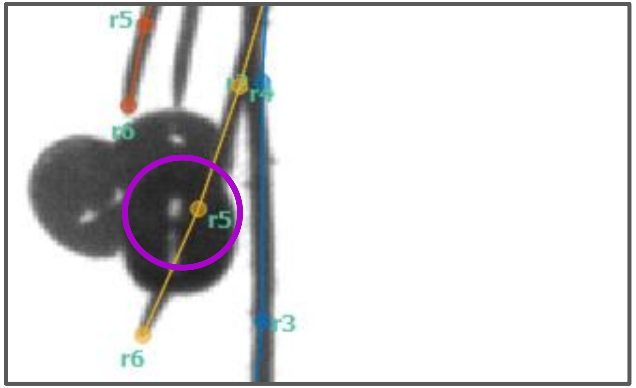

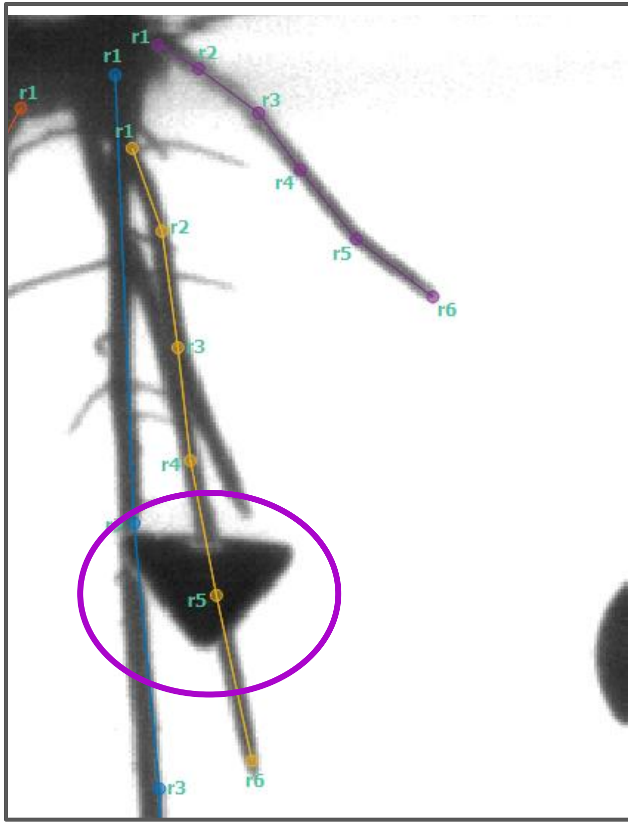

General Rice Labeling Rules: Shoots

- This is a shoot! Do not label it.
- You can tell it is an upside-down shoot because it has sharp edges and it folds when it hits the bottom
- Label the roots only!

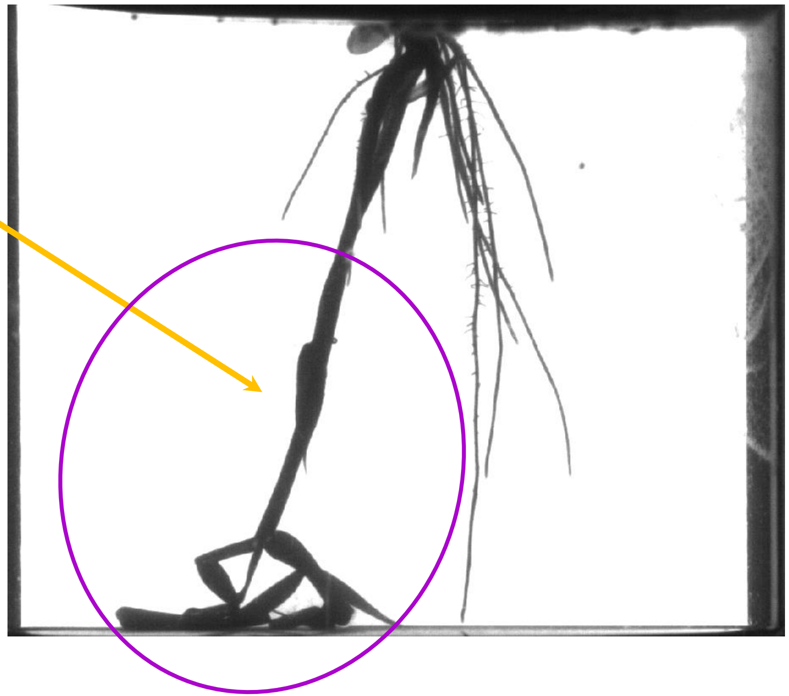

Soybean Quality Control and Labeling in SLEAP

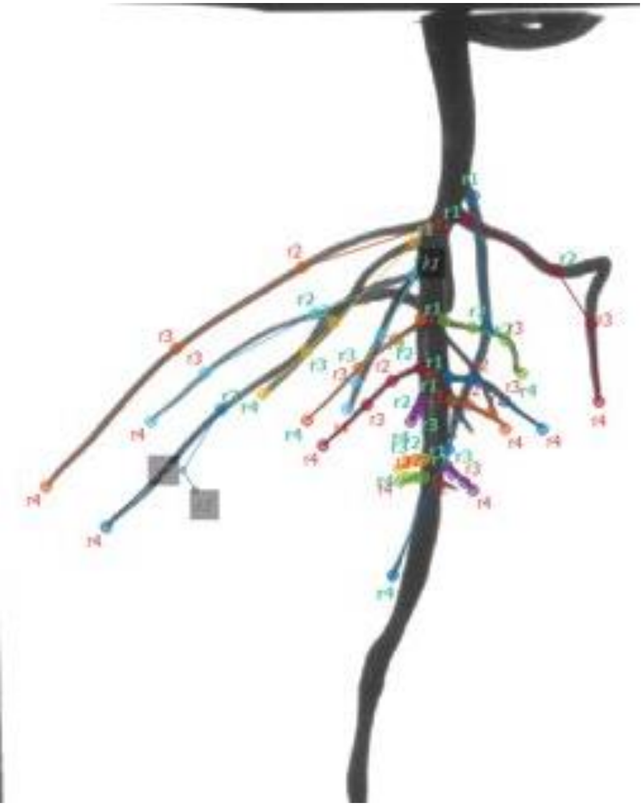
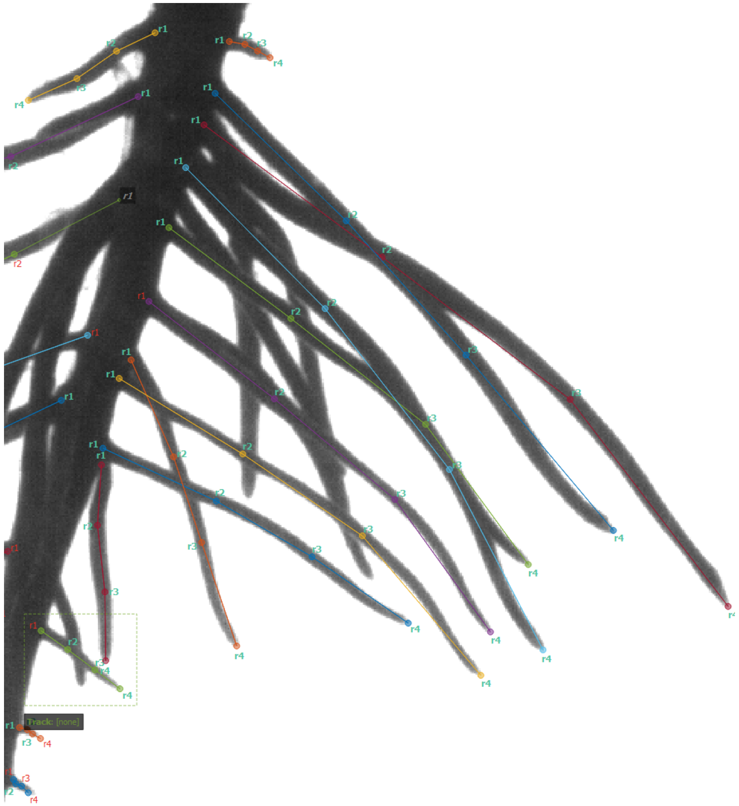
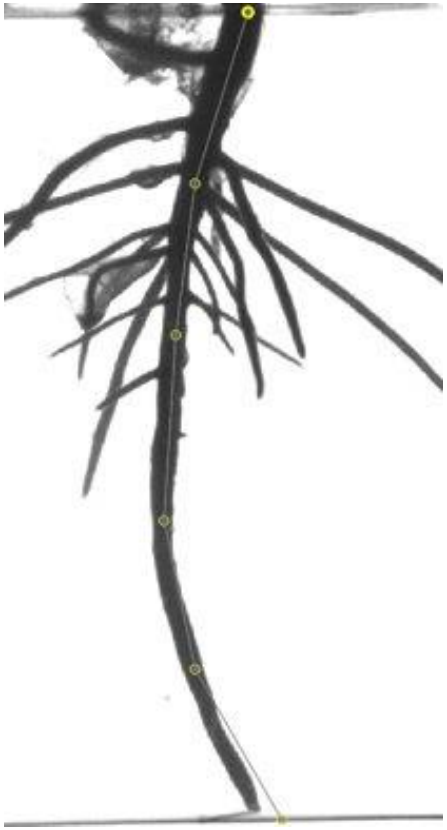

Overview

- **Quality control**
  - Protocol
  - Codes
  - Examples
- **Soybean Models**
  - Primary Root Model and skeleton
  - Lateral Roots Model and skeleton
- **General Soybean Labeling Rules**
  - Key Ideas
  - Tip and Base Definition
  - Prioritizing Roots
  - Occluded, Small Roots
  - Toggling Visibility: Bases, Tips, Holes
  - Labeling Around Bubbles and Contamination

SLEAP Quality Control (QC) Protocol

- Before proofreading in sleap, QC must be done
- Meet beforehand to discuss specific QC requirements for individual experiments
  - Make sure there is consensus between everyone involved in QC and ideally do it together
- Do QC on the final day scan
  - If you do this before the final scan, please write the age you QC'd plant in column "QC_age"
- Do QC based on QC codes on following slide
- If replicate passes quality control, record a 0 in the "QC" column in the master data sheet for the experiment and leave "QC_code" column blank
- If replicate does not meet quality control requirements and needs to be excluded, mark 1 in "QC" column and record corresponding QC code in "QC_code" column

QC_code	Description	Notes/Details (vary based off of experiment)
**cont**	Excessive contamination engulfing plant	• Plants can have some contamination and not be QC'd out as long as you can see most of the root not contaminated in some frames<br>• As long as base of root is not contaminated, include plant (don't QC)
**sub**	Whole plant submerged	
**jig**	Media jiggly	
**ori**	Incorrect orientation	• Careful: distinguish poor growth and orientation, growing sideways or up is **ori** not **pg**
**germ**	Poor germination	• No germination or just a cotyledon nub grown (any growth past cotyledon nub is **pg** not **germ**)
**dead**	Unhealthy or dead	
**adv**	Too many adventitious roots	• Adventitious roots are different from primary or lateral roots. Models are not trained on adventitious roots so too many can result in error.
**miss**	Cylinder missing	• Only use if you can't find the scans for the barcode (naming individual cylinder). If you need to throw away a cylinder in the middle of an experiment note the date and QC_code
**pg**	Poot growth	• In experiments where we expect to see no grwth we will be less strict with this/define new conditions

QC Examples: **cont** (Contamination)

- Contamination engulfs base of plant
- Contamination occludes large portion of roots on most frames

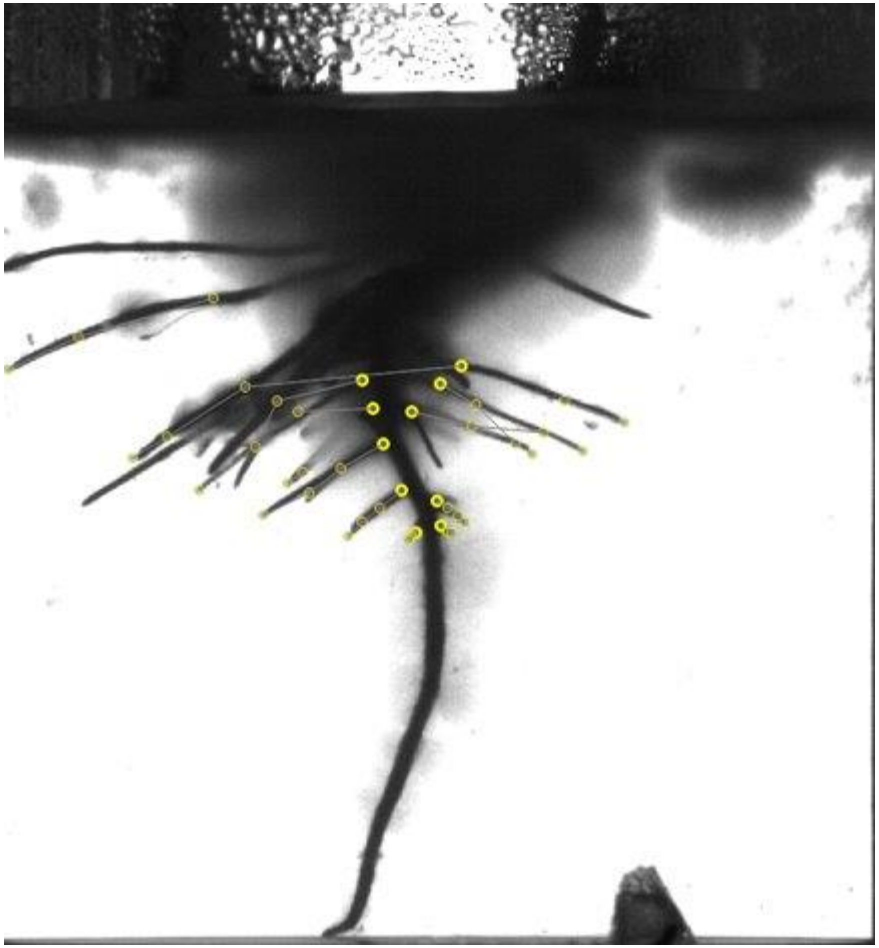

QC Examples: **pg** (poor growth)

- Example to the right is also a **cont** QC
- Plant is very small for timepoint in comparison to other individuals of the same genotype and environmental conditions
- Plant looks stunted for soybean

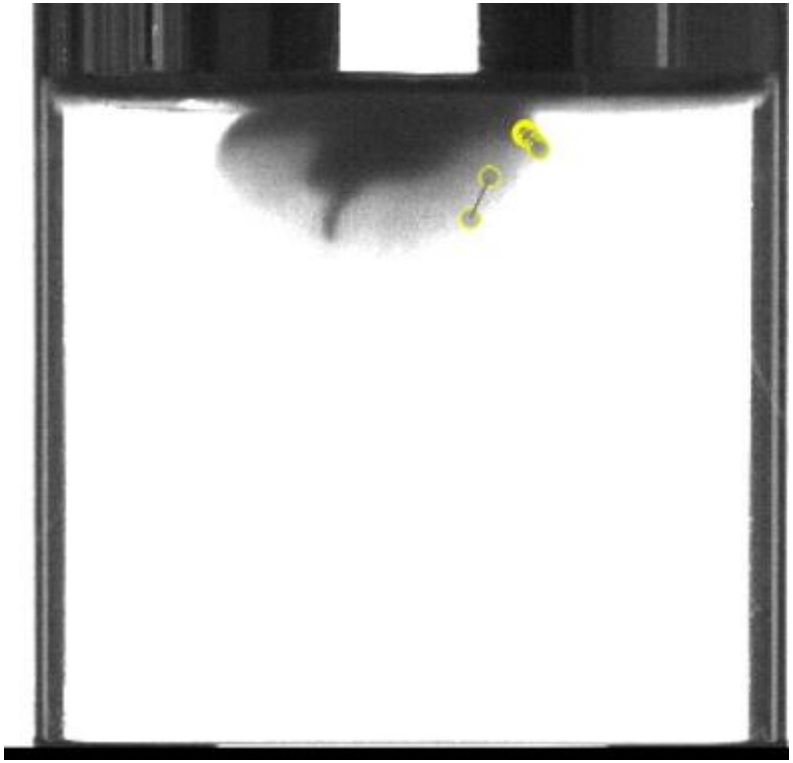

Soybean Models

Primary Root Model
Lateral Root Model

- Soybean is a dicot, it can have one primary root and multiple lateral roots
- Soybean has two SLEAP models one for primary roots and one for lateral roots
  - For each model, we need to label different roots in the rice root system architecture (RSA)

Soybean Models: Primary Root Model and Skeleton

- In Soybean, primary roots are usually the largest, thickest, most gravitropic root.
- Primary roots grow out of the seed whereas lateral roots grow out of the primary root

- Primary roots are labelled using 6-node trees, which form a line
- r1 is the base node and r6 is the tip node
- Nodes should be equally spaced lengthwise along each root.
- Nodes should be centered widthwise on the root.
- Zoom in to make sure they're centered

"node_names": ["r1", "r2", "r3", "r4", "r5", "r6"],
"edge_inds": [[0, 1], [1, 2], [2, 3], [3, 4], [4, 5]]

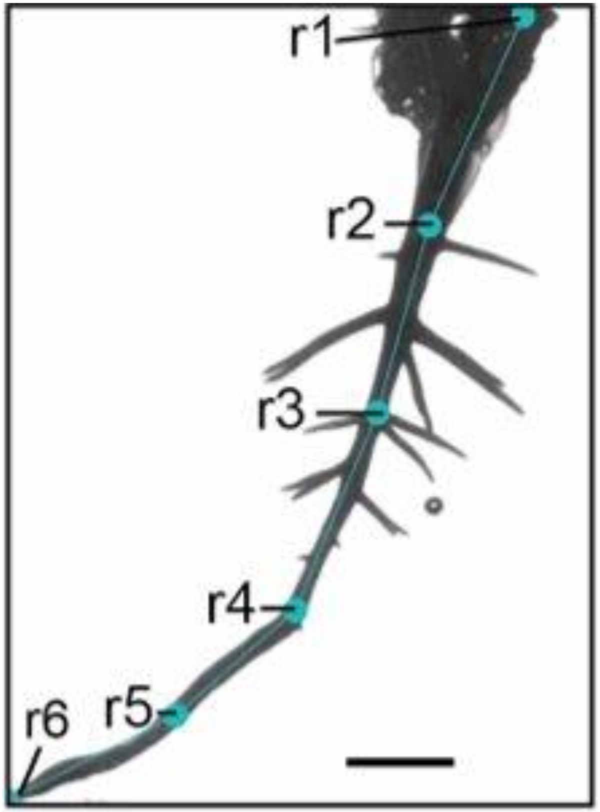

Soybean Models: Lateral Roots Model and Skeleton

- Lateral roots grow out of the primary root and in general are thinner and shorter than the primary root at this age

- Lateral roots are labelled using 4-node trees, which form a line
- r1 is the base node and r4 is the tip node
- Nodes should be equally spaced lengthwise along each root.
- Nodes should be centered widthwise on the root.
- Zoom in to make sure they're centered

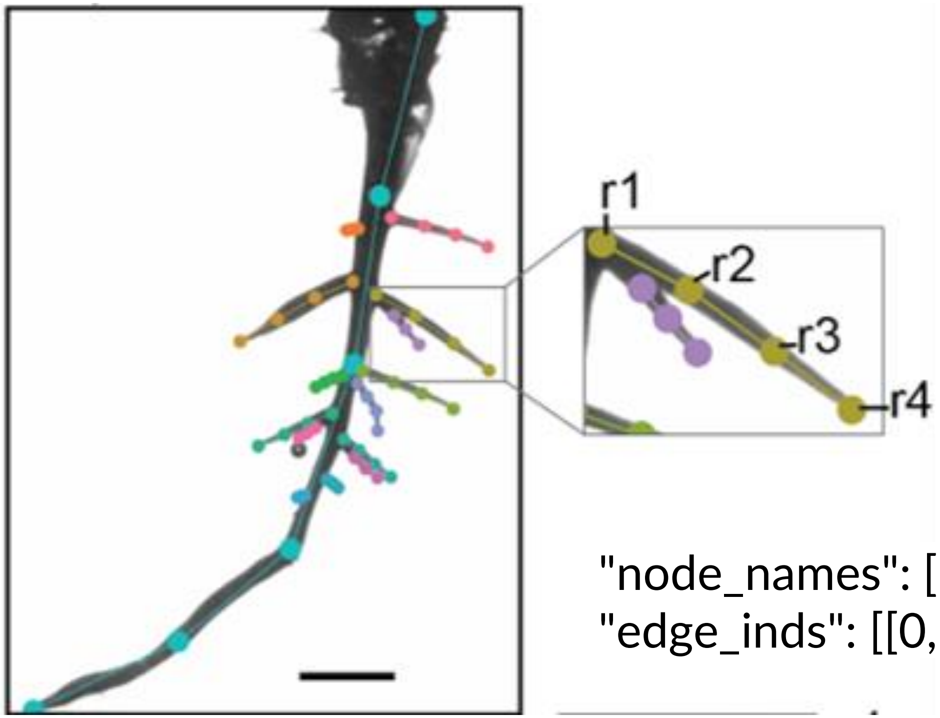

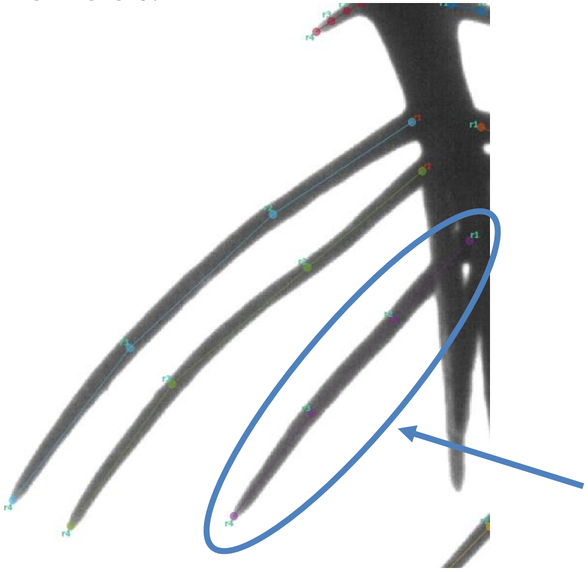

one instance or lateral root

"node_names": ["r1", "r2", "r3", "r4“]
"edge_inds": [[0, 1], [1, 2], [2, 3]]

General Soybean Labeling Rules: Key Ideas

Save frequently

- Changes are NOT automatically saved

Consistent labels

- if labels are not consistent, they will have to be re-done
- Go slowly and carefully rather than quickly
- If more than one person is labeling, review rules for crop and experiment beforehand and record who labels which replicates

Don’t over label

- Older roots are very complicated
  - Review specific experiment focus to decide priorities but in general prioritize:
    - larger roots
    - more visible roots
    - roots most important to overall root system architecture
- Minimize crisscrossing labels

General Soybean Labeling Rules: Tip Definition

- The tip of the root is the last visible point, and should be labelled with r4 in the lateral root model and r6 in the primary root model (primary root example to the right) .
  - Remember to zoom in for precision

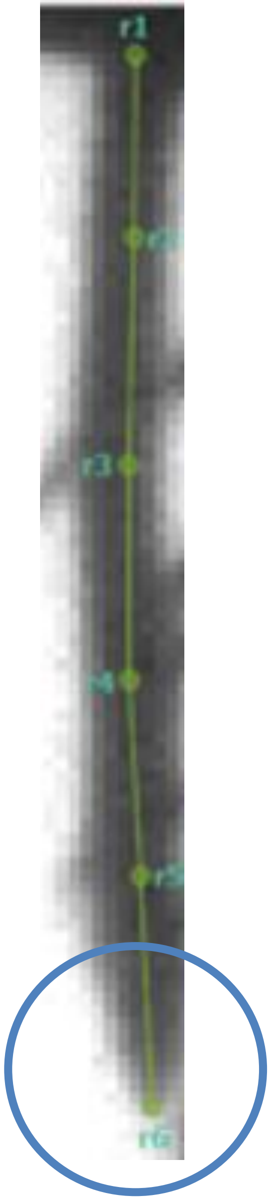

General Soybean Labeling Rules: Base Definition

- The **base** of the root is the **first point visible** on a primary or a lateral root and should be labelled as **r1**.
- **For a primary root**, this point is where the primary root reaches the seed or the top most visible part of the root structure.
- **For a lateral root**, this point is where the lateral root meets the primary root
- If the bases of two roots overlap, r1 on one of the roots should be moved down to the first distinguishable point of the root that does not overlap with another root.
  - Or toggle r1 invisible
- The bases of thicker and longer roots should be prioritized and kept higher instead of moved down.

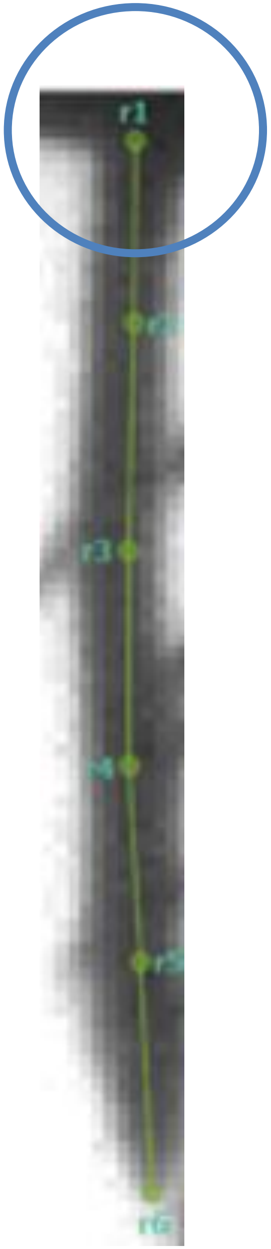

General Soybean Labeling Rules: Base Definition Examples

- Don't put nodes right next to each other
- The probability fields used to find them will overlap too much.
- You can move the green base node a bit so that it is on its own root
- Or mark as invisible if too occluded to tell where the base is

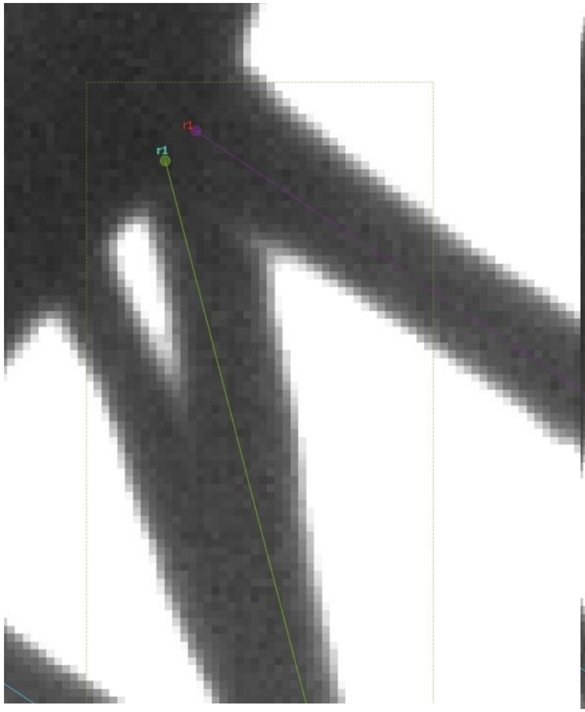

Incorrect

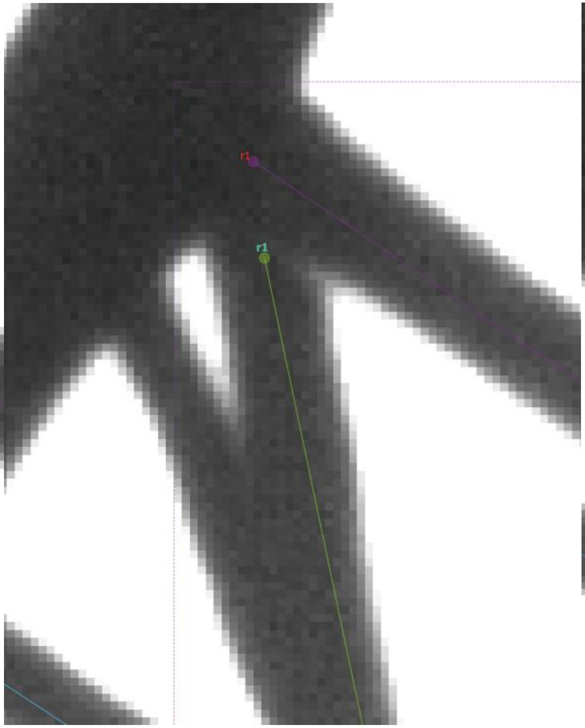

Correct

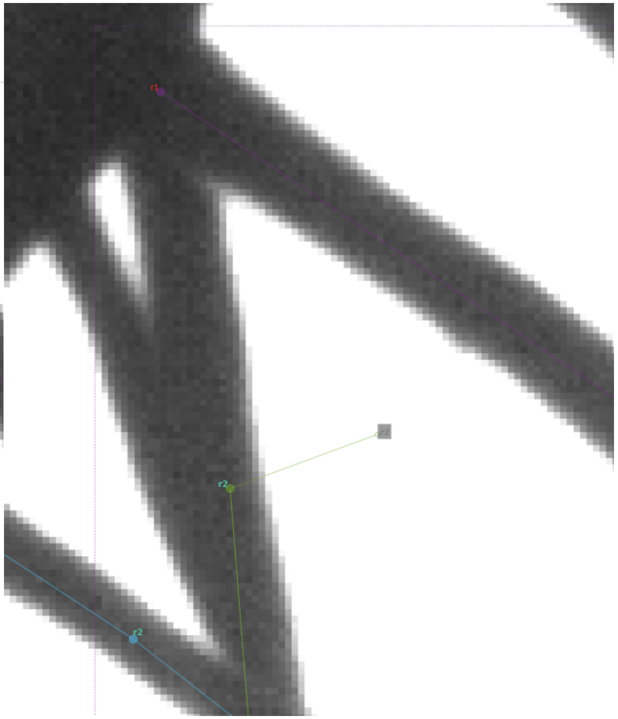

Correct

General Soybean Labeling Rules: Prioritizing Roots - Size

- Prioritize larger roots
- If two roots have similar thickness/length, prioritize the one that is less obstructed by other roots
  - The non-prioritized one will be prioritized in a different frame

- The green root is more important than the purple root. Label the green root first and the purple root after.
- Since you cannot see the base of the purple root after the green root is labeled, mark the base of the purple root as invisible (by **right-clicking** it).

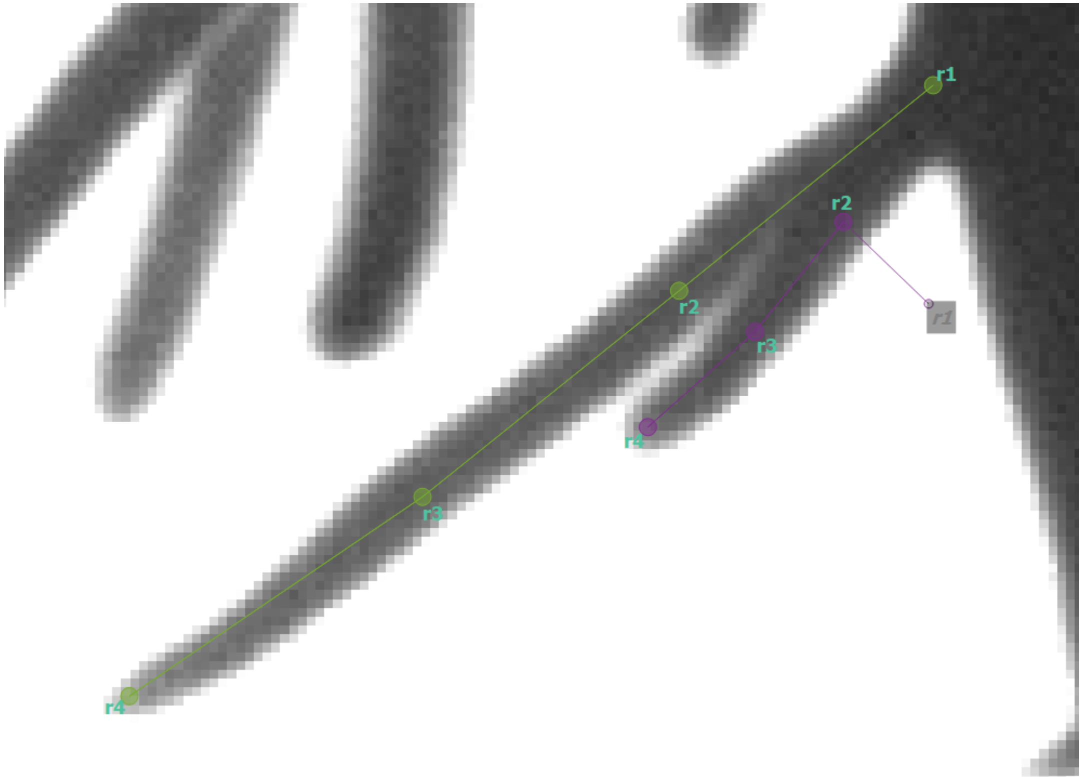

General Soybean Labeling Rules: Prioritizing Roots - Angle

- Prioritize root with the true angle
  - Every frame shows a different angle of the plant
  - Roots viewed from the side have a smaller angle than viewed from the front
  - We want the max angle over the 72 frames

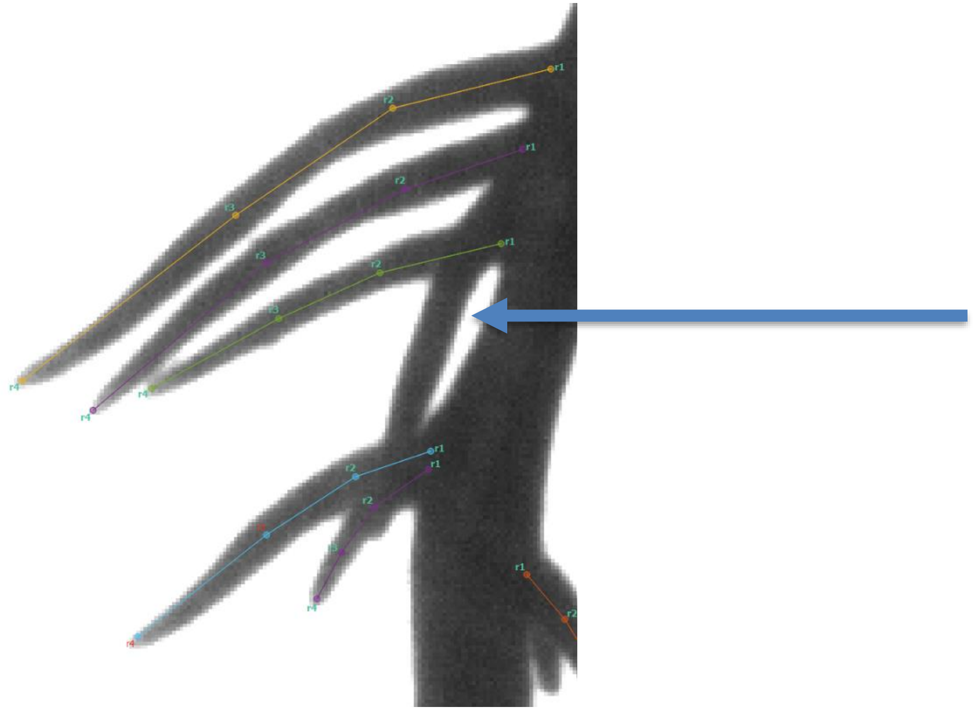

Root not labeled because not true angle in this frame

General Soybean Labeling Rules: Occluded and Small Roots

- Don’t label a root if it’s 75% or more occluded by another root

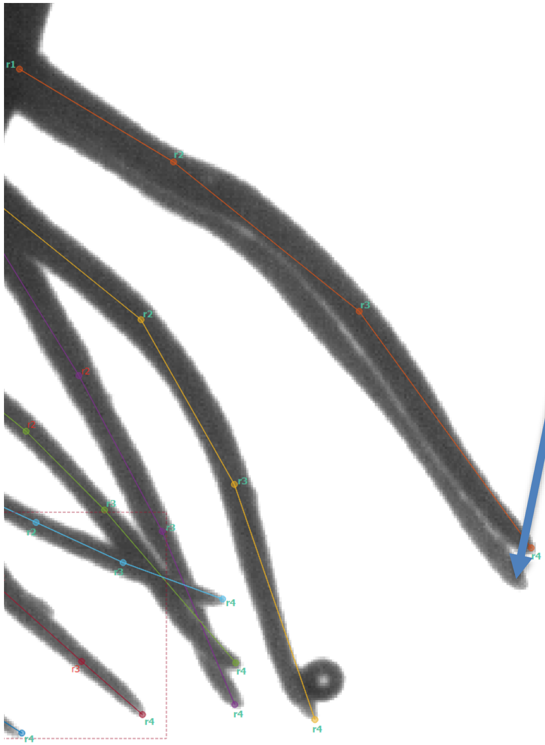

- label the base of an occluded root if you can tell where that would be
- Here the occluded root is not labeled so it's ok to label the green root's base

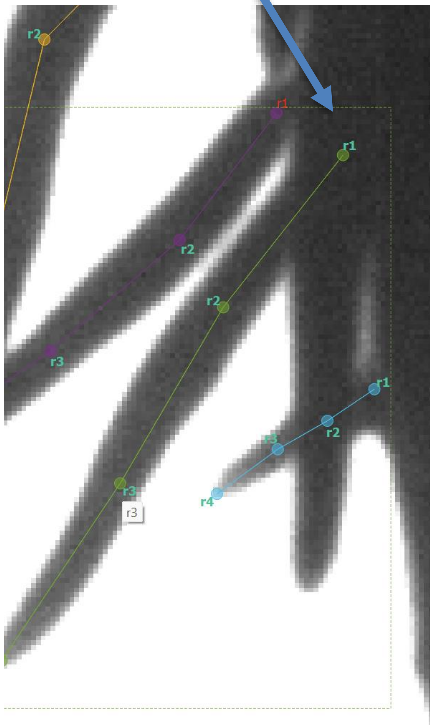

General Soybean Labeling Rules: Toggling Visibility - Bases

- Use sparingly, only when any of following conditions are met:
- Toggle visibility of a node anytime it's on a part of the root that's not visible.
- If two roots' bases overlap, you can either move the base of the lower-priority root down (TRY THIS FIRST), or toggle the visibility of the nodes above its first non-overlapping point
- For an example of moving r1 downwards, see base definition/labelling slide

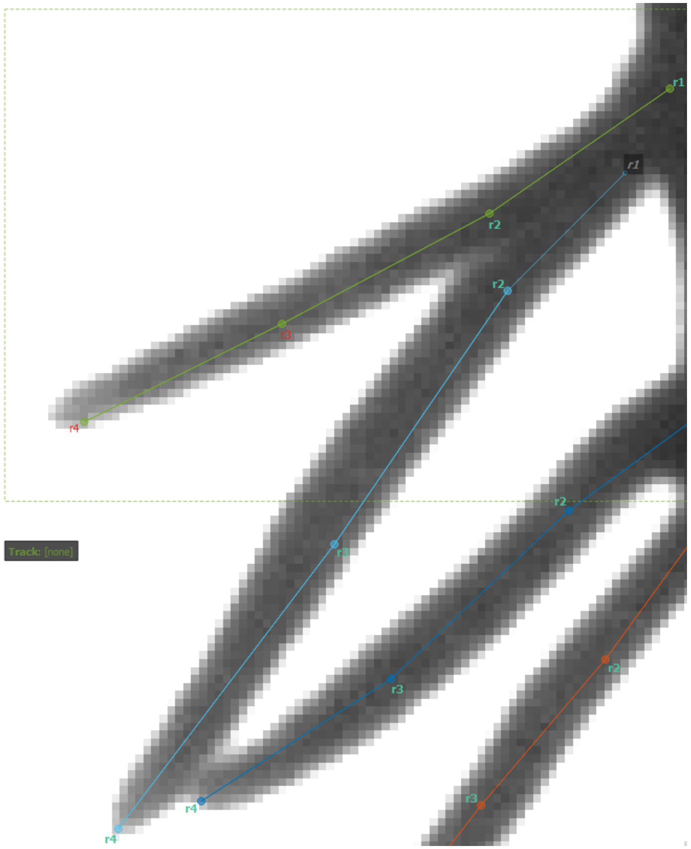

The light blue root base is behind the green root.

➔Toggle the base of the light blue root invisible by **right –clicking it**

It will then appear grey

General Soybean Labeling Rules: Toggling Visibility - Tips

- SLEAP uses **landmark detection**
  - ==> Tips are easily recognizable
- **For primary roots**, If you cannot see the tip, label the last point on the root as r5 and r6 as invisible
- **For lateral roots**, If you cannot see the tip, label the last point on the root as r3 and r4 as invisible
- Only do this if most of that root is visible (more than 75%). Otherwise, just do not label the root.

General Soybean Labeling Rules: Toggling Visibility - Holes

- There cannot be any holes of visibility in a root.
- If a segment in the middle of a root is not visible, the nodes that are in the occluded part must be toggled invisible AND the rest of the nodes going one direction (towards r1 or towards r6) must also be toggle invisible

General Soybean Labeling Rules: Labeling Around Bubbles and Contamination

- If a bubble is obstructing a root but you're sure the root goes under the bubble, place a node on top of the bubble
- Make sure to keep nodes evenly spaced
- If you're not sure where the root ends/if it ends behind the bubble, shift to other frames with a similar angle where the root isn't hidden behind the bubble
- See example of node placed over bubbled circled in blue to the right

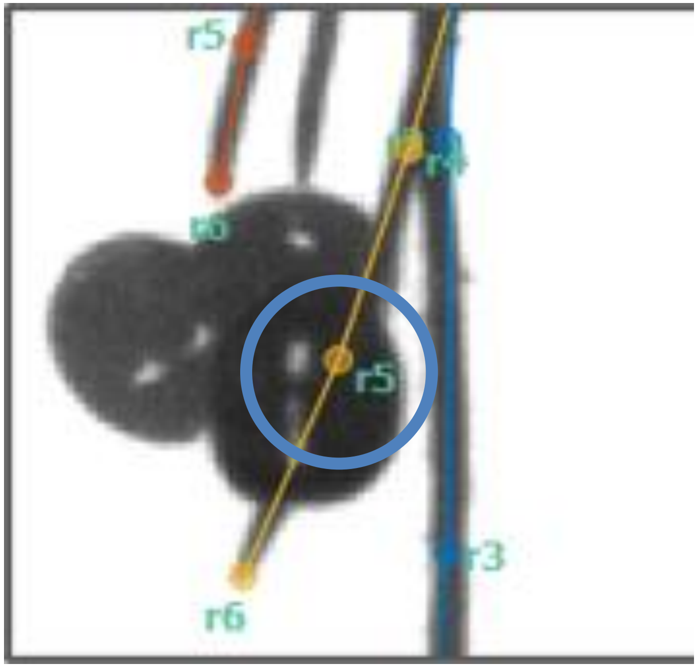

Supplement: Supplementary 1 — Figs. S1 and S2 Tables S1 to S6 Annotation Protocols [file plantphenomics.0175.f1.zip › supp-annotation_protocols.pdf]
